# Quantifying how HIV-1 envelope sequence features impact vaccine efficacy in the HVTN 705/HPX2008 randomised trial in southern African women

**DOI:** 10.1016/j.ebiom.2026.106459

**Published:** 2026-08-31

**Authors:** Michal Juraska, Li Li, Craig A. Magaret, James Peng, Elena E. Giorgi, James Ludwig, Lindsay N. Carpp, Allan C. deCamp, Jia J. Kee, Cindy Molitor, Marthin Mandig, Paul T. Edlefsen, Raabya Rossenkhan, Dylan H. Westfall, Wenjie Deng, Lennie Chen, Hong Zhao, Bruna Galvao, Craig Adams, Anna Yssel, David Matten, Talita York, Asanda Gwashu-Nyangiwe, Nonkululeko Ndabambi, Ruwayhida Thebus, Edith M. Swann, John A. Hural, Janine van Duijn, Susan Buchbinder, Frank Tomaka, Lawrence Corey, Kathryn Mngadi, Glenda E. Gray, Peter B. Gilbert, Carolyn Williamson, James I. Mullins

**Affiliations:** a Vaccine and Infectious Disease Division, Fred Hutchinson Cancer Center, Seattle, WA, 98109, USA; b Department of Biostatistics, University of Washington, Seattle, WA, 98195, USA; c Department of Microbiology, University of Washington School of Medicine, Seattle, WA, 98195, USA; d Institute of Infectious Disease and Molecular Medicine and CIDRI-Africa Wellcome Centre, Department of Pathology, Faculty of Health Sciences, University of Cape Town and National Health Laboratory Service, Cape Town, 7701, South Africa; e Division of AIDS, National Institute of Allergy and Infectious Diseases, Bethesda, MD, 20852, USA; f Janssen Vaccines & Prevention BV, Leiden, 2333, the Netherlands; g San Francisco Department of Public Health, San Francisco, CA, 94102, USA; h Janssen Research & Development, LLC, Titusville, NJ, 08560, USA; i Department of Medicine, University of Washington School of Medicine, Seattle, WA, 98195, USA; j Department of Laboratory Medicine, University of Washington School of Medicine, Seattle, WA, 98195, USA; k Public Health Sciences Division, Fred Hutchinson Cancer Center, Seattle, WA, 98109, USA; l The Aurum Institute, Johannesburg, 1609, South Africa; m South African Medical Research Council, Cape Town, 7505, South Africa

**Keywords:** AlphaFold3, CD4 binding site signature regions, Heterologous Ad26.Mos4.HIV and clade C gp140 vaccine regimen, Sieve analysis, Subtype C broadly neutralising antibody resistance signature positions, Physicochemical-weighted Hamming distance

## Abstract

**Background:**

A heterologous Ad26.Mos4.HIV and clade C gp140 vaccine regimen did not show overall significant efficacy against HIV-1 acquisition [point estimate 14.1%; 95% confidence interval (CI), −22.0 to 39.5] in the HVTN 705/HPX2008 trial in southern African women. We examined whether and how vaccine efficacy (VE) against HIV-1 diagnosis over 7–24 months post-first dose varied by HIV-1 Envelope (Env) amino acid sequence features.

**Methods:**

HIV-1 viral sequences were generated by PacBio SMRT-UMI sequencing from the first RNA-positive sample of participants who acquired HIV-1. Env amino acid sequence features were prespecified for analyses based on 1) being hypothesised to impact VE; and 2) having sufficient variability. Sieve analyses assessed VE by a single representative sequence and by viral population composition.

**Findings:**

The majority of Env features showed no evidence of differential VE, with only two signals having familywise error rate (FWER) P-values <0.10. In single-sequence analyses, VE declined with increasing physicochemical-weighted Hamming distance from the C97ZA vaccine insert in clade C broadly neutralising antibody resistance-associated signature positions (FWER P = 0.08). Sequence-predicted Env structural features showed no significant vaccine vs. placebo differences in structural divergence from the C97ZA vaccine-insert Env sequence. In multi-sequence analyses (median 121 sequences/individual), VE was higher against viral populations with ≥99% vs. <99% L832 prevalence (VE = 91.7%; 95% CI, 67.4–97.9 vs. VE = −7.0%; 95% CI, −55.5 to 26.4) (unadjusted P = 0.0002 for differential VE, FWER P = 0.023).

**Interpretation:**

Despite extensive prespecified and exploratory analyses including Env features supported by prior studies to potentially impact VE, there was only limited, weak evidence that Env sequence features modified VE in HVTN 705. Although previous work suggested a protective role of IgG3 binding to V1V2 in a small subgroup of vaccine recipients, a V1V2 sieve signal was absent.

**Funding:**

National Institutes of Health and Johnson & Johnson.

**Research in context**

**Evidence before this study**

We queried PubMed on October 8, 2025 using the following combinations: (“sieve analysis” and “HIV-1”), (“HIV-1 vaccine” and “signature site”), and (“HIV-1 vaccine” and “genetic signatures”). We included research articles from database inception through to the day of search and did not use any filters or place any restrictions on language in our search. Through these three searches, we identified 30 unique articles, including 3 reviews/commentaries. Among the identified research articles were five sieve analyses of four different preventive randomised, placebo-controlled HIV-1 vaccine efficacy trials. One of these analyses reported that, for a DNA/recombinant adenovirus type 5 vector HIV-1 vaccine that had shown no overall efficacy to reduce HIV-1 acquisition in the HVTN 505 trial, intra-host Gag, Pol, Vif, and gp120 diversity was significantly lower, and gp120 sequences were significantly more distant from the vaccine insert, in vaccine versus placebo recipients. Specific amino acid sites implicated in these signals mapped to the CD4 binding site and CD4-induced monoclonal antibody footprints, with these signatures proposed to influence the conformational transition of the HIV-1 envelope trimer between its pre-fusion closed and CD4-bound (open) states. It was not possible to discriminate whether these observed vaccine effects reflected post-acquisition pressure driving viral evolution or differential impact on acquisition of different exposing HIV-1 sequences. We also identified one sieve analysis of two harmonised broadly neutralising monoclonal antibody HIV-1 prevention efficacy trials and a neutralisation sieve analysis of a nonhuman primate simian immunodeficiency virus vaccine trial.

In a second search conducted on the same day, we similarly queried PubMed using the terms (“mosaic HIV-1 vaccine” and “immune correlates”). We identified 10 articles, including 6 reviews/commentaries. Among the identified research articles was an immune correlates analysis of a preventive HIV-1 vaccine efficacy trial of a heterologous Ad26.Mos4.HIV and clade C gp140 vaccine regimen evaluated in southern African females. This study (HVTN 705/HPX2008) found that two immune response markers that were identified as correlates of protection from simian HIV infection in a preclinical nonhuman primate challenge study of a similar mosaic HIV-1 vaccine (potential T-cell epitope responses and gp140-specific IgG binding antibody responses) were not significant correlates of HIV-1 risk in the vaccine efficacy trial. However, a trend was observed for IgG3 binding antibody breadth to V1V2 antigens to correlate inversely with HIV-1 risk, and to also potentially correlate with protection. The search also identified an immune correlates study of the preclinical nonhuman primate challenge study referenced above, which reported that V2-specific binding antibody responses were a correlate of protection from simian HIV infection in nonhuman primates.

**Added value of this study**

The present study performs a sieve analysis of HIV-1 genome sequences from the same preventive HIV-1 vaccine efficacy trial of a heterologous Ad26.Mos4.HIV and clade C gp140 vaccine regimen identified above. Both single-sequence and multi-sequence sieve analyses were performed, which assessed whether and how vaccine efficacy depended on viral sequence features. The latter analyses took full advantage of the rich datasets provided by long-read deep sequencing data and demonstrated that considering viral quasispecies composition in analyses can reveal signals not captured by considering a single representative sequence alone. Additional analyses beyond those performed in previous sieve analyses of vaccine efficacy trials include an AlphaFold3-based deep learning structural similarity sieve analysis based on comparing predicted HIV Envelope (Env) trimer structures from primary endpoint cases to predicted Env trimer structures of each vaccine sequence.

**Implications of all the available evidence**

There was little evidence that Env sequence features modified vaccine efficacy of the heterologous Ad26.Mos4.HIV and clade C gp140 vaccine regimen, or exerted pressure on breakthrough viruses, in the trial's population of southern African females. In particular, the absence of a V1V2 sieve signal provides little sequence-based support for the previously reported IgG3 V1V2 immune correlates signal. Inconclusive signals that merit cautious interpretation and should be regarded as hypothesis-generating include a trend of lower early viral load in vaccine (vs. placebo) recipients, weak CD4bs-related sieve signals, and a quasispecies-level sieve effect at Env position 832 (which lies outside the vaccine Env inserts). The absence of convincing Env-based sieve signals does not preclude vaccine-induced selection operating through other mechanisms, including T-cell-mediated effects. Follow-up work focussing on vaccine-induced T-cell responses and their restriction by HLA alleles is needed to address whether other such sieve effects may have been present.

## Introduction

The global public health burden of HIV remains substantial, with an estimated 40.8 million people living with HIV in 2024, 1.3 million of whom acquired HIV that year.^1^ Some of the highest rates of HIV acquisition are in young women in sub-Saharan Africa, where women and girls (all ages) accounted for 63% of all new HIV acquisitions in 2024.^1^ While remarkable advances have been made in HIV prevention and treatment,^2^ a safe, durably efficacious, and accessible preventative HIV-1 vaccine would play an invaluable role as a complementary tool for ultimately ending HIV globally.^3^^,^^4^ A major hurdle that impedes progress towards this goal is the vast genetic and antigenic diversity of HIV-1.^5^ Mosaic HIV-1 vaccines set out to address this problem by using computationally optimised antigens to maximise potential T-cell epitope coverage for a given viral population of subtypes and recombinant forms.^6^ Multivalent HIV-1 vaccine regimens based on adenovirus 26 (Ad26)-vectored delivery of such mosaic immunogens were shown to confer protection against heterologous SHIV challenge in rhesus monkeys^7^^,^^8^ and to be safe and immunogenic in humans, eliciting broad cellular and humoural responses against diverse HIV-1 variants.^8^, ^9^, ^10^ These results supported the evaluation of a mosaic vaccine regimen in a phase 2b clinical efficacy study, HVTN 705/HPX2008.^11^

HVTN 705 was a randomised, double-blind, placebo-controlled, phase 2b trial conducted at 23 clinical trial sites in Malawi, Mozambique, South Africa, Zambia, and Zimbabwe. The trial enrolled HIV-negative sexually active females aged 18–35 years and in good general health and randomised eligible participants (1:1) to receive vaccine or placebo. Participants in the vaccine group received a heterologous HIV-1 vaccine regimen consisting of mosaic Ad26-based vaccine (Ad26.Mos4.HIV) at Months 0, 3, 6, and 12 and alum-adjuvanted clade C gp140 (C97ZA) at Months 6 and 12. Placebo recipients received saline placebo at Months 0, 3, 6, and 12. Participants were assessed for HIV-1/2 acquisition at Months 0, 3, 6, 7, 9, and every three months thereafter for ≥2 years after enrolment. Primary objective 1 of the study was to evaluate vaccine efficacy (VE) for the prevention of HIV acquisition in HIV-seronegative southern African women. The primary endpoint was a confirmed HIV-1 acquisition diagnosis between the Month 7 and Month 24 visit after the first vaccination in the per-protocol (PP) population. Primary objective 2 of the study was to evaluate the vaccine regimen's safety and tolerability in HIV-seronegative women residing in sub-Saharan Africa. The vaccine regimen did not provide significant protection against HIV-1 acquisition, with estimated VE of 14.1% [95% confidence interval (CI), −22.0 to 39.5] in the primary analysis, and the study was ended after stage 1 (which extended until the last enrolled participant's Month 24 visit) due to the absence of efficacy.^11^

An immune correlates analysis that studied Month 7 immune response biomarkers among vaccine recipients as correlates of risk of HIV acquisition and as correlates of vaccine efficacy between Month 7 and Month 24 generated the hypothesis that the vaccine regimen conferred protection to a small subgroup of vaccine recipients, defined by having the highest Month 7 IgG3 HIV Envelope (Env) V1V2 breadth scores.^12^ The IgG3 V1V2 breadth score was a weighted average of eight V1V2 antigen-specific (including clades AE, B, and C) IgG3 binding antibody readouts. VE rose with increasing Month 7 IgG3 V1V2 breadth score, with VE 11.8% (95% CI, −17.9% to 34.0%) at all IgG3 V1V2 breadth score values below 667 weighted geometric mean net mean fluorescence intensity (MFI); VE of 62.6% (95% CI, −17.9% to 89.6%) just above 667 net MFI, and 80.9% (95% CI, −17.9% to 99.5%) at 1471 net MFI.^12^ These results supported IgG3 V1V2 binding antibody levels as a potential correlate of protection. Less than 10% of vaccine recipients attained an IgG3 V1V2 breadth score higher than 667 MFI, which potentially explains the lack of overall efficacy in the trial.

The immune correlates analysis^12^ was “host immune response-focused” as it assessed immune markers as correlates of HIV-1 acquisition without accounting for features of acquired viruses. For genetically diverse pathogens such as HIV, a complementary “pathogen-focused” approach, genotypic sieve analysis,^13^ can yield further insights towards mechanisms of protection. Genotypic sieve analysis^14^, ^15^, ^16^, ^17^, ^18^, ^19^, ^20^, ^21^, ^22^ uses pathogen sequence data to assess whether and how vaccine efficacy depends on pathogen sequence features, such as the presence of a certain amino acid (vs. not that amino acid) at a given sequence position. Sieve analyses can generate hypotheses about specific pathogen features marking immune vulnerability. For example, the RV144 trial sieve results suggested that protective antibodies may target the V2 region.^15^^,^^23^ The present work addresses a secondary objective of the HVTN 705 trial: “To evaluate and compare genomic sequences of viral isolates from HIV-1–infected vaccine and placebo recipients and use sieve analysis methods to assess whether VE differs by genotypic or phenotypic characteristics of exposing HIVs and whether there is evidence of vaccine-induced immune pressure on the viral sequences”.

## Methods

### HVTN 705 trial

Information on participant sex and gender was collected via participant self-report. Participants could choose from pre-determined responses (male, female) on sex and from pre-determined responses on gender. Eligible participants were centrally randomly assigned (1:1) in stratified permuted blocks, through a sequence obtained through computer-generated random numbers, to receive vaccine or placebo. All syringes were covered with an overlay to preserve blinding. Additional details on study design; study period; vaccine regimen; blinding; HIV diagnostic procedures; full inclusion and exclusion criteria; secondary objectives and endpoints; participant baseline demographic, risk, and clinical characteristics; and trial oversight are provided in Gray et al.^11^

### HIV-1 viral load and env sequencing

Treatment group comparisons (vaccine vs. placebo) of mean log10 viral load in the first RNA-positive sample and at 2–6 weeks after first RNA detection were performed using a two-sided Wald confidence interval and test from a linear regression model adjusted for baseline age, body mass index (BMI), HIV risk score, and enrolment in South Africa vs. a different country.

To assess a treatment difference in the post-first-detection viral load decline, we performed a post-hoc exploratory longitudinal analysis of log10 viral load measurements from first RNA detection through ART initiation. We fit a linear mixed-effects model including the vaccine group indicator, time in days since the first RNA-positive sample, the treatment-by-time interaction, the indicator of enrolment in South Africa, and a participant-level random intercept. The interaction coefficient estimate and a percentile bootstrap 95% CI were used to assess the treatment difference in the mean daily change in log10 viral load after first RNA detection.

To evaluate treatment differences in mean nucleotide and amino acid (AA) sequencing depth in the first RNA-positive sample, the same covariate-adjusted linear regression framework was applied as for the cross-sectional viral load analyses, with inference based on percentile bootstrap confidence intervals and permutation tests.

An HIV genomic region spanning *rev*, *vpu*, *env*, and partial *nef* was sequenced in the earliest HIV-1 RNA PCR-positive plasma sample from each primary endpoint case. We employed the PacBio single molecule real-time unique molecular identifier (SMRT-UMI) sequencing methodology,^24^^,^^25^ which allowed accurate single-genome sequencing at great depth by using universal molecular identifiers and provided an accurate representation of emerging *in vivo* virus lineage populations by removing PCR misincorporation errors and nearly eliminating recombination artefacts. Nucleotide sequences were aligned separately within each primary endpoint, truncated to the *env* gene, and translated to Env AA sequences. Within each participant, AA sequences were downselected after applying a functional filter with the following exclusion criteria: (1) a premature stop codon occurring more than 5% of the *env* length from the 3′ end, (2) gp160 length <80% of the median sequence length, with the median calculated within each participant's sequences, (3) missing the 5′ end including the ATG start codon, and (4) absence of the ATG start codon. The per-participant sequence alignments were created with MUSCLE^26^ and then visually inspected and manually adjusted by three different reviewers as previously described.^27^

### Virus lineage multiplicity identification

For each participant's nucleotide sequence alignment, we calculated the pairwise sequence Hamming distance (HD) distribution and counted the number of peaks to distinguish unimodal distributions from multimodal ones. This was motivated by the observation that within-lineage inter-sequence HD distributions are unimodal, whereas distributions comprising multiple lineages are generally multimodal, depending on the level of recombinant sequences. This assumption is usually met when sequences are sampled within the first few weeks from HIV-1 acquisition.^28^^,^^29^

For the multimodal distributions, we recalculated the inter-sequence HD distribution a second time to control for the effect of G-to-A hypermutation patterns associated with APOBEC3G/F signature, this time excluding hypermutated sequences (defined as yielding a P-value ≤0.1 from the LANL tool Hypermut^30^^,^^31^), ignoring all the positions in the alignment where a G was embedded within an APOBEC3G/F motif. Enrichment for G-to-A hypermutation patterns with APOBEC3G/F signatures^29^ happens at a higher rate than the expected *env* mutation rate and thus yields multimodal HD distributions even in the case of a single early virus lineage. If the number of peaks remained multimodal, these changes in the alignment were visualised via highlighter plot^32^ to confirm that all multi-modal distributions were indeed caused by distinct viral lineages rather than early immune escape as described above.^33^^,^^34^ Frequencies of multiple lineage primary endpoints were compared between treatment groups using Fisher's exact test.

### Intra-host env sequence diversity and lineage divergence

The genetic diversity analysis, defined as the pairwise genetic diversity across all intra-host sequence pairs, was conducted on each intra-host *env* nucleotide sequence alignment, after excluding hypermutated sequences. The per-nucleotide pairwise HD was defined as the number of nucleotide mutations comparing two intra-host sequences divided by the sequence length and calculated for all intra-host sequence pairs. Shortly after entering a new host, HIV starts accumulating mutations at an average rate of 2.16 × 10^−5^ per site per generation cycle.^28^^,^^33^^,^^35^ Therefore, *env* sequences observed at a later timepoint should have a higher mean per-nucleotide pairwise HD. To adjust for the temporal effect, we also calculated the per-nucleotide pairwise HD rate per day, defined as the HD divided by the number of days from the last HIV-1 RNA-negative sample collection date to the collection date of the sequenced sample. Gap regions within the alignment introduced by insertions or deletions were ignored. If an individual had a total of 3 or fewer identical or nearly identical *env* sequences, we could not rule out the presence of additional lineages, hence no call on the number of early virus lineages was made for the individual, and they were excluded from the intra-host diversity analysis. The LANL tool RAPR^36^^,^^37^ was used to identify recombinants.

The lineage divergence analysis, defined as the per-nucleotide genetic distance between co-evolving genetically distinct virus lineages, was conducted on all primary endpoints classified as multiple lineage endpoints. We first identified the most common virus within each distinct lineage and, if more than two lineages were present, we considered the two most divergent ones for calculating the per-nucleotide HD between them. The intra-host mean per-nucleotide pairwise HD distributions and the intra-host inter-lineage per-nucleotide HD distributions of multi-lineage alignments were compared between the placebo and vaccine group using the two-sided permutation test from median quantile regression models adjusted for age, BMI, baseline HIV risk score, and enrolment in South Africa vs. a different country.

### Representative sequence selection for single-sequence sieve analyses

Most statistical sieve analysis methods use a single Env AA sequence representative of a primary endpoint case. For each primary endpoint, we selected an observed sequence with the shortest HD to the consensus sequence for that participant. The sequence with the minimum distance is referred to as the ‘mindist’ sequence—it is closest to a “centre of tree”^38^ sequence and optimised to best represent all observed sequences within each primary endpoint. If multiple unique observed sequences had the same minimum distance to the consensus sequence, the most prevalent sequence was selected. If this criterion still did not identify a unique sequence, one candidate sequence was selected at random. The representative mindist AA sequences for all primary endpoints were then aligned together, following the same alignment review process as used for the per-participant nucleotide sequence alignments, and this alignment was used for deriving sequence features for single-sequence sieve analyses. Subtype classification of mindist Env sequences was performed using the LANL tool Recombinant Identification Program (RIP).^39^^,^^40^

Complementary to single-sequence sieve analyses, we characterised the intra-host diversity and lineage divergence of all *env* nucleotide sequences measured from each primary endpoint case, as well as the intra-host variability among values, computed for the multiple measured Env AA sequences, of features analysed in tier 1 as described below. We also made inferences about differential VE for tier 1 and tier 2 binary features accounting for all measured Env AA sequences from each case.

### HIV-1 Env AA sequence features for sieve analysis

All Env AA sequence features interrogated for sieve effects were prespecified before treatment unblinding unless otherwise noted as a post-hoc exploratory analysis. Features were divided into two tiers: 1) features hypothesised to potentially impact vaccine efficacy based on immunology/structural biology knowledge, theory, and experiments; and (2) all other features with sufficient AA variability within Env (HXB2 positions 1–856) to allow potential discovery of additional sieve effects. The decision was made to evaluate sufficiently variable Env positions broadly, including regions not represented in the vaccine sequences [Ad26 vaccine Env sequences: HXB2 positions 1–681 (Mos1) and 1–712 (Mos2S); gp140 C97ZA vaccine sequence: HXB2 positions 1–683] because sieve analysis can in principle identify sequence features associated with differential VE even when the underlying positions are not themselves contained in the immunogens. Potential drivers of such “indirect” signals may include distant Env sites that are related functionally or structurally, compensatory mutations that confer evolutionary advantage, and/or immune pressure on a different Env region resulting in post-acquisition adaptation.

Briefly, selection of sequence features in tier 1 was driven by previous findings and hypotheses summarised in Table 1. The listed hypotheses gave rise to the following seven types of tier 1 features specified for sieve analysis: 1) presence vs. absence of a particular observed residue at an AA position in the union of sets a)–f) in Table 1; 2) presence vs. absence of a potential N-linked glycosylation site (PNGS) motif starting at an AA position in set g) in Table 1; 3) Hamming distance, with positions weighted by physicochemical (PC) properties of AAs, from each of the three Env vaccine insert sequences (Mos1, Mos2S, C97ZA), separately in each sequence region defined in a), b), c), e), f1), f2) in Table 1; 4) PC-weighted Hamming distance from the binding antibody assay's gp70-001428.2.42 V1V2 antigen sequence in position set b) in Table 1; 5) a V1V2 region characteristic listed in h) in Table 1; 6) cysteine count in Env gp160; 7) single vs. multiple early virus lineages driven by hypothesis j) in Table 1. C97ZA was chosen for the primary reference sequence for these Env-focused sieve analyses because an Env sieve signal is plausibly more related to antibody recognition than to T-cell epitope coverage, and the C97ZA gp140 protein immunogen was designed specifically to boost humoural responses against a clade C Env (in contrast to the Ad26 mosaic vector inserts, which were designed to induce broad T-cell responses).

**Table 1:** Tier 1 Env AA sequence features prespecified for sieve analysis.

Finding(s)/Hypothesis and Reference(s)	Tier 1 AA Position Set/Sequence Feature
*Position-specific hypotheses/features: the analysed feature associated with each listed AA position is presence vs. absence of a specific observed AA residue in a)–f) and presence vs. absence of a PNGS in g)*	
a) Env AA positions 157–184 in the N-terminal portion of V2 associated with vaccine protection in the NHP challenge model^41^	Env AA positions 157–184
b) IgG3 V1V2 breadth score trended as an inverse correlate of HIV-1 acquisition risk, with the strongest signal for IgG3 concentration against the gp70-001428.2.42 V1V2 antigen, in the HVTN 705 immune correlates analysis^12^	15 AA positions in the non-hypervariable region of V1V2 with a residue mismatch between the strongest and at least 1 of the two weakest gp70-scaffolded V1V2 correlate antigen sequences, identified as potential sites of vulnerability driving the IgG3 V1V2 correlates result
c) High ADCC in combination with low plasma anti-HIV-1 Env IgA antibodies correlated inversely with HIV-1 acquisition risk in the RV144 trial^42^^,^^43^	21 AA positions delineating the ADCC epitopes on gp120 that are targeted by vaccine-induced ADCC-mediating antibodies (including linear epitopes in the C1 region)
d) AA positions 169 and 181 associated with differential vaccine efficacy in the RV144 trial^15^	Env AA positions 169 and 181
e) Env AA positions identified as having increased frequency of residues associated with resistance to bnAbs known to be most potent against subtype C viruses (e.g., V3 bnAbs 10-1074 and PGT121; CD4bs bnAbs 3BNC117 and N6; and MPER bnAb 10E8)^44^	11 Env AA positions with increased frequency of residues associated with resistance to bnAbs listed on the left
f) Signature regions identified that significantly impacted VE in the HVTN 505 trial's sieve analysis^18^	f1) 54 AA positions covering four k-mer regions overlapping the CD4bs in one of the signature regions described on the left^a^
	f2) 93 AA positions comprising CD4bs monoclonal antibody footprints in one of the signature regions described on the left^a^
g) AA positions associated with changes over time in presence/absence of glycans compared to historic Env sequences and associated with changes in bnAb resistance,^44^ plus AA positions of two glycan holes in the C97ZA vaccine insert sequence,^45^^,^^46^ raising the hypothesis that the vaccine could potentially elicit responses targeting these holes	Env AA position 130 for the CD4bs bnAb class; positions 156, 160 for the V2 bnAb class; positions 332, 392 for the V3 bnAb class; and positions 234, 392 of the glycan holes in the C97ZA vaccine insert
*Position-independent hypotheses/features*	
h) V1V2 region characteristics associated with a fitness advantage during transmission or early post-acquisition or with increased bnAb resistance^44^^,^^47^	Length of V1V2 and number of PNGS in V1V2 (each of which correlated with V3 bnAb resistance), and electrochemical charge in V2 (which correlated with V2 bnAb resistance)
i) Env gp160 cysteine count associated with a sieve effect signal in the transmembrane domain of Env in the HVTN 505 sieve analysis^18^	Cysteine count in Env gp160
j) The multiplicity of infection, even in the absence of vaccine efficacy against the primary endpoint, if found to be differential between vaccine and placebo groups, is an indication of partial vaccine efficacy against specific strains.	Single vs. multiple early virus lineages

AA, amino acid; ADCC, antibody-dependent cellular cytotoxicity; bnAb, broadly neutralising antibody; CD4bs, CD4 binding site; Env, HIV-1 envelope; NHP, nonhuman primate; PNG, potential N-linked glycosylation.

a Analysed only in aggregate as a physico-chemical Hamming distance from a vaccine insert sequence.

Additionally, we analysed three types of sequence features in tier 2: 1) presence vs. absence of a particular observed residue at an AA position in the complement of the tier 1 position set within Env; 2) PC-weighted Hamming distance from each Env vaccine insert sequence with C97ZA selected as the primary reference sequence, separately in the full gp120, the vaccine insert-covered N-terminal portion of gp41, and the V5 loop; 3) length and number of PNGS motifs in V5, which have been correlated with CD4bs broadly neutralising antibody (bnAb) resistance. AA positions were numbered using HXB2 coordinates throughout.

### HIV-1 Env predicted structural features for single-sequence sieve analysis

In addition to Env AA sequence-derived features, we prespecified tier 2 features derived from AlphaFold3 (AF3)-predicted Env trimer structures corresponding to the same observed mindist and vaccine-insert (C97ZA) Env sequences. Structure prediction was performed using the gp120 (positions 31–511) and gp41 (positions 512–665) regions, which yield reliable multimeric structures in AlphaFold^48^ and high-confidence trimeric structures in AF3. For each sequence, the top-scoring model among five AF3 predictions was selected for downstream analyses.

To quantify sieve effects arising from structural divergence between breakthrough viruses and vaccine sequences, we computed the template modelling (TM) score,^49^ a topological similarity metric for comparing each predicted primary endpoint structure to the predicted structure of each vaccine sequence, using a single protomer chain. The TM-score was chosen because it is length-independent, enabling structural comparisons of proteins of unequal length.

Because the gp140 region was relatively homogeneous among observed sequences—yielding minimal signal in full-protein TM-scores—we implemented a sliding-window approach to assess localised structural variation. Specifically, we calculated TM-scores of overlapping 15-mer windows across gp120, with a 4-position overlap between consecutive windows. We excluded windows that contained any hypervariable loop positions. Each 15-mer corresponded to 15 linear HXB2 alignment positions rather than 15 linear AAs in the structure to ensure that homologous structural regions were compared. A minimum threshold of six AAs per window was required to avoid spuriously large differences dominated by point mutations. Sensitivity analyses using higher thresholds produced comparable results but overall increased similarity values. Shorter windows tended to yield weaker, not stronger, signals, indicating that slightly longer N-mers provided more discriminative power. Therefore, the six-AA threshold was retained to avoid the removal of potentially significant windows based on length. Treatment group differences in TM-scores, standardised to the [0–1] interval, were evaluated using permutation tests based on linear regression models adjusted for baseline age, BMI, HIV risk score, and enrolment in South Africa vs. a different country.

### Vaccine efficacy as a function of an HIV-1 Env AA sequence feature in single-sequence sieve analysis

We defined AA sequence feature-specific vaccine efficacy (VE) as one minus the hazard ratio (vaccine/placebo) of the HIV-1 diagnosis primary endpoint characterised by a particular specified feature level of the mindist sequence. For binary features such as presence/absence of a residue at a given AA position, feature-specific VE was estimated using a competing-risks Cox proportional hazards model with vaccine vs. placebo treatment assignment as the sole covariate, including Wald 95% confidence intervals (CIs), and testing for differential VE across the two feature levels with a two-sided Wald test.^50^ For quantitative features with >4 distinct values such as a PC-weighted Hamming distance, the feature-specific VE was estimated using a continuous mark-specific proportional hazards model, with pointwise Wald 95% CIs, and a two-sided Wald test for varying VE across the distinct levels of the feature supported in the data.^51^ The competing-risks Cox models^50^^,^^51^ were stratified by enrolment in South Africa vs. in a different country. Throughout, a sieve effect refers to statistically significant evidence for variation in feature-specific VE across distinct levels of the feature under consideration (Fig. S1).

### Inter- and intra-host position-specific amino acid residue diversity

In a post-hoc analysis to assess whether the vaccine influenced inter-host viral diversity at a particular AA position, we calculated the Shannon entropy of AA residues at that position in the mindist sequences, separately for vaccine and placebo groups. These were compared with the position-specific Shannon entropy of a reference set of non-study Env AA sequences that were selected to be epidemiologically relevant to the HVTN 705 trial. The reference set was selected from the Los Alamos National Laboratory (LANL) HIV Sequence Database's 2022 premade alignment,^52^ using the following inclusion criteria: subtype C, sample collection date between 2017 and 2022, and geographic origin from one of the five countries that participated in the trial (Malawi, Mozambique, South Africa, Zambia and Zimbabwe). A total of 95 Env sequences met these criteria. However, phylogenetics analysis revealed that two sequences were from the same person. One of these two sequences was removed at random, resulting in a total of 94 reference sequences for this analysis. For each group, a nonparametric percentile bootstrap with 1000 replicates was used to obtain a 95% confidence interval (CI) for position-specific Shannon entropy. For the treatment comparison, a 95% CI for the difference in Shannon entropy was calculated by resampling participants separately within each treatment group.

To examine intra-host residue diversity at a particular AA position, separately for vaccine and placebo groups, the intra-host Shannon entropy of AA residues at that position was calculated for each primary endpoint, and the treatment-specific mean entropy was reported with t-distribution-based 95% CIs. The treatment difference in mean intra-host entropy was summarised with a two-sample unequal-variance t-distribution-based 95% CI.

### Prescreening of HIV-1 Env AA sequence features for single-sequence sieve analysis

To increase power to detect sieve effects, we screened all binary features for sufficient variability defined as ≥4 primary endpoint cases exhibiting each of the two levels of the feature. Conditioning on the primary endpoint split by treatment, the threshold of 4 cases was obtained as the minimum number of cases of each binary type required for Fisher's exact test to yield a P-value <0.05 (i.e., to detect a sieve effect) if all cases of one type were observed in the same treatment group. Binary features associated with AA positions in the hypervariable regions (Table S1) were excluded due to ambiguous alignability, and AA positions within those regions were excluded in calculations of PC-weighted Hamming distances. In contrast, hypervariable region characteristics such as a variable loop length included hypervariable positions as these features were, by definition, independent of the alignment. The number of PNGS motifs in the V5 loop had 4 unique values among primary endpoints and was dichotomised as ≤1 vs. ≥2.

### High-dimensional hypothesis testing for single-sequence feature differences between treatment groups

To assess whether a high-dimensional signal was present in the multivariate sequence feature space distinguishing vaccine from placebo endpoints, and to identify the subsets of features contributing most strongly to this signal, we applied the Direction-Projection-Permutation (DiProPerm) method.^53^ DiProPerm is a nonparametric test for differences between two high-dimensional distributions. The method projects data onto a one-dimensional discriminative direction—here, the mean difference (MD) direction—and computes a two-sided P-value using treatment-group balanced permutations to test the global null hypothesis that all features in the set have the same distribution in the two treatment groups.

We conducted DiProPerm analyses across 97 prespecified sequence feature subsets (Table S2). A set of control covariates—age, BMI, baseline HIV risk score, and enrolment in South Africa—was analysed independently as subset 1 and also included as adjustment variables in all other subsets. Feature groups composed of multiple subsets (e.g., subsets 2a–2e) were also analysed in aggregate (e.g., subset 2 combining all features from 2a–2e). This aggregation approach was applied to all multi-subset feature groups except for the structural feature group (13), in which distinct subgroups represented region-specific TM-score features.

We additionally evaluated hybrid subsets combining each of subsets 1–12 with the region-specific structural features (subsets 13o and 13p) to assess potential interactions between sequence- and structure-based features (e.g., ‘subset 2e hybrid’ combined subsets 2e, 13o, and 13p). Lastly, we performed a DiProPerm analysis using all non-hybrid feature subsets combined (excluding group-level aggregate subsets and representing structural features by subsets 13a, 13o, and 13p), resulting in a 2193-dimensional feature space (subset 14).

All variables were linearly rescaled to the [0, 1] interval prior to analysis to facilitate comparability of variable loadings in the MD direction. Each DiProPerm analysis used 1000 permutations to compute the global P-value. Because this exploratory analysis was intended to identify feature sets contributing to the overall treatment separation, global P-values were not adjusted for multiplicity.

### Multi-sequence sieve analysis

We also performed a post-hoc sieve analysis of binary features using the full deep-sequencing data measured in the earliest RNA-positive sample from each primary endpoint case. For each participant and binary feature, the observed within-host feature prevalence was calculated as the number of sampled sequences carrying the feature divided by the total number of sampled sequences. Because, at first detection, sequencing depth varied across participants and tended to be lower among vaccine recipients, the multi-sequence sieve analysis did not classify endpoint cases deterministically according to this observed feature prevalence. Instead, the method defined the viral features of interest in terms of the true feature prevalence in the underlying whole viral quasispecies in blood (a latent variable that is unobserved); specifically these features are the probabilities that the participant's quasispecies belonged to the threshold-defined category ≥1% vs. <1% prevalence, or ≥99% vs. <99% prevalence. Thus, endpoint cases with lower sequencing depth contributed with greater classification uncertainty, rather than being classified deterministically as ≥1% vs. <1%, or ≥99% vs. <99%, based solely on the sampled sequences.

We then estimated and compared vaccine efficacy against viral populations with ≥1% vs. <1% feature prevalence, and separately as having ≥99% vs. <99% feature prevalence (in the whole unobservable quasispecies in blood), using a method, available in a preprint, designed to use sample frequencies to estimate the viral features and to properly account for the errors in these estimates, which are especially acute for samples with low sequencing depth.^54^ This method uses a measurement-error version of a competing-risks Cox model adjusted for age, BMI, HIV risk score, and enrolment in South Africa vs. a different country, treating the viral population types (≥1% vs. <1%, and ≥99% vs. <99% feature prevalence) as competing risks. Testing for differential VE was performed using a two-sided Wald test with the covariance matrix estimated using nonparametric bootstrap. The 1% and 99% thresholds were selected because the median sequencing depth of 121 corresponded roughly to a limit of detection of variants with prevalence slightly less than 1% in the sampled sequences. Both thresholds were included because both the dominance and scarcity of a sequence feature were scientifically relevant.

### Prescreening of binary HIV-1 Env AA sequence features for multi-sequence sieve analysis

For binary features to be eligible for the analysis, we set two screening criteria, the first for sufficient inter-individual diversity and the second for sufficient intra-individual diversity. The first screen required ≥4 primary endpoints with <1% (or <99%) of the sequences with the feature, and ≥4 primary endpoints with ≥1% (or ≥99%) of the sequences with the feature. The second screen required that comparing the dichotomisation of primary endpoints based on the 1% (or 99%) threshold and the dichotomisation based on the mindist sequence feature value resulted in category switching in ≥10% of primary endpoints.

### Multiple testing correction

Given the number of hypothesis tests for variation in VE (i.e., sieve effect tests) reflecting the multitude of analysed sequence features, we implemented a prespecified multiplicity P-value adjustment approach. Multiplicity adjustment was performed separately for the single-sequence vs. multi-sequence sieve analyses, because these analyses addressed related but distinct scientific questions and used different feature constructions. Sequence features were divided into seven mutually exclusive multiplicity sets defined by feature and analysis types (Table S3). Sieve testing for PC-weighted Hamming distances from the Mos1 and Mos2S vaccine insert sequences and the gp70-001428.2.42 V1V2 antigen sequence was performed as a sensitivity analysis with no reporting of adjusted P-values. The test for differential VE by single vs. multiple early virus lineages was not adjusted for testing multiplicity. Within each non-empty multiplicity set, we calculated adjusted P-values controlling the familywise error rate (FWER) and the false discovery rate (FDR) (Q-values) using a permutation-based method^55^^,^^56^ implemented in Fong et al.^57^ No additional multiplicity adjustment was applied across the seven multiplicity sets; therefore, adjusted P-values should be interpreted as controlling multiple testing within each prespecified set. We defined FWER statistical significance as an FWER-adjusted P-value ≤0.05 and FDR statistical significance as a Q-value ≤0.20 and an unadjusted P-value ≤0.05 within each multiplicity set.

### Ethics

The HVTN 705 trial adhered to the principles of the 2024 Declaration of Helsinki and Good Clinical Practice guidelines. The protocol, protocol amendments, and other relevant documents were approved by the following institutional review boards (IRBs), ethics committees, and applicable regulatory entities: Malawi—Malawi National Health Sciences Research Committee, University of North Carolina IRB (due to site academic affiliation); Mozambique—Mozambique INS Health Bioethics Institutional Committee; South Africa: University of Witwatersrand Human Research Ethics Committee, University of Cape Town Human Research Ethics Committee, South Africa Medical Research Council Ethics Committee, University of KwaZulu-Natal Biomedical Research Ethics Committee, University of Pretoria Research Ethics Committee, Sefako Makgatho University Research Committee; Zambia—University of Zambia Biomedical Research Ethics Committee, University of Alabama IRB (due to site academic affiliation), Emory University IRB (due to site academic affiliation); and Zimbabwe—University of Zimbabwe Joint Research Ethics Committee. Sequencing work conducted at the University of Cape Town was approved by the University of Cape Town Human Research Ethics Committee (UCT HREC REF 234/2021).

All participants provided written informed consent. The HVTN 705 trial is registered with ClinicalTrials.gov, NCT03060629 (https://clinicaltrials.gov/study/NCT03060629; study registration first posted February 23, 2017). The HVTN 705 study protocol and Statistical Analysis Plan are available in the Supplementary Appendix (pp. 18–163 and pp. 164–220, respectively) of Gray et al.^11^ An independent data and safety monitoring board periodically reviewed the safety and efficacy data. Interim monitoring for potential harm, non-efficacy, and high efficacy is detailed in section 10 of the SAP in Gray et al.^11^

The HVTN 705 trial exhibited principles of equity and inclusivity in its conduct by supporting full HIV-1 prevention services before and during the study [oral pre-exposure prophylaxis (tenofovir disoproxil fumarate–emtricitabine) was offered either directly or at an affiliated facility proximal to the study site], by engaging community stakeholders, and by recruiting participants from a group at high risk that is under-represented in HIV-1 research. Community Advisory Boards are required by DAIDS and supported at all HVTN research sites to ensure community input, in accordance with Good Participatory Practices (GPP) and all local and national guidelines. Clinical research sites participating in HVTN 705 were selected from accredited HIV Vaccine Trials Network clinical research sites based on epidemiologic suitability, regulatory and operational readiness, and the capacity to conduct a large phase 2b HIV vaccine efficacy trial. In addition to meeting HVTN-wide requirements, sites were required to demonstrate access to a population of young women at risk for HIV acquisition, the ability to deliver the protocol-defined standard of HIV prevention and care, established community engagement infrastructure, and successful completion of protocol-specific site initiation procedures prior to enrolment.

### Statistics

Details on the statistical methods used for the different analyses, inclusion/exclusion criteria for the different analyses, the statistical tests used for comparisons, and the approach to multiplicity adjustment are provided in the sections above. A methodology flowchart is also provided as Fig. S1 for reference. All statistical analyses were prespecified before treatment unblinding unless otherwise noted as a post-hoc exploratory analysis. All reported P-values are from two-sided tests. The Statistical Analysis Plan for Genotypic Sieve Analysis v4.0 is provided as a Supplementary File.

Formal sample size calculation was not done for this sieve analysis. Sample size was determined by the number of participants in the PP cohort who acquired an HIV-1 primary endpoint and had Env sequence data available from the earliest timepoint. The analysis was considered feasible if at least 40 such primary endpoints were available overall, provided that endpoint counts were not extremely imbalanced by treatment group. This threshold was deemed a suitable number to provide enough data to have reasonable power for the detection of meaningful sieve effect sizes, based on previous sieve analyses.^14^, ^15^, ^16^, ^17^, ^18^, ^19^, ^20^, ^21^, ^22^ The present study substantially exceeded this threshold, with reasonably balanced endpoint counts by treatment group.

### Role of Funders

This study was co-funded through a public-private partnership that included the National Institute of Allergy and Infectious Diseases (NIAID) and its Division of AIDS (DAIDS), both of the National Institutes of Health (NIH); the HIV Vaccine Trials Network (HVTN) (Fred Hutchinson Cancer Center); and the US Army Medical Materiel Development Activity. The trial was co-designed by the NIAID-supported HVTN and conducted through the HVTN. All co-funding parties participated in trial design and in the collection, analysis, and interpretation of data. This manuscript was reviewed by DAIDS, the HVTN, and Johnson & Johnson. The authors were not precluded from accessing data in the study, and they accept responsibility to submit for publication.

For the HVTN 705/HPX2008 efficacy trial (Gray et al.^11^), the HVTN and Johnson & Johnson, in collaboration with the Gates Foundation (formerly the Bill & Melinda Gates Foundation) and Ragon Institute, had roles in the study design. The HVTN, Johnson & Johnson, and the Ragon Institute had roles in the collection, analysis, and interpretation of data in Gray et al.; the writing of the Gray et al. paper; and the decision to submit the Gray et al. paper for publication.

## Results

### HIV-1 viral load and env sequencing depth

**Fig. 1:**
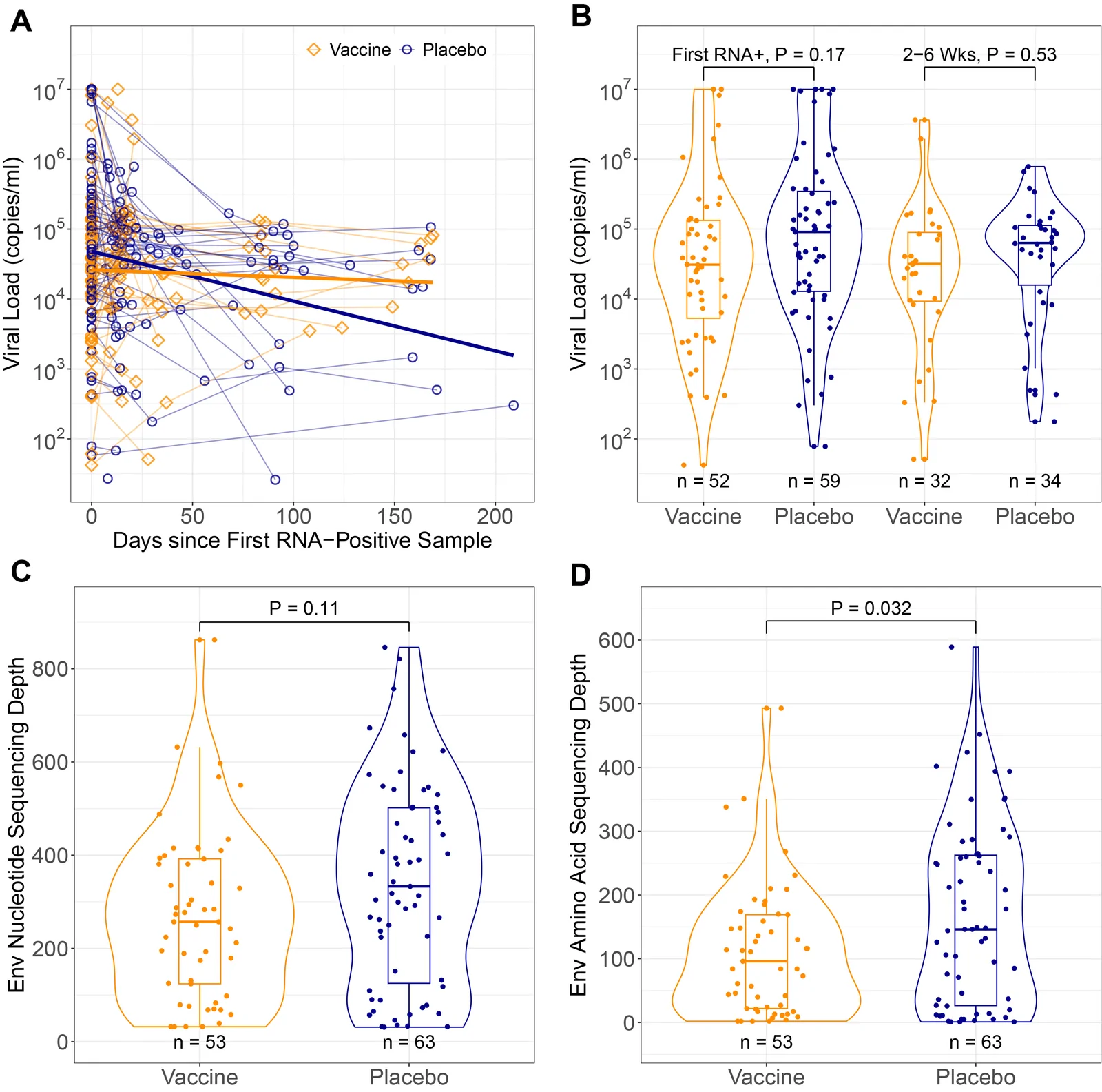
Analyses of HIV-1 viral load (copies/ml) in primary endpoints and of sequencing depth. (A) Longitudinal trajectories of HIV-1 viral load (copies/ml) in primary endpoints [n = 54 vaccine, n = 64 placebo (1 missing placebo viral load)], shown by treatment group, starting on the day of the first RNA-positive sample and extending through the last pre-ART sample. The thicker lines show estimated treatment-specific mean rates of decline from a longitudinal mixed effects model. (B) HIV-1 viral loads in primary endpoints, shown by treatment group, in the first RNA-positive sample [left side, n = 52 vaccine, n = 59 placebo; P = 0.17 (two-sided linear regression test—t-test for log10 viral load)] and individual-level geometric mean (GM) of viral loads 2–6 weeks later while pre-ART initiation [right side, n = 32 vaccine, n = 34 placebo; P = 0.53 (two-sided linear regression test—t-test for log10 viral load)]. (C) Env sequencing depth for nucleotide sequences from primary endpoints, shown by treatment group [n = 53 vaccine, n = 63 placebo; P = 0.11 (two-sided linear regression test—permutation test)]. (D) Env sequencing depth for amino acid sequences from primary endpoints after application of the functional filter [n = 53 vaccine, n = 63 placebo; P = 0.032 (two-sided linear regression test—permutation test)]. All regression models were adjusted for age, BMI, baseline risk score, and enrolment in South Africa vs. a different country. Violin plots in B–D contain interior box plots with upper and lower horizontal edges at the 25th and 75th percentiles and middle line at the 50th percentile, and vertical bars the distance from the 25th (or 75th) percentile and the minimum (or maximum) level within the 25th (or 75th) percentile minus (or plus) 1.5 times the interquartile range. Each side shows a rotated probability density (estimated by a kernel density estimator with a default Gaussian kernel) of the data.

Throughout we analysed the PP cohort of 2188 participants (1080 received vaccine; 1108 received placebo) using the same definition as in Gray et al.^11^ A total of 119 PP participants (54 vaccine; 65 placebo) acquired an HIV-1 primary endpoint. Longitudinal HIV-1 viral loads from the first RNA-positive sample through the last pre-ART sample are shown by treatment group in Fig. 1A. In the earliest RNA-positive sample, viral load trended lower in vaccine than placebo recipients [geometric mean (GM) = 33,624 vs. 67,854 copies/ml; GM ratio = 0.50 (95% CI, 0.18–1.37); P = 0.17 (linear regression Wald test)] (Fig. 1B). This trend diminished by 2–6 weeks post-RNA detection and pre-ART initiation [GM = 24,059 vs. 34,259 copies/ml for vaccine vs. placebo; GM ratio = 0.70 (95% CI, 0.23–2.17); P = 0.53 (linear regression Wald test)] (Fig. 1B). In a post-hoc longitudinal analysis, the pre-ART GM viral load after first RNA detection declined faster in placebo than vaccine recipients [treatment–time interaction coefficient = 0.006 log10 copies/ml per day (95% CI, 0.002–0.010)] (Fig. 1A).

Env sequence data from the earliest RNA-positive sample were available for 116 of the 119 primary endpoints (53 vaccine; 63 placebo); data were missing for one case with undetectable viral load and two with failed PCR amplification associated with very low viral loads (Table 2). Consistent with the viral load findings, PacBio sequencing depth at first RNA detection was lower in the vaccine vs. placebo group [mean = 114 vs. 166 amino acid (AA) sequences; mean difference = −52 (95% CI, −96 to −8); P = 0.032 (linear regression permutation test)], an association more pronounced for translated Env AA sequences after application of a functional filter (see Methods) (Fig. 1D) than for the original nucleotide sequences which included defective viral sequences (Fig. 1C).

**Table 2:** HIV-1 acquisition diagnosis primary endpoints and available HIV-1 Env sequence data by treatment assignment.

	Vaccine	Placebo	Total
No. of primary endpoints^a^	54	65	119
No. Of primary endpoints with available first RNA+ sample Env sequence data	53	63	116
No. of primary endpoints as single-lineage infections^b^	33	42	75
No. of Env nucleotide sequences per primary endpoint: Mean (Range)	239.0 (2, 832)	308.7 (1, 816)	276.8 (1, 832)
No. of Env amino acid sequences per primary endpoint: Mean (Range)^c^	110.8 (2, 493)	168.3 (1, 589)	142.0 (1, 589)

a HIV-1 diagnosis per the trial's testing algorithm in the per-protocol cohort between months 7 and 24.

b Unable to determine lineage information for 8 primary endpoints (4 vaccine; 4 placebo) with available first RNA+ sample Env sequence data due to low sequence counts.

c After application of the amino acid sequence functional filter.

### Env sequence data

A single Env AA sequence with the shortest Hamming distance (HD) to the consensus sequence for that participant (henceforth referred to as the ‘mindist’ sequence) was selected for single-sequence sieve analyses from each of the 116 primary endpoints (see Methods). Random selection among tied candidate sequences was required for only two primary endpoints. For one endpoint, two tied sequences were each observed once among five total sequences, both with HD = 2, and none of their consensus-mismatched Env AA positions was included in the preselected hypothesis-driven position set in Table 3. For the other endpoint, four tied sequences were each observed twice among 38 total sequences, all with HD = 2; among their consensus-mismatched positions, only position 281 was listed in Table 3. All mindist Env sequences were subtype C, except a single isolate from South Africa that was classified as a C/D recombinant.

**Table 3:** Tier 1 hypothesis-driven Env amino acid positions and screened-in residue presence/absence features for sieve analysis. Amino acid positions are numbered using HXB2 coordinates.

**65 *Env* amino acid positions** 51–61, 69–78, 130, 155, 157–184, 186, 200, 230, 234, 279, 280–282, 350, 364, 429, 432, 456, 471	
**Classification of positions by hypothesis**^a^	
V2-associated protection in NHP challenge model	157–184
IgG3 V1V2 correlate in HVTN 705	130, 155, 160, 161, 164–166, 170, 172, 173, 178, 179, 181, 186, 200
gp120 epitopes of ADCC correlate of risk in RV144	51–61, 69–78
Primary signature positions in RV144 sieve analysis	169, 181
Subtype C CD4bs-, V3-, and MPER-bnAb resistance-associated signatures	230, 279–282, 350, 364, 429, 432, 456, 471
**138 screened-in residue presence/absence features pertaining to the 65 *Env* amino acid positions** 51.P, 51.T, 57.D, 57.N, 59.K, 60.A, 60.G, 60.S, 61.H, 72.H, 78.D, 130.E, 130.H, 130.K, 130.N, 130.S, 130.T, 155.K,155.Q, 155.R, 155.T, 158.S, 160.K, 160.N, 161.A, 161.I, 161.M, 161.T, 161.V, 162.T, 163.S, 163.T, 164.E, 165.I, 165.L, 165.V, 166.K, 166.R, 166.T, 167.D, 167.N, 168.K, 168.R, 169.E, 169.I, 169.K, 169.Q, 169.R, 169.T, 170.K, 170.Q, 170.R, 171.K, 171.N, 171.Q, 171.R, 172.A, 172.E, 172.M, 172.T, 172.V, 173.H, 173.R, 173.S, 173.Y, 175.L, 177.Y, 178.K, 178.R, 179.L, 179.P, 179.S, 179.V, 181.I, 181.L, 181.T, 181.V, 182.E, 182.I, 182.T, 182.V, 183.A, 183.K, 183.P, 183.Q, 183.S, 184.I, 184.L, 186.D, 186.E, 186.G, 186.K, 186.N, 186.S, 186.T, 186.gap, 200.A, 200.T, 200.V, 230.D, 230.N, 234.N, 234.S, 279.D, 279.N, 280.N, 280.S, 281.A, 281.I, 281.T, 281.V, 282.K, 350.A, 350.E, 350.G, 350.K, 350.Q, 350.R, 364.A, 364.H, 364.P, 364.S, 429.E, 429.G, 429.K, 429.R, 432.K, 432.Q, 432.R, 456.R, 456.W, 471.A, 471.E, 471.G, 471.I, 471.Q, 471.T, 471.V	

ADCC, antibody-dependent cellular cytotoxicity; bnAb, broadly neutralising antibody; CD4bs, CD4 binding site; Env, HIV-1 envelope; NHP, nonhuman primate.

a Hypotheses described in Methods and Table 1.

Phylogenetic reconstruction of the 116 *env* mindist and 3 *env* vaccine sequences, rooted on the 2021 clade C consensus sequence, showed that the C/D recombinant was not farther away from the tree root than all other subtype C sequences. Of the two mosaic *env* vaccine sequences, the most central to subtype C was Mos2S, whereas Mos1 was the sequence farthest away from the consensus C tree root (Fig. S2). When classified by geographical sampling, *env* sequences from different countries were equally distributed around the tree (Fig. S3). Three subclades comprised exclusively sequences from South Africa; however, none corresponded uniquely to a single enrolment site or province (Fig. S3). Of note, a pair of mindist *env* sequences in the upper-left quadrant of the tree (from South Africa) appeared much closer to each other than any other pair, consistent with epidemiologically linked transmissions: their branch length was 0.019 compared with 0.18 for the next closest pair in the tree.

### Intra-host lineage multiplicity and sequence diversity

Among the 116 primary endpoints with *env* sequence data, the mean per-nucleotide pairwise *env* Hamming distance (HD) ranged between 0% and 4.4% (median, 0.2%). Of those, eight endpoints (four in the placebo group and four in the vaccine group) had too few (<3) sequences to reliably determine lineage multiplicity, and 108 (93%) had an identifiable number of early virus lineages, including 33 (31%) endpoints with two or more genetically distinct virus lineages [17 (29%) in the placebo group and 16 (33%) in the vaccine group]. We found no evidence for a difference in the frequency of multiple early virus lineages between the treatment groups.

We next compared, by treatment, genetic divergence between distinct intra-host lineages among the 33 confirmed multiple-lineage alignments. For each, we calculated the per-nucleotide HD between the most prevalent sequences of two distinct lineages (or the two most divergent if more than two were present). Intra-host lineage divergence ranged from 0.3%–9.4% (median, 1.6%), with no evidence of difference between the treatment groups [P = 0.71 (quantile regression permutation test)].

As expected, within both treatment groups, multiple-lineage alignments showed higher mean per-nucleotide pairwise HD than single-lineage alignments (Fig. S4). This finding reflects the presence of genetically distinct early virus lineages. Among multiple-lineage alignments, the median of the intra-host mean per-nucleotide pairwise HD was higher in the vaccine vs. placebo groups [0.7% vs. 0.4%, respectively; P = 0.023 (quantile regression permutation test)]. In contrast, the median of the HD in single-lineage alignments trended lower in the vaccine vs. placebo group [0.08% vs. 0.14%; P = 0.10 (quantile regression permutation test)]. We next adjusted for time since the last HIV-1 RNA-negative sample, given that viral diversity increases over time. After this adjustment, the median of the mean per-nucleotide pairwise HD rate (per day) was lower in the vaccine vs. placebo group in single-lineage alignments [P = 0.0085 (quantile regression permutation test)], whereas no difference in this rate was seen between the two groups in multiple-lineage alignments [P = 0.94 (quantile regression permutation test)] (Fig. S4).

We detected recombinant *env* sequences in 24 (73%) multiple-lineage *env* alignments, equally split between the treatment groups. Most recombinants were unique sequences, but for at least six endpoints, recombinant sequences formed one or more novel lineages.

### Trend of higher VE against viruses with C97ZA insert-matched residue at Env position 364

**Fig. 2:**
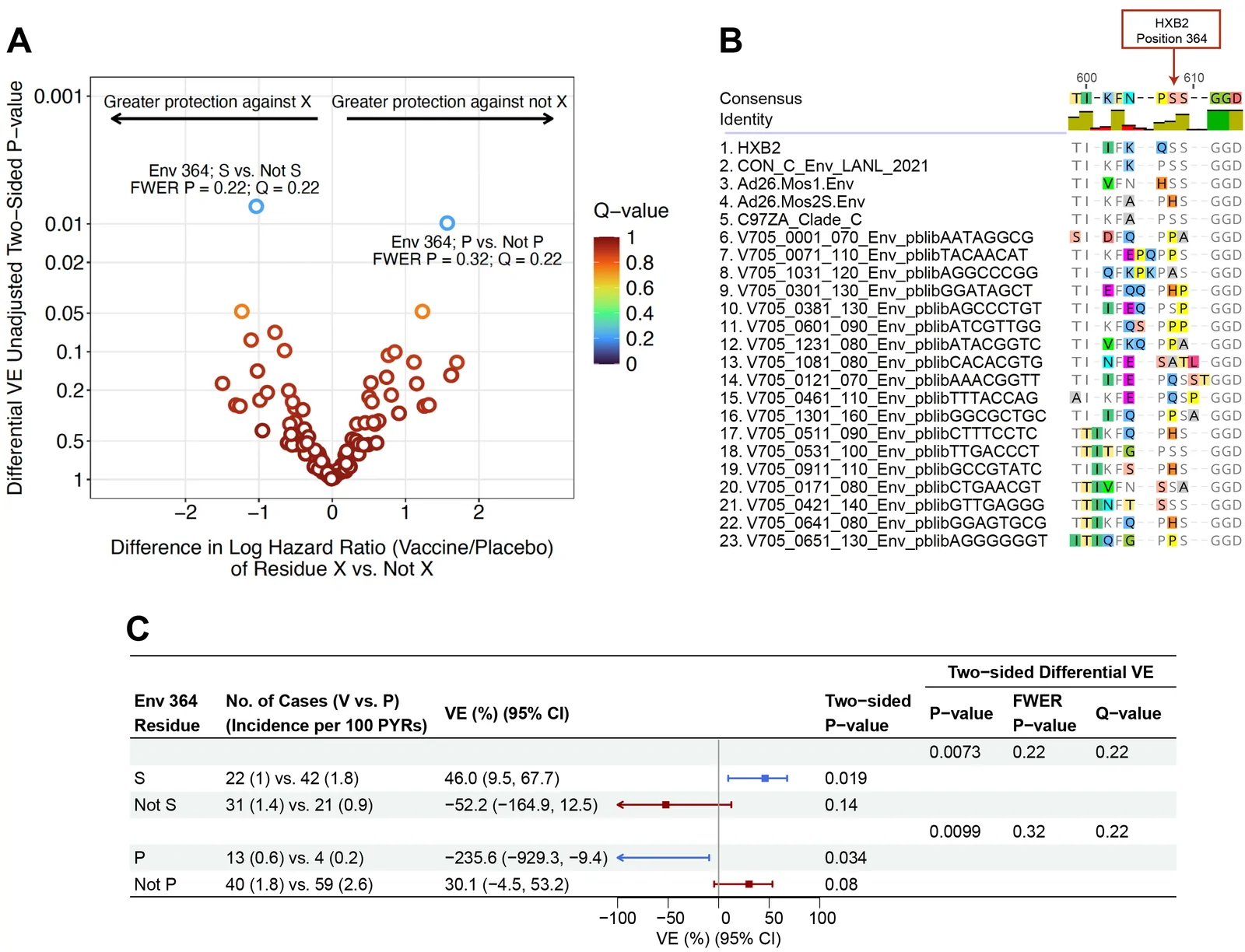
Sieve analysis of Env HXB2 position 364. (A) Difference in natural log hazard ratio (vaccine/placebo) of the primary endpoint (n = 53 vaccine, n = 63 placebo) (residue X-specific HIV diagnosis minus residue not X specific HIV diagnosis) vs. unadjusted two-sided P-value that tests for differential VE (Lunn-McNeil test^50^), calculated for all screened-in amino acid residue presence/absence features (n = 138). The colour reflects the Q-value. (B) Sections of inter-participant single representative sequence (mindist) alignments of Env amino acid sequences that encompass amino acid position 364. Reference sequences (n = 5) plus the participant mindist sequences with length variation in this region (n = 18) are shown. HXB2 amino acid position 364 corresponds to position 608 in this alignment (designated with the red arrow). (C) Vaccine efficacy (VE) against viruses with presence vs. with absence of a particular screened-in residue at Env position 364. The “Two-sided P-values” column shows Wald test P-values. Two-sided differential VE P-values were P = 0.0073 (S vs. not S, Lunn-McNeil test^50^) and P = 0.0099 (P vs. not P, Lunn-McNeil test^50^). Filled squares designate VE point estimates, and bars extend through the 95% confidence intervals (CIs).

We preselected a set of 65 Env gp120 AA positions based on previously generated hypotheses that vaccine protection against HIV-1 acquisition could vary with mutations at these positions (Table 3). Scanning the position set, we screened in 138 binary features each indicating a particular observed residue (a vaccine-matched residue or a vaccine-independent residue) in the mindist sequence. Screening eligibility required ≥4 primary endpoints representing each condition of the presence and absence of the residue. We found borderline-significant evidence of differential VE against HIV-1 genotypes defined by present vs. absent residue for two such features, Env S364 vs. not-S364 and P364 vs. not-P364 (Fig. 2A). Both features pertain to position 364, located in the CD4 binding loop (Fig. 2B). VE was 46% (95% CI, 10–68) against the C97ZA insert-matched residue S vs. −52% (95% CI, −165 to 13) against the C97ZA insert-mismatched residues P, H, A, or Q [unadjusted P = 0.0073 for differential VE (Wald test), FWER-adjusted P = 0.22, Q-value = 0.22] (Fig. 2C). Additionally, VE was −236% (95% CI, −929 to −9) against residue P vs. 30% (95% CI, −5 to 53) against residues S, H, A, or Q [unadjusted P = 0.0099 for differential VE (Wald test), FWER P = 0.32, Q = 0.22] (Fig. 2C).

Seven of the 116 sequences had a single AA insertion downstream from position 365, and two others had a two-AA insertion at this position. These insertions caused an ambiguity in the alignment, as they could have been aligned before or after positions 362–364. To confirm that the VE association found at position 364 was not due to this ambiguity, we conducted a sensitivity analysis using an alternative alignment (Fig. S5). This yielded 45% VE (95% CI, 7–67) against S364 vs. −45% VE (95% CI, −151 to 16) against viruses with other residues [unadjusted P = 0.012 for differential VE (Wald test)]. For both the original and alternative alignments, the residue at position 364 in the Ad26.Mos1.Env sequence was S [matching the clade C gp140 (C97ZA) protein sequence] and H in the Ad26.Mos2S.Env sequence. The difference in VE against H vs. not H had unadjusted P > 0.50 (Wald test). Additionally, because mutations at position 364 can cause a loss or gain of glycans at upstream positions, we assessed PNGS motifs starting at positions 362 and 363 but found no evidence of differential VE for the presence vs. absence of these motifs.

Given the borderline sieve effect at position 364, we conducted a post-hoc analysis of Shannon entropy^58^ to assess whether the vaccine influenced viral diversity at this site. The Shannon entropy among placebo recipient mindist sequences was 1.48 (95% CI, 1.08–1.74), comparable to that of non-study reference sequences [1.31 (95% CI, 0.99–1.53)]. Vaccine recipient mindist sequences showed an elevated entropy of 1.96 (95% CI, 1.67–2.10), yielding a difference (placebo minus vaccine) of −0.48 (95% CI, −0.87 to −0.079).

In a complementary scan of the remaining Env region, we evaluated all 796 Env positions not included in the hypothesis-driven position set, which yielded 1118 screened-in features of mindist sequences as defined above (Table S4). None of them exhibited evidence for differential VE by presence vs. absence of a particular residue, with all Q-values ≥0.72 (Fig. S6).

### No evidence of differential VE by presence vs. absence of a broadly neutralising antibody resistance-associated PNGS

We further preidentified Env positions of six signature glycans associated with increased bnAb resistance, two of which represent missing glycans, i.e., glycan holes, in the C97ZA insert sequence (Fig. S7). All six features indicating the presence of a PNGS motif in the mindist sequence met the inclusion eligibility criterion stated above and were screened in. We found no evidence of differential VE by PNGS presence vs. absence in this position set, with all Q-values ≥0.93 (Fig. S7).

### Significant decline in VE with increasing weighted Hamming distance from C97ZA insert

**Fig. 3:**
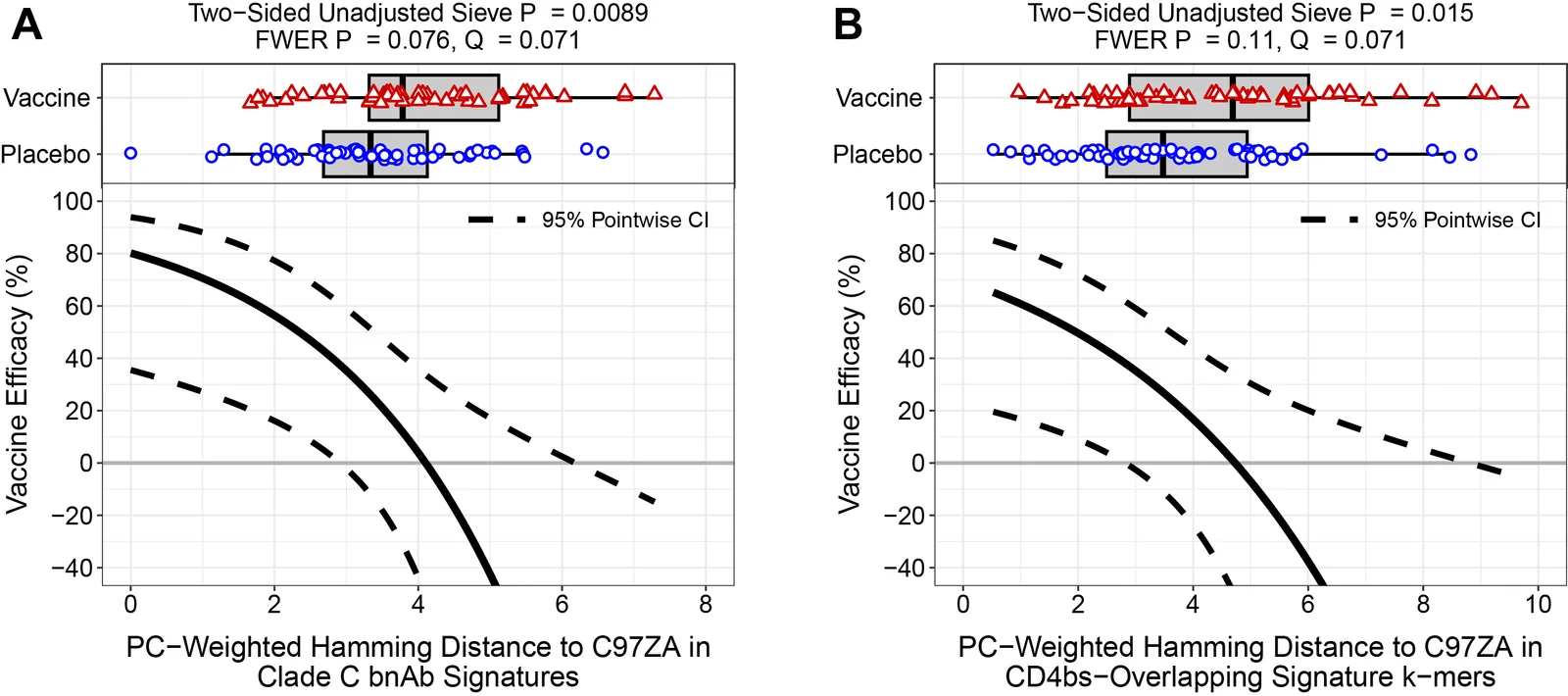
Vaccine efficacy (VE) against viruses with specific physicochemical (PC)-weighted Hamming distances from the C97ZA Env vaccine insert sequence, based on single representative sequences (mindist). Plots are for Hamming distances of (A) clade C bnAb resistance-associated signature positions and (B) CD4 binding site-overlapping HVTN 505 signature k-mers. The dashed black curves represent 95% pointwise confidence intervals. The horizontal box plots at the top show distributions of the PC-weighted Hamming distances in primary endpoint cases in the vaccine group (open red triangles, n = 53) and the placebo group (open blue circles, n = 63), where the vertical line in each boxplot represents the median PC-weighted Hamming distance and the boxes extend through the 25th and 75th percentiles. “Unadjusted Sieve P” two-sided P-values from testing whether VE varies with the sequence distance on the x-axis are also shown [A: P = 0.0089 (Wald test); B: P = 0.015 (Wald test)].

Prior sieve analyses demonstrated that aggregate measures of deviation from a reference sequence may discriminate VE more effectively than individual position-specific mutations.^18^^,^^19^ To this end, we prespecified Hamming distances of mindist sequences from vaccine insert sequences for key regions in Env, with position-specific AA substitutions weighted by their physicochemical (PC) properties. Multiple distances were designed to separately capture deviations in several hypothesis-driven position sets (Table S5). We found significantly declining VE with an increasing PC-weighted Hamming distance from the C97ZA vaccine insert in the set of 11 subtype C bnAb resistance signature positions [unadjusted P = 0.0089 (Wald test), FWER P = 0.08, Q = 0.07] (Fig. 3A). VE was estimated to be >60% against viruses with ≤2 mismatched residues to C97ZA [with 6 (10%) placebo endpoints and 4 (7%) vaccine endpoints having ≤2 mismatched residues], and it declined to 0% with the accumulation of 4 residue mismatches in this set [with 18 (26%) placebo endpoints and 26 (49%) vaccine endpoints having ≥4 mismatched residues] (Fig. 3A). In a post-hoc analysis, position 364 appeared to be influential on the VE decline as its exclusion resulted in loss of significance [unadjusted P = 0.06 (Wald test)], albeit a trend of a declining point estimate of VE remained (Fig. S8). For the distances from each of the Mos1 and Mos2S vaccine sequences in the same position set, the decreasing trend in VE was not significant and appeared to be driven by position 364 for the Mos1 reference (Fig. S9). In this set (Fig. S10), position 364 was the only one that exhibited a difference in Shannon entropy between the placebo and vaccine groups [–0.48 (95% CI, −0.87 to −0.079) as reported above].

VE also diminished significantly with an increasing PC-weighted Hamming distance from the C97ZA vaccine sequence in the set of 54 positions within CD4 binding site (CD4bs)-overlapping HVTN 505 signature k-mers^18^ [unadjusted P = 0.015 (Wald test), FWER P = 0.11, Q = 0.07] (Fig. 3B). VE was estimated to be >60% against viruses with ≤1 mismatched residue to C97ZA, and the accumulation of ≥5 residue mismatches in this set abrogated VE (Fig. 3B). The declining trend in the point estimate of VE with the same distance measure from each of the Mos1 and Mos2S vaccine sequences was not significant (Fig. S11). No position in this set showed an apparent difference in Shannon entropy between vaccine and placebo groups (Fig. S12). None of the remaining PC-weighted Hamming distances, either from an Env vaccine sequence or the gp70-001428.2.42 V1V2 antigen sequence associated with the strongest IgG3 V1V2 correlate, were found to discriminate VE (Figs. S13 and S14).

### No evidence of variation in VE with features of Env variable loops

HIV-1 Env hypervariable regions vary across individuals and over time within a single host^59^, ^60^, ^61^ due to insertions and deletions that often repeat multiple times within the same region. These result in changes in length, charge, and the number of PNGS within the hypervariable regions of Env that mediate bnAb escape^44^^,^^62^ and transmission fitness in subtype C acquisitions.^63^ We thus prespecified five Env variable loop features shown to be associated with changes in bnAb resistance to be tested for their association with changes in VE: length of V1V2, number of PNGS in V1V2, length of the V5 loop, number of PNGS in V5, and electrochemical charge in V2, all calculated in mindist sequences.^44^ We found no evidence of a variation in VE with distinct levels of any of these features, with all Q-values ≥0.60 (Figs. S15 and S16).

### No evidence of variation in VE with cysteine count in Env gp160

Motivated by the finding of significantly higher Env cysteine counts in vaccine recipients from the HVTN 505 trial,^18^ we prespecified Env gp160 cysteine count in the mindist sequence as a feature of interest and found no evidence of variation in VE with this feature [unadjusted P = 0.25 (Wald test)] (Fig. S15D).

### No evidence of variation in VE with early virus lineage multiplicity

No evidence was found for a difference in VE against HIV-1 diagnosis with a single vs. multiple early virus lineages (Fig. S17).

### No evidence of Env structural divergence from C97ZA in the vaccine group

Analysis of 115 15-mer TM-scores, tiled across the gp120 and gp41 regions, of AlphaFold3-predicted Env structures of mindist sequences revealed no regions with a significant difference in structural similarity to the C97ZA insert sequence-predicted structure between vaccine and placebo endpoint cases (all Q-values ≥0.60; Fig. S18). Two top-ranking 15-mer windows were 477–491 and 231–239; both showing a nominal trend towards greater divergence from the C97ZA structure among vaccine cases compared with placebo cases [unadjusted P = 0.012 and 0.023, respectively (linear regression permutation tests)].

### Low intra-host sequence feature variability

Most sieve analyses to date have used the single mindist Env AA sequence as a representation of the depth of sequences measured from a primary endpoint case, raising the opportunity to gain additional insights by including the full sequence set. To assess this possibility, we utilised the deep sequence data to characterise intra-host sequence variability of each hypothesis-driven feature in the gp120 portion of Env (Figs. S19 and S20). Of the 144 screened-in tier 1 binary features, 4% had zero intra-host variability, 43% had >95% of endpoint cases with zero variability, and 99% had >80% of cases with zero variability. Among cases with any intra-host variation in values of a given feature, the proportion of intra-host sequences with a binary value other than the dominant one ranged between 0.7–0.9% to 0.2–48% per feature. Of the 11 quantitative features, one had >50% and nine had >25% of cases with zero variability outside the modal neighbourhood, with the modal neighbourhood defined as an individual's modal value of a feature −/+ 0.5 times the average of the feature's intra-host sample standard deviations. Among cases with variability outside the modal neighbourhood, the proportion of intra-host sequences outside the neighbourhood ranged between 0.2–16% to 0.6–75% per feature. In sum, intra-host feature variability was very low, supporting that, for most features, conclusions drawn from the single-sequence analyses robustly reflect all the available information in the sequence data.

### Higher VE against viral populations with ≥99% of sequences with leucine at Env position 832

**Fig. 4:**
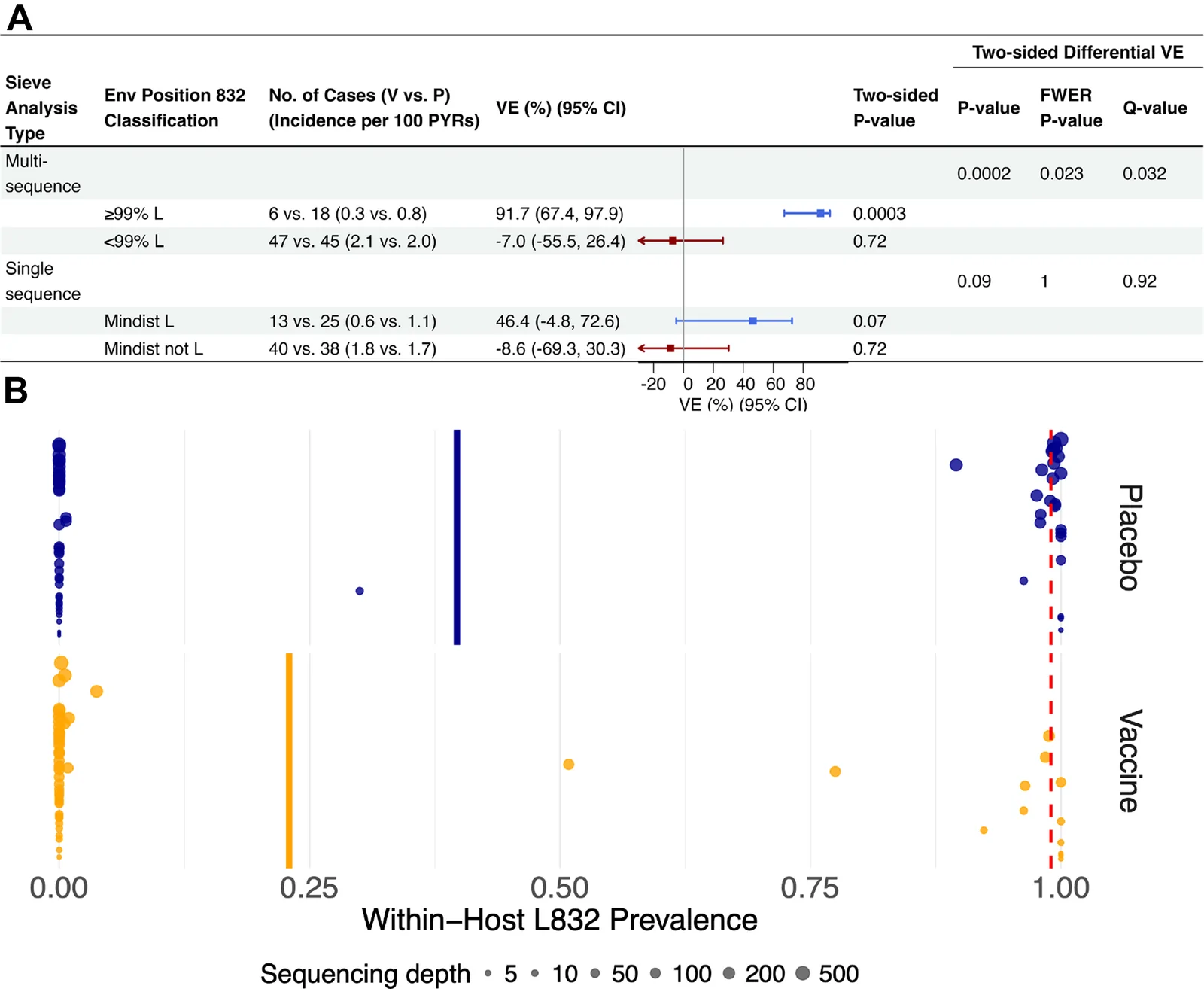
Sieve analyses of Env HXB2 position 832. (A) Vaccine efficacy (VE) against viral populations with ≥99% vs. <99% prevalence of leucine at Env position 832 (L832), contrasted with VE against viral populations whose single representative (mindist) sequence carries L832 vs. not-L832. Sequences having a gap at position 832 were included in the analysis; no gaps were present in the mindist sequences. In both the multi-sequence and single-sequence analysis, primary endpoints analysed were n = 53 vaccine, n = 63 placebo. In the multi-sequence analysis, all P-values are from competing risks Cox model Wald tests, with bootstrap covariance estimates as described in a preprint.^54^ In the single-sequence analysis, P-values in the “Two-sided P-value” column are from the competing risks Cox model Wald test and those in the “Two-sided Differential VE P-value” column are from the Lunn-McNeil test.^50^ P-values for differential VE were P = 0.0002 in the multi-sequence analysis and P = 0.09 in the single-sequence analysis. Filled squares designate VE point estimates, and bars extend through the 95% confidence intervals (CIs). (B) Observed L832 prevalence among sampled sequences from primary endpoints by treatment group (n = 53 vaccine, n = 63 placebo). Data point size is proportional to sequencing depth. The solid vertical lines show the average treatment-specific observed L832 prevalence.

A small number of binary features exhibited sufficient variability to warrant an assessment of whether VE depended on the overall composition of the viral population rather than solely on a single representative sequence. This analysis incorporated all measured sequences per participant, accounted for differences in sequencing depth, and evaluated VE against viral populations defined by thresholded binary-feature prevalence in the whole viral quasispecies in blood (≥1% vs. <1%, and ≥99% vs. <99%) from which the sequence data are a sample. Of the 568 binary tier 1 features, none met the predefined screening criteria (see Methods) for either the 1% or 99% threshold analyses (Table S6). Of the 7890 binary tier 2 features, 114 passed the screening steps, including 71 for 1% threshold analysis and 43 for 99% threshold analysis. In the multi-sequence analysis, we found evidence of differential VE against HIV-1 quasispecies with ≥99% prevalence of leucine at Env HXB2 position 832 in the cytoplasmic tail of gp41 (VE = 91.7%; 95% CI, 67.4–97.9) vs. those with <99% prevalence of leucine at that position (VE = −7.0%; 95% CI, −55.5 to 26.4) [unadjusted P = 0.0002 for differential VE (competing risks Cox model Wald test with bootstrap covariance estimate), FWER P = 0.023, Q = 0.032] (Fig. 4A). Vaccine cases tended to have a lower observed L832 prevalence among sampled sequences, and those with ≥99% prevalence generally exhibited lower sequencing depth than placebo cases (Fig. 4B). The VE estimation method, however, explicitly corrected for potential sequencing depth-related misclassification; therefore, the L832 signature cannot be explained as an artefact of treatment differences in sequencing depth.

While the functional filter excluded AA sequences with a premature stop codon located more than 5% of the gene length from the 3′ end, it permitted inclusion of sequences with stops occurring from position 814 onward. Of the total 37,211 sequences analysed, 264 contained a gap at position 832: 54 (21%) had a gap due to a deletion [32 from a single placebo endpoint (9% of 377 sequences total)], and the remaining 210 sequences due to a premature stop codon prior to position 832. To investigate whether the L832 signature was driven by alignment artefacts, we performed a sensitivity analysis by excluding all sequences with a gap at position 832. We observed a comparable result, with VE = 87.3% (95% CI, 49.0–97.0) against quasispecies with ≥99% prevalence of L832 and VE = −22.4% (95% CI, 85.1–19.0) against those with <99% prevalence of L832 [unadjusted P = 0.0024 for differential VE (competing-risks Cox model Wald test with bootstrap covariance estimate)] (Fig. S21), indicating that the signal was robust to potential alignment artefacts.

The single-sequence analysis—based on mindist sequences, which exhibited no gaps at position 832—showed a VE trend in the same direction [VE against mindist L832 = 46.4% (95% CI, −4.8 to 72.6) vs. VE against mindist not-L832 = −8.6% (95% CI, −69.3 to 30.3); unadjusted P = 0.09 for differential VE (Wald test); Fig. 4A]. Hence, the relative risk ratio (≥99% vs. <99% whole-quasispecies prevalence of L832) in the multi-sequence analysis (0.078) was estimated to be 6.4-fold smaller than the relative risk ratio (L832 vs. not-L832) in the single-sequence analysis (0.49), indicating that the sieve effect size was larger and power to detect the sieve effect was higher when the full viral population composition was accounted for. To further explore this finding, we assessed in a post-hoc analysis whether vaccination influenced intra-host residue diversity at this position. The comparable mean intra-host Shannon entropy among placebo endpoints [0.15 (95% CI, 0.071–0.22)] and vaccine endpoints [0.13 (95% CI, 0.060–0.20); difference = 0.01 (95% CI, −0.092 to 0.11)] suggests that the sieve effect likely reflects post-infection adaptation rather than transmission bottleneck. Together, these results suggest that subtle shifts in viral population structure, rather than mindist sequence differences, may underlie the differential vaccine effect on HIV-1 linked to leucine at Env position 832.

### No strong evidence of vaccine-placebo separation in high-dimensional feature analysis

Application of the Direction-Projection-Permutation (DiProPerm) method to the high-dimensional single-sequence feature data revealed no material evidence of separation between vaccine and placebo endpoints across 97 analysed feature subsets (Table S7). The smallest global P-value was 0.029 (DiProPerm permutation test), observed for subset 2e, which comprised 15 vaccine residue match/mismatch features at signature positions for clade C bnAbs. A comparable P-value [0.035 (DiProPerm permutation test)] was obtained for the corresponding subset 2e hybrid, which combined subset 2e with the region-specific structural features (subsets 13o and 13p). The loadings for subset 2e are shown in Fig. S22. Notably, the top six loadings were identical for subsets 2e and 2e hybrid, indicating that inclusion of the TM-score structural features did not strongly influence treatment classification. The feature with the highest loading was the indicator of vaccine residue match at Env position 364, consistent with the independent sieve finding highlighting the importance of position 364 as a site of potential vaccine-induced selection. Although the P-values for subsets 2e and 2e hybrid reached nominal significance at the 0.05 level, they do not constitute strong evidence against the global null hypothesis given that 97 feature subsets were tested. None of the remaining 95 subsets achieved nominal significance.

## Discussion

The main finding of this analysis of Env AA sequences in 116 HVTN 705 trial participants who acquired a primary endpoint (53 vaccine; 63 placebo) is a lack of variation in VE with the majority of the analysed Env sequence features. There was no evidence for variation in VE with Env sequence features restricted to AA positions linked to immune correlates identified in a nonhuman primate challenge model (rhesus monkeys were immunised with Ad26/Ad26+gp140 and then challenged repeatedly with the heterologous SHIV-SF162P3 strain^41^). Similarly, we found no evidence for variation in VE with Env sequence features restricted to AA positions linked to the potential immune correlate signal—V1V2 IgG3 binding antibodies—in the HVTN 705 trial itself.^12^ We further analysed Env signature positions that were previously shown in the RV144 HIV-1 vaccine efficacy trial to impact VE in sieve analysis^15^ or in immune correlates and experimental analyses,^23^^,^^42^^,^^64^ and found no signal of any sieve effects pertaining to these signatures. This may be due to the lower levels of V1V2-specific antibodies in HVTN 705 compared to RV144,^65^ or our current lack of knowledge of the conformational epitope associated with the RV144 V1V2 effect. Moreover, no evidence of variation in VE was found for signature glycan or viral geometry features previously found to be associated with resistance against the most common bnAb classes. There was no evidence of variation in VE by whether the isolated virus included multiple lineages, nor any signal of AlphaFold3-predicted structural divergence from vaccine Envs. Thus, Env-based sieve effects were largely absent from the HVTN 705 trial.

This analysis also yielded weak and inconclusive signals in support of sieve effects that, while cautious interpretation is warranted, are nevertheless worth discussing. When analysing solely a single representative sequence, evidence for differential VE was observed across levels of three prespecified Env features. The first was physicochemical-weighted Hamming distance between a viral sequence and a reference vaccine sequence across a combined set of four CD4bs-overlapping signature regions identified by a k-mer scanning analysis in the HVTN 505 sieve analysis.^18^ While VE decreased against viruses with increasing distance from the CD4 binding site k-mers in both trials, evidence for the sieve effect was weaker, or not significant, in HVTN 705 compared to HVTN 505. For example, the unadjusted P-value was 0.015 for distance from the clade C gp140 (C97ZA) reference sequence in HVTN 705 (this paper) vs. an unadjusted P-value of 0.00046 for distance from the clade B gp120 reference sequence in HVTN 505 (deCamp et al.^18^). Moreover, VE did not significantly decrease with increasing distance from the Mos1 and Mos2S Ad26 Env vaccine inserts in HVTN 705 (unadjusted P-values of 0.10 and 0.09, respectively). Given that the Ad26 vector vaccine delivering the four mosaic immunogens was administered more frequently (four immunisations) than the gp140 protein vaccine (two immunisations) in HVTN 705, this difference between the C97ZA-based and mosaic-based results is perhaps unexpected. This asymmetry is consistent with, but does not establish, a more prominent role of antibody responses linked to the gp140 protein vaccine than antibody responses linked to the mosaic vector components of the vaccine regimen.

The second feature associated with VE also pertained to divergence from the clade C gp140 (C97ZA) sequence, where VE significantly decreased against viruses with increasing PC-weighted Hamming distance from the C97ZA sequence in the set of subtype C bnAb resistance signature positions. Connectedly, the third feature associated with VE concerned position 364 in the CD4 binding loop, which exhibited a borderline-significant, local sieve effect: VE was higher against viruses harbouring the C97ZA sequence-matched residue S than against viruses with a mismatched residue. Given that the FWER-adjusted P-value was 0.22 for this position (and 0.32 using the alternative alignment), this analysis does not provide sufficient standalone evidence for a sieve effect; however, three additional results in the present work (the elevated Shannon entropy at position 364 in vaccine vs. placebo group mindist sequences, the identification of position 364 as influential in the subtype C bnAb signature set, and the identification of vaccine residue match at position 364 as the feature with the highest loading in the Direction-Projection-Permutation analysis) cumulatively support position 364 as a hypothesis-generating signal. Given that position 364 did not satisfy the prespecified screening criterion for sufficient intra-host diversity, it was not carried forward into the multi-sequence quasispecies analysis. Thus, the absence of a reported multi-sequence result for position 364 should not be interpreted as a failure to replicate the finding in that framework. Rather, under our prespecified screening rules, a multi-sequence analysis at this position was not expected to add meaningful information beyond the single-sequence analysis. Beyond the present work, position 364 was identified as a contact site for five additional CD4bs-specific bnAbs (HJ16, VRC13.01, VRC16.01, CH103, b12)^66^, ^67^, ^68^, ^69^ that did not contribute to the subtype C bnAb signature set used here, and S364 was also associated with sensitivity to CD4bs-targeting bnAbs in a bnAb signature mapping effort.^44^ Therefore, while the overall evidence is weak and non-definitive for a position 364 sieve effect, the recurring signal across complementary analyses generates hypotheses that need confirmation in future studies.

An additional finding of this study is that, when analysing the overall composition of the viral population, VE was significantly higher against quasispecies with ≥99% (vs. <99%) of sequences having a leucine residue at position 832. This result is consistent with the idea that a small minority of vaccine-resistant variants in the quasispecies can thwart vaccine protection. However, while the observed association with differential VE was statistically significant, it depends on the prespecified 99% threshold used to classify viral populations by L832 prevalence. Moreover, given that position 832 resides within the gp41 cytoplasmic domain,^70^ which was not included in the Ad26 vaccine Env sequences nor in the gp140 vaccine sequence, the L832 finding does not support a straightforward interpretation based on direct vaccine-induced antibody or T-cell pressure at that site. Instead, the L832 sieve signal may have arisen indirectly, for example through functional or structural interdependence among Env sites, compensatory evolution that preserves viral fitness, or post-acquisition adaptation linked to immune pressure exerted elsewhere in Env. Given the complete lack of signal at other residues near position 832, however, such potential explanations remain speculative in the present setting. Serum antibody binding assays, neutralising antibody assays, or antibody-dependent cellular cytotoxicity experiments stratified by L832 prevalence, or employing site-directed mutagenesis at position 832, could potentially help gain insights into whether/how L832 is actually linked to immunological mechanisms of protection or selection.

Another finding of this study was a trend of lower viral load in the first RNA-positive sample in the vaccine versus placebo group, with the estimated geometric mean viral load in the vaccine group approximately half that in the placebo group. This trend diminished over 6 weeks following the first detection of viral RNA, and a post-hoc longitudinal analysis of pre-ART viral loads suggested a faster post-detection decline in placebo than vaccine recipients. Because viral load directly influences the amount of material available for sequencing, it was not unexpected that the number of measured intra-host nucleotide sequences tended to be lower in the vaccine group, yet it is interesting that, in these data, sequencing depth was a more sensitive metric of the vaccine slowing viral replication than viral load itself. Moreover, the reduction in sequencing depth was even more pronounced for functional AA sequences. Whereas nucleotide sequence depth reflects the efficiency of sequencing at a given viral load, AA sequence depth, due to the application of the functional filter (see Methods), reflects the virus's reproductive capacity at that viral load, suggesting that vaccine-induced immune responses exerted selective pressure on the virus to maintain reproductive fitness, i.e., to avoid mutations that would yield non-functional variants.

Addressing the question as to whether the vaccine impacted the genetic bottleneck on breakthrough viruses, we found no evidence for a treatment difference in the frequency of multiple early virus lineages or in inter-lineage divergence among primary endpoints with multiple virus lineages. For most primary endpoints across both treatment groups, the inter-lineage divergence was greater than the expected divergence that would accumulate post-acquisition considering the number of days since the last RNA-negative sample collection and assuming an average per-nucleotide mutating rate of 2.16 × 10^−5^ per generation cycle.^28^^,^^33^^,^^35^ However, it is possible that, for some primary endpoints, the observed multiple virus lineages evolved post-acquisition rather than being separate transmitting lineages, especially given the long (3-month) interval between sampling in this trial. This can happen due to early, potentially stochastic mutations that happen shortly after acquisition, outgrowth of fitter transmitted variants, or due to early onset of immune pressure escape, which is usually localised to T-cell epitopes where a higher accumulation of mutations is observed as the virus reacts to the host's first T cell immune responses.

Among single-lineage endpoints, intra-host env diversity was lower in the vaccine group than in the placebo group after adjustment for time since the last HIV-1 RNA-negative sample. Among multiple-lineage endpoints, the adjusted comparison did not show evidence of a treatment difference; however, this metric combined within-lineage diversity and between-lineage divergence, limiting direct comparison with the single-lineage result.

A strength of this study is the fact that this was a sieve analysis of a randomised, placebo-controlled vaccine efficacy trial, thus lending rigour and minimising potential confounding. An additional strength is the inclusion of multi-sequence sieve analyses, which fully leveraged the deep sequencing data to capture the overall composition of the viral population and revealed additional insights that would have been missed if the analyses had been limited to single-sequence analyses. Limitations of this study include that the low point estimate of overall VE constrained the magnitude of possible sieve effects, thereby limiting the statistical power of sieve analyses to detect differential VE. Moreover, for many binary Env AA features, the power to detect differential VE across the two feature levels was constrained by a small number of HIV-1 endpoint cases exhibiting one of the two levels. Additionally, analyses of the static AF3-predicted structures were limited given that these analyses did not account for dynamic motion, and the highly flexible hypervariable loop positions were excluded. This limitation could be addressed through additional work using molecular dynamics, which we are exploring in future work. Regarding generalisability, given that the HVTN 705 trial was conducted in southern African women, the results may not generalise to men and/or other geographic regions/epidemic contexts. The HVTN 706/HPX3002 trial tested the efficacy of a closely related mosaic HIV-1 vaccine regimen in men who have sex with men in the Americas and Europe.^71^

This work focused on examining VE variation with Env sequence features. It may be viewed as somewhat discouraging that, for this mosaic HIV-1 vaccine regimen, there was little evidence of an Env-based sieve effect. However, the absence of convincing Env-based sieve signals does not rule out vaccine-induced selection operating through other mechanisms, including T-cell-mediated effects. Work is ongoing to examine post-acquisition sieve effects that includes data on vaccine-induced T-cell responses and their restriction by HLA alleles.

## Contributors

Conceptualisation: MJ, LL, SB, FT, LCorey, KM, GEG, PBG, CW, JIM.

Data curation: MJ, LL, CAM, JP, EEG, JL, ACdC, JJK, CM, MM, PTE, RR, DHW, WD, LChen, PBG, CW, JIM.

Formal analysis: MJ, LL, CAM, EEG, JL, CM, JP, ACdC, JJK, MM, PTE, RR, DHW, WD, LChen, HZ, FT, PBG, CW, JIM.

Funding acquisition: LCorey, GEG, PBG.

Investigation: BG, CA, AY, DM, TY, AG-N, NN, RT, EMS, JAH, JvD, SB, FT, LCorey, KM, GEG, PBG, CW, JIM.

Methodology: MJ, LL, CAM, JP, EEG, JL, ACdC, JJK, PTE, RR, DHW, WD, LChen, PBG, CW, JIM.

Resources: EMS, JAH, JvD, SB, FT, LCorey, KM, GEG, PBG, CW, JIM.

Software: MJ, LL, CAM, JP, EEG, JL, PBG.

Supervision: MJ, CAM, JAH, JvD, SB, FT, LCorey, KM, GEG, PBG, CW, JIM.

Validation: MJ, LL, CAM, EEG, JL, PBG, CW, JIM.

Visualisation: MJ, LL, CAM, JP, EEG, JL, ACdC, JJK, CM, MM, PBG, CW, JIM.

Writing—original draft: MJ, LL, LNC, PBG.

Writing—review and editing: All coauthors.

MJ, LL, CAM, and PBG accessed and verified the underlying data reported in the manuscript. PBG was responsible for the decision to submit the manuscript. All authors read and approved the final version of the manuscript.

## Data sharing statement

The data sharing policy of Johnson & Johnson is available at https://innovativemedicine.jnj.com/our-innovation/clinical-trials/transparency. As noted on this site, requests for access to the study data can be submitted through Yale Open Data Access [YODA] Project site at http://yoda.yale.edu. All *R* code generated for the statistical analysis, including code used for deriving features of the sequences, is publicly available at https://github.com/HVTN-SDMC/705sieve-public.

## Declaration of interests

LNC was compensated for work on this manuscript, and received support to travel to the HVTN Full Group Meeting, through a consulting agreement with Fred Hutchinson Cancer Center. JvD was an employee of Johnson & Johnson at the time the work was conducted. FT was a full-time employee of Johnson & Johnson from the time that the concept of this trial was discussed and continued in the position of Clinical Franchise Leader of HIV Vaccines at Janssen Vaccine/Johnson & Johnson until the conclusion of the trial. FT was involved in writing of the protocol, protocol amendments, data analyses, and interpretation. FT also holds stock in Johnson & Johnson, some of which was granted during planning, execution, and analysis of this study. CW reports receipt of ThermaStop-RT to the University of Cape Town from ThermaGenix Inc., free of charge. All other authors declare no competing interests.

## Appendix B Supplementary data

Supplementary Tables and Figures

Statistical Analysis Plan
