# Supplementary material for: Quantifying how HIV-1 envelope sequence features impact vaccine efficacy in the HVTN 705/HPX2008 randomised trial in southern African women
Source: eBioMedicine. 2026 Aug 31;131:106459. doi: 10.1016/j.ebiom.2026.106459 (PMC13560528; doi:10.1016/j.ebiom.2026.106459)
Supplement: Supplementary file 1 [file mmc2.pdf]

Statistical Analysis Plan for Gentoypic Sieve Analysis in HVTN 705/VAC89220HPX2008

Version 4.0

April 17, 2026

PPD [redacted]

Fred Hutchinson Cancer Center and Janssen Statistics

Contents

1 **Version History and Summary of Changes** **5**

2 **Introduction** **5**

2.1 Scope of the first sieve analysis reported in May of 2022 6

3 **Vaccine insert sequences for sieve analysis** **6**

4 **Study Cohorts** **7**

5 **Time-to-Event Endpoint Definition** **8**

6 **Sieve Analysis Objectives from the Protocol** **8**

7 **Pacbio Sequence Data** **9**

8 **Process for the AA Sequence Sieve Analysis** **10**

8.1 Removing Unalignable Sites from the Sieve Analysis 13

8.2 Structuring AA Sequence Features for Sieve Analysis 14

8.2.1 Summary of All Analyzed Tier 1 and 2 AA Sequence Features and Associated Statistical Approaches 14

8.3 Exploratory Tier 2 Coverage Analysis 16

8.4 Exploratory Tier 2 Analysis of Signature Sites for Clade C bnAbs 18

8.5 Sieve Analysis Inference Conditioning on Infection (Approach A Methods) . 18

8.6 Accounting for Multiplicity of Sequences from Participants Who Acquired HIV-1 18

8.7 Application of Approach A and B Sieve Analyses to the AA Sequence Features in Tier 1 and Tier 2 19

8.8 Approach A Conditional Sieve Analysis Statistical Method 20

8.9 Details of Implementation of Approach B Proportional Hazards Sieve Analysis 22

8.10 Analysis of Indicator of Multi-Lineage Infection 22

8.10.1 Handling Non-Subtype C Acquisition Primary Endpoints 23

8.11 Descriptive Analysis of Amino Acid Sequence Features V_{obs} 23
8.12 Phylogenetic Trees 23
8.13 Multiple Hypothesis Testing Adjustment for Sieve Effects 23

9 R Code for the Sieve Analysis Methods 24

10 Additions to the v1 SAP: New Sieve Analyses Implemented in the v2 SAP 25
10.1 Additional Env Sequence Features 25
10.2 Complete List of Env Sequence Features for v2 SAP 26
10.3 Multiple Hypothesis Testing Adjustment for v2 SAP 26

11 Additions to the v2 SAP: New Sieve Analyses Implemented in the v3 SAP 29
11.1 Analysis of Predicted Env Structures for Functional Sieve Effects 29
11.2 Sieve Analysis of High-Dimensional Signals in Amino Acid Sequence Features 30

12 Revision from SAP v3.0 to v4.0 33

References 34

List of Tables

1 Sites to Exclude from Sieve Analyses Due to Unalignability 13

2 List of AA Sequence Features V_{obs} for Sieve Analysis 17

3 Application of Approach A and B sieve analysis to the AA sequence features in Tier 1 and Tier 2[1,2] 20

4 R code for implementing the sieve analyses* 25

5 Complete list of AA sequence features V_{obs} studied in v2 SAP, with additions in v2 shown in blue 28

1 Version History and Summary of Changes

The original sieve analysis statistical analysis plan (SAP) version 1.0 was finalized in May of 2022.

Version 2.0 was finalized in March 2023. Section 10 documents changes from version 1.0 to version 2.0.

Version 3.0 was finalized on June 10, 2025. Section 11 documents changes from version 2.0 to version 3.0.

Version 4.0 was finalized on April 17, 2026. Section 12 documents changes from version 3.0 to version 4.0.

2 Introduction

This SAP considers "mark variables", denoted as V_{obs}, for an HIV-1 feature marker in a study participant experiencing the primary HIV-1 infection endpoint. More specifically, V_{obs} is defined as an observed feature of acquired virus isolates from the first HIV-1 RNA positive sample. We use the notation V_{obs} to emphasize that it is an observed feature, and not necessarily a feature of breakthrough infecting viruses at the time of HIV-1 acquisition (because HIV-1 sequences at acquisition cannot be directly observed, features derived from sequences at acquisition are not directly observable).

Analyses in this SAP restrict to assessment of directly observed AA sequence feature marks V_{obs}, and restrict to the Env protein. More specifically, since the vaccine inserts cover gp120, some analyses will be limited to the gp120 protein for the purpose of optimzing our statistical power. As such, the AA sequence sieve analyses specified in this SAP focus on addressing the question of how vaccine efficacy VE varies with Env gp160 AA sequence characteristics of observed virus features. The sieve analyses also address a related question whose answers have a different interpretation: Among primary endpoint HIV-1 infected cases, how do Env gp160 AA sequence characteristics differ over the randomized vaccine vs. placebo arms?

While this SAP is developed to focus on observed sequence features and thus accepts that results cannot be definitively interpreted in terms of discriminating acquisition sieve effects from post-acquisition sieve effects, at a later point in time additional exploratory analyses may be planned that seek to explicitly isolate these two distinct types of sieve effects.

The main AA sequence sieve analyses focus on primary endpoint HIV-1 infected cases, with diagnosis of infection by the Month 24 study visit.

All Env protein positions mentioned in this SAP are in the context of HXB2, unless otherwise specified.

2.1 Scope of the first sieve analysis reported in May of 2022

Four main categories of sieve analysis are:

1. Descriptives of AA sequence features by treatment arm,
2. Prospective differential vaccine efficacy sieve analyses (named "Approach B"),
3. HIV-1 infection-conditional sieve analysis (names "Approach A"), and
4. machine learning sieve analysis that studies how AA sequence features best classify HIV-1 infected participants by assignment to vaccine or placebo.

The first sieve analysis report will include 1. and 2., where for 2. only the proportional hazards-based implementation will be used (not the cumulative incidence-based implementation). In addition, the first sieve analysis restricts to Env gp120 AA sequences; sieve analyses are not conducted to assess differential vaccine efficacy by Gag-Pol-Nef AA sequences. Once HLA type data are available, T cell epitope-based sieve analyses will be applied to conduct sieve analysis of Gag-Pol-Nef sequences. Additionally, some follow-up analyses may consider the Env gp41 protein. Lastly, the first sieve analysis restricts to the analysis of a single sequence per primary endpoint HIV-1 infected participant, and a potential vaccine effect on modifying the probability of multi-lineage infection is studied based only on the indicator of multi-lineage infection; later analyses can assess multi-lineage infection accounting for the number of inferred lineages. (The term 'lineage' denotes a set of sequences inferred to have evolved from a single founder sequence.)

3 Vaccine insert sequences for sieve analysis

The vaccine insert for the quadrivalent Ad26.Mos4.HIV vaccine includes the following mosaic immunogens:

1. Mos1 Gag-Pol (Ad26.Mos1.Gag-Pol.v3, plasmid ID 1857)
2. Mos2 Gag-Pol (Ad26.Mos2.Gag-Pol.v3, plasmid ID 1858)
3. Mos1 Env (Ad26.Mos1.Env, plasmid ID 1817)
4. Mos2 Env (Mos2S.Env)

The follow-up adjuvanted Clade C gp140 product is based on the C97ZA strain and is formally known as"C97ZA_fib_SortA".

The initial sieve analyses are done for each of the three Env insert sequences (the two mosaic inserts and the clade C strain) used as the reference sequence.

4 Study Cohorts

The following study cohorts or analysis sets are used for addressing the sieve analysis objective. The terminology for the cohorts is also used in the protocol and in the safety and efficacy statistical analysis plan.

1. **Modified Intent-to-Treat (MITT) cohort:** participants in the Full Analysis Set (FAS) who are HIV-1 uninfected on the date of first vaccination.

2. **Per-Protocol (PP) cohort:** participants in the FAS who are HIV-1 uninfected 4 weeks after the 3rd vaccination visit, who received all planned vaccinations at the first 3 vaccination visits within the respective visit windows and have no other major protocol deviations that were judged to possibly impact the efficacy of the vaccine. A participant who received the 3rd vaccination is considered HIV-1 uninfected 4 weeks after the 3rd vaccination visit if the last negative sample collection date coincides or follows the 3rd vaccination visit date plus 28 days.

3. **Full Immunization Set (FIS):** participants in the FAS who are HIV-1 uninfected 4 weeks after the 4th vaccination visit and who receive all planned vaccinations within the respective visit windows. A participant who received the 4th vaccination is considered HIV-1 uninfected 4 weeks after the 4th vaccination visit if the last negative sample collection date coincides or follows the 4th vaccination visit date plus 28 days.

4. **MITT HIV-1-Diagnosed by 24 Months Cohort (MITTcases0to24):** Participants in the MITT cohort who are diagnosed with HIV-1 infection during the follow-up period after enrollment through the Month 24 visit.

5. **MITT HIV-1-Diagnosed by 36 Months Cohort (MITTcases0to36):** Participants in the MITT cohort who are diagnosed with HIV-1 infection during the follow-up period after enrollment through the Month 36 visit.

6. **Per-Protocol HIV-1-Diagnosed by 24 Months Cohort (PPcases7to24):** Participants in the IC-PP cohort who are diagnosed with HIV-1 infection during the follow-up period on or after the Month 7 visit through the Month 24 visit.

7. **Per-Protocol HIV-1-Diagnosed by 36 Months Cohort (PPcases7to36):** Participants in the IC-PP cohort who are diagnosed with HIV-1 infection during the follow-up period on or after the Month 7 visit through the Month 36 visit.

8. **Full Immunization Set HIV-1-Diagnosed by 24 Months Cohort (FIScases13to24):** Participants in the FIS cohort who are diagnosed with HIV-1 infection during the follow-up period on or after the Month 7 visit through the Month 24 visit.

9. **Full Immunization Set HIV-1-Diagnosed by 36 Months Cohort (FIScases13to36):** Participants in the FIS cohort who are diagnosed with HIV-1 infection during the follow-up period on or after the Month 7 visit through the Month 36 visit.

For time-to-event sieve analyses, the PP cohort is the cohort for analysis. For infection-conditional sieve analyses, PPcases7to24 is the cohort for analysis.

5 Time-to-Event Endpoint Definition

In all time-to-event endpoint sieve analyses such as those of estimation and inference about parameters $VE_h(v)$ and $VE_c(\tau, v)$ introduced in Section 8, the event of interest will be the collection of the first HIV-1 RNA positive sample as determined by the standard HIV-1 RNA PCR assay. For participants who do not register the HIV-1 endpoint, their follow-up time will be right-censored at their last HIV-negative sample collection date.

In all hazard- and cumulative incidence-based analyses, follow-up times are right-censored at the same fixed time point τ as in the primary efficacy analyses, defined in the main HVTN 705 SAP v3.3 as 25.91 months post-enrollment representing the right edge of the month 24 allowable visit window.

Throughout the sieve analysis, we will analyze time-to-event data following database lock on April 29, 2022.

6 Sieve Analysis Objectives from the Protocol

The following two study objectives, copied from the protocol, are considered to be the "sieve analysis" study objectives, and the purpose of this statistical analysis plan (SAP) is to describe the statistical methods and their implementation that will be used to answer these study objectives. The endpoints listed in the protocol corresponding to these objectives are also recapitulated here.

Secondary Objective 8: If significant positive evidence of vaccine efficacy from month 7 through 24 months is seen, to assess if and how vaccine efficacy depends on genotypic characteristics of HIV such as signature mutations

Secondary endpoint 8: HIV-1 infection diagnosed after month 7 through Month 24 and genotypic characteristics of viral sequences from HIV-1-infected participants at HIV-1 diagnosis, such as signature site mutations.

Secondary Objective 9: To evaluate and compare genomic sequences of viral isolates from HIV-1-infected vaccine and placebo recipients, and use sieve analysis methods to assess whether VE differs by genotypic or phenotypic characteristics of exposing HIVs and whether there is evidence of vaccine-induced immune pressure on the viral sequences.

Secondary endpoint 9: Viral sequences from HIV-1-infected participants at the earliest available post-infection timepoint and possibly subsequent visits.

This SAP restricts to genotypic HIV-1 features, which is referred to as amino acid (AA) sequence sieve analysis.

7 Pacbio Sequence Data

Sequence data are available from each primary endpoint HIV-1 infected participant from the first positive sample.

In the HVTN 705 trial, UMI (universal molecular identifier)-tag based PacBio sequencing methodology was used to sequence the REN and GP gene regions of the HIV-1 genome from participants who became HIV-1 infected during the study. The PacBio SMRT UMI-tagged PacBio sequencing protocol was developed by the Mullins laboratory. The use of PacBio sequencing has two unique and valuable advantages. First, unlike the Sanger and Illumina sequencing platforms, it allows determination of the entire length of the *env* (and *gag*) genes in single sequence reads, and in doing so allows an assessment of changes-at-a-distance from the contact point with antibodies that may impact protein structure and thus antibody neutralization sensitivity (i.e., along the entire protein). Second, the use of UMIs as molecular barcodes that are added onto each viral genome as it is copied into cDNA, allows performance of accurate, single-genome sequencing, and at greater depth than has been achieved to date.

The laboratory procedures employed, developed in the Mullins laboratory, are unique in that they remove PCR and sequencing misincorporation errors. This is critical since the level of these errors typically exceed the natural degree of HIV population diversity early in infection. These procedures also greatly reduce the occurrences of recombination during PCR. In contrast, both mutation and recombination artefacts are common in typical PacBio as well as other deep sequencing approaches. These improvements are achieved by sequencing each molecule over 50 times (versus 1 time in typical PacBio experiments), which effectively eliminates PCR and sequencing errors. Also, generating the products to be sequenced over a large number of independent PCR reactions, usually 8 per sample (compared to the typical single reaction), nearly eliminates recombination artefacts.

In summary, these procedures permit an unprecedentedly deep and accurate view of emerging founder virus populations *in vivo*, including sensitive identification of variants of potentially differential antibody sensitivity. This accuracy is critical and necessary to discern virus population diversity in the nearly homogeneous virus populations that typically characterize acute HIV infection. Without these advanced procedures in place, misincorporation errors that occur during the PCR reactions would equal or exceed the number of mutations that have occurred in the virus population very early in infection and thus prevent accurate measurements of viral diversity and obscure the timing of infection.

This protocol was used in the Mullins and Williamson laboratories for the sequencing of plasma samples from all trial participants who acquired a primary endpoint HIV-1 infection.

The raw PacBio sequence data generated were pre-processed before expert driven alignment by a pipeline developed by Ben Murell and Jim Mullins which included a quality filter, de-multiplex of data according to sample, collapsing circular consensus sequences by UMI, removing sequences with CCS read number for each UMI below threshold for inclusion, preparing initial automated sequence alignments, manually reviewing nucleotide and amino acid alignments, manually inspecting and resolving issues with the data processing pipeline and preparing sequence reports.

8 Process for the AA Sequence Sieve Analysis

As stated in the introduction, the AA sequence sieve analysis focuses on the analysis of observed virus sequence features measured from samples at the first available HIV-1 RNA PCR positive time point. Sieve analysis questions are posed in two ways: first, the assessment of whether and how vaccine efficacy (VE) depends on AA sequence features at the first time point (prospective sieve analysis (Gilbert et al. (1998)) and, second, the assessment of whether and how these AA sequences differ among randomized treatment arms (conditional sieve analysis). The first kind is normative sieve analysis, given the direct interpretation in terms of vaccine efficacy, whereas the second kind can have improved statistical power and can provide additional insights, but does not have an interpretation in terms of differential vaccine efficacy. We arbitrarily refer to the first kind and second kind as Approach B and Approach A sieve analysis, respectively, following the nomenclature used for the neutralization sieve analysis (Corey et al., 2021). Because of the interpretation in terms of prospective vaccine efficacy, the Approach B sieve analysis results are prioritized.

For Approach B sieve analysis, we consider two Env gp160 AA sequence feature-specific VE estimands for measuring sieve effects:

1. $VE_h(v)$ defined as 1 minus the hazard ratio (vaccine/placebo) of the time-to-event endpoint through the month 24 visit with a specific univariate AA sequence feature $V_{obs} = v$ associated with the set of sequences measured from the infected participant's earliest HIV-1 RNA+ sample.

2. $VE_c(\tau, v)$ defined as 1 minus the ratio (vaccine/placebo) of cumulative incidences at time τ of the time-to-event endpoint with a specific univariate AA sequence feature $V_{obs} = v$ associated with the set of sequences measured from the infected participant's earliest HIV-1 RNA+ sample. The identical timepoint τ is selected as in the primary overall VE analysis.

An Approach B AA sequence sieve effect is defined as statistically significant evidence for differential VE across multiple levels of a given AA sequence feature. Note that p-values quantifying the sieve effect evidence will only be computed for the vaccine vs. placebo comparison. The primary estimand of interest is $VE_h(v)$, and sieve effect evidence will be

evaluated for every specified feature V_{obs} in vaccine vs. placebo comparisons with respect to $VE_h(v)$.

We use published sieve analysis methods for analyzing various types of AA sequence marks V_{obs}. For binary and ordered K-level categorical V_{obs} with $K > 2$ but not large (i.e., K in the range 3 to 4), the competing risks Cox model and the Lunn and McNeil (1995) method, studied in Gilbert (2000) for sieve analysis, are used for assessing VE_h against each of the K genotypes (with 95% confidence intervals, 2-sided p-values for $VE_h(v)$ departing from 0% for a given v, and a 2-sided p-value for differential $VE_h(v)$ for either $v = 0, 1$ or $v = 0, 1, 2, \cdots, K$). For a subset of binary and ordered categorical V_{obs}, the cumulative incidence estimator and its influence function-based variance estimator in Benkeser, Juraska, and Gilbert (2020) are used for assessing $VE_c(\tau, v)$ for each level v of V_{obs} (with 95% CIs, 2-sided p-values for $VE_c(\tau, v)$ departing from 0% for a given v, and a 2-sided p-value for a linear trend in $\log\{1 - VE_c(\tau, v)\}$ with v).

For quantitative V_{obs} with a large number of levels such as a weighted Hamming distance, the Juraska and Gilbert (2013) method is used along with the goodness-of-fit diagnostics for the modeling assumptions, which include a diagnostic of the proportional hazards assumption.

In addition, the nonparametric kernel smoothing method of Sun, Gilbert, and McKeague (2009) and Gilbert and Sun (2015) will be applied, where the smoothing may yield additional insights. The analysis will be implemented using the *cmprskPHContinMark* R code available at peter Gilbert's website, using the same tuning parameter settings as for the neutralization sieve analysis (Corey et al., 2021).

For K-category ordered marks with $K = 3$ or 4, the Aalen-Johansen (AJ) nonparametric maximum likelihood estimator (NPMLE) is used for assessing $VE_c(\tau, v)$ for each of the K levels v of V_{obs} (with 95% CIs, 2-sided p-values for $VE_c(\tau, v)$ departing from 0% for a given v, and a 1-sided p-value for a linear trend in $\log\{1 - VE_c(\tau, v)\}$ with v). The same analysis was done for neutralization sieve analysis (Corey et al., 2021).

If more than 10 percent of HIV infected cases are missing mark data, then the missing data versions of the methods will be used, which means replacing the competing risks Cox model and Lunn and McNeil (1995) with the AIPW method of Heng et al. (2022), and replacing Juraska and Gilbert (2013) with the AIPW method of Juraska and Gilbert (2016) If needed, these missing data methods will be implemented estimating the probability of observing the mark using a best-fitting logistic regression model selected by all-subsets model selection according to the BIC criterion; where the covariates considered are treatment, South Africa vs. not South Africa, and viral load at the earliest available time point. In addition, for each candidate auxiliary variable, linear or logistic regression will be used to relate the mean of the auxiliary variable to the variables retained in the "missing data model", treatment, and the mark, where again BIC is used to pick a best-fitting model. If less than 10 percent of HIV infected cases are missing mark data, then simpler complete-case implementations of the method will be used, which is the competing risks Cox model, Lunn and McNeil (1995), and Juraska and Gilbert (2013) as noted above. Based on the data, fewer than 10% of cases

are missing sequence data, such that the complete-case implementations will be used.

For analyses involving $VE_h(v)$, the competing risks Cox model, the duplication method Cox model underlying the Lunn and McNeil (1995) calculation, and the Cox model portion underlying the product estimator of Juraska and Gilbert (2013) will be stratified by South Africa vs. not South Africa (i.e., have a separate baseline hazard for South Africa and not South Africa), whereas the cumulative incidence estimator will be stratified by South Africa vs. not South Africa within each treatment group. For $VE_h(v)$, stratification is by separate baseline hazards (the mark density ratio portion of the product estimator is unstratified), and, for $VE_c(\tau, v)$, stratification is by separate treatment-specific cumulative incidence estimation in each stratum with an equal weight assigned to each stratum. For analysis of $VE_h(v)$ for each binary mark that is studied, for each of the two genotypes a p-value for the Grambsch and Therneau (1994) proportional-hazards diagnostic test is reported.

Approach A amino acid sequence sieve analysis is based on the PPcases7to24 as defined in Section 4. In contrast, Approach B sieve analysis is based on the PP cohort defined in Section 4. Approach B sieve analysis studies vaccine efficacy separately against specific HIV-1 genotypes and assesses whether vaccine efficacy varies across genotypes. It is the main approach to sieve analysis because results can be interpreted in terms of vaccine efficacy, which has been the normative way to evaluate vaccine efficacy by pathogen strains (e.g., many vaccine efficacy trials over the past decades reported vaccine efficacy results separately by virus serotype). Because Approach B sieve analysis evaluates vaccine efficacy, it is conducted in the same cohort as the primary analysis of vaccine efficacy that did not account for virus genotype.

In contrast, Approach A sieve analyses only include primary endpoint HIV-1 acquisition endpoint cases in the analysis, comparing the genotype distribution between infected vaccine recipients vs. infected placebo recipients. As such, these results are not interpretable in terms of genotype-specific vaccine efficacy. However, they are useful supplemental analyses because they enable graphical descriptives and statistical inferences of whether and how genotype distributions differ between the two groups, where differences will be reflective either of differential vaccine efficacy by genotype and/or any post-infection vaccine effects that modified viral evolution between acquisition and the sampling of the viral sequences. It is very challenging to determine with certainty whether observed differences in genotype distributions are caused by differential vaccine efficacy or by post-infection impact, or by some combination of the two effects, such that Approach A results should be interpreted firstly as detection of some vaccine effect, and then additional considerations are needed to pursue a biological interpretation of the results.

The analyses of Approach B estimate and plot mark-specific vaccine efficacy (with 95% CIs). Additional implementation details for Approach B sieve analysis are described in Section 8.9.

8.1 Removing Unalignable Sites from the Sieve Analysis

Due to the variability of HIV-1's envelope, some sites, particularly in the hypervariable regions, are impossible to align in any way that will provide any confidence about functional equivalence. Such sites should not be included in the sieve analysis, as any variability at these sites would be noise, and including them into the analysis would reduce our statistical power.

These sites are located within the following positions: 133–154 (in V1), 185–192 with the exception of site 186 (in V2), 392–416 with the exception of site 392 (in V4), and 460–466 (in V5). These sites are summarized in Table 1.

However, while virtually impossible to align correctly due to the multiple occurrence of insertions and deletions at various times during the evolution of the viral population, nonetheless, these hypervariable regions present characteristics that are independent of the alignment and can be objectively measured for each Env in the alignment, namely: the density and number of the glycosylation sites, the length (number of AA of the region), and the net charge (computed cumulatively by assigning a positive charge to KRH and a negative to DE). Because these features, particularly in V1V2, can be associated with increased resistance to specific bNAbs , we plan on exploring whether in the selected V705 TF viruses we observe a difference in such characteristics between the vaccinees and the placebo group as detailed in Section 8.9.

Table 1: Sites to Exclude from Sieve Analyses Due to Unalignability

**Env Region**	**Hypervariable Sites Excluded**
V1	133 – 154
V2	185 – 192 (with the exception of 186)
V4	392 – 416 (with the exception of 392)
V5	460 – 466

Notes:

- Sites that are represented as a pair of adjacent positions (e.g., 408-409) include all insertions between the supplied HXB2 positions. This will not be relevant to those analyses that will not be looking at sites that are insertions relative to HXB2, for reasons pertaining to alignment-based reproducibility.
- The following sites in these variable loops were included in the sieve analyses for the following reasons (largely due to the effort made to preserve the alignment of sequons):
  - 186: N conserved
  - 392: N conserved

8.2 Structuring AA Sequence Features for Sieve Analysis

The AA sequence sieve analysis has a pre-specified hypothesis-driven primary analysis tier (Tier 1) and a pre-specified exploratory analysis tier (Tier 2) (see also Section 8.13 for analysis tiers).

8.2.1 Summary of All Analyzed Tier 1 and 2 AA Sequence Features and Associated Statistical Approaches

The following AA sequence sieve analyses are pre-specified in a treatment-blinded manner, with the analysis tier indicated:

1. [AA site-scanning: Tier 1 for hypothesis-driven Env sites; Tier 2 for all analyzed Env gp120 sites.]

   For each AA position with at least 4 cases having a reference-matched residue and at least 4 cases having a reference-mismatched residue regardless of the sequence support (henceforth referred to as the minimum variability filter), we will make inference about $VE_h(v)$ for a binary position-specific sequence feature V_{obs} defined as a reference residue match (= 1) vs. mismatch (= 0), where the reference sequence is defined as an Env vaccine sequence. The analysis is conducted for each of the three Env vaccine-insert sequences. For each insert/reference sequence, we will report for each AA position meeting the condition above a two-sided unadjusted p-value from the test of differential $VE_h(v)$ for the two levels v of the feature, as well as point and 95% confidence interval estimates for each of $VE_h(1)$ and $VE_h(0)$. In addition, for the Clade C Env boost C97ZA reference sequence, we will report FWER- and FDR-adjusted p-values (see Section 8.13 for method description), where all screened-in positions constitute a multiple comparison procedure. The multiplicity correction is performed separately by tier.

   The Tier 1 hypothesis-driven sites comprise the union of the following sets of specified sites, where all the sites will be analyzed with a single multiple testing correction. These sets of sites are based on:

   a [V1V2 peptide microarray hotspot results from the NHP challenge model provided by Janssen.]

   The peptide microarray yielded the following sites in the N-terminal side of V2 that were relevant for vaccine protection in the NHP model: sites 157–184. Any of these 28 sites that do not pass all of the filters (i.e., alignment difficulties/hypervariable or minimum variability filter) will be excluded.

   b [V1V2 IgG3 correlates of risk results.]

   A protein alignment of the V1V2 loop sequences from HXB2 and the nine V1V2 antigens used in the IgG3 correlates of risk analysis were generated by MAFFT

using the LANL HIValign tool available at https://www.hiv.lanl.gov. The aligned sequences were compared between the strongest inverse correlate of risk (gp70-001428.2.42) and the two gp70 antigens with an estimated HR closest to one (gp70-Ce1086_B2 and gp70-1394C9G1). All sites in the non-hypervariable regions of the V1V2 loop that had a mismatch to the reference antigen (gp70-001428.2.42) for either comparator antigen are included. These sites are: 130, 155, 160, 161, 164, 165, 166, 170, 172, 173, 178, 179, 181, 186, and 200. Note, many of these sites are also identified by the peptide microarray data noted above.

c [V2 C1 ADCC epitope sites.]

We will assess in Tier 1 signature sites previously found to be linked to differential vaccine efficacy in the RV144 vaccine study: ADCC epitope in the C1 conserved region (Pollara et al., 2014; Easterhoff et al., 2020) (sites listed in Table 2).

d [RV144 VE-signature sites.]

We will assess in Tier 1 signature sites previously found to be linked to differential vaccine efficacy in the RV144 vaccine study: positions 169 and 181 (Rolland* et al., 2012).

e [Signature sites for Clade C bnAbs.]

Several studies have shown that over time the genetic diversity acquired by HIV-1 has increased its resistance to both serum samples as well as broadly neutralizing antibodies (Stefic et al., 2019) (Rademeyer et al., 2016) (Hraber et al., 2014). Additionally, recent work conducted by Bette Korber and Elena Giorgi, motivated by conversations with Georgia Tomaras regarding the creation of a new virus panel, has led to the observation that bNAb epitopes are becoming more diverse over time, especially sites within the V2 apex epitope. Motivated by this phenomenon, we will look at specific sites that have been most impacted by changes in diversity in Env, and that, especially in the C clade, have been shown to increase viral resistance to bNAbs, including the V2 apex epitope, the CD4 binding site, MPER epitope, the fusion peptide epitope, and contact glycans shown to be linked to resistance to V3 bNAbs (Bricault et al., 2019). The set of sites are listed in Table 2.

2. [Potential N-linked glycosylation (PNG) sites that may be poorly covered by the vaccine inserts (Tier 1).]

   In Tier 1, we will examine notable PNG sites with prior evidence that they are poorly covered by the vaccine inserts. One such example are the PNG sites at positions 234 and 392, which are missing from the C-boost Env (Bette Korber CHAVD presentation, October 2021); these sites will be analyzed. In addition, V2 bnAb contact glycans at sites 130, 156, 168–173, and 185–187 will be assessed.

   For these analyses, we will be looking for the presence or absence of an N-linked glycosylation motif (a.k.a. “sequon”): [N][not P][S or T].

3. [Physico-chemical weighted Hamming distances: Tier 1 for hypothesis-driven sets of AA sites; Tier 2 for all analyzed sites.]

   Inference is conducted for $VE_h(v)$ for univariable V_{obs} varying over levels v of a physicochemical weighted Hamming distance to each reference Env vaccine sequence. Four Tier 1 physicochemical weighted Hamming distances will be analyzed: one defined using the V1V2-hotspot positions 157–184, one defined using the V2 correlates-generated positions, one defined using the V2 C1 ADCC epitope positions, and one defined using the signature sites for C-clade bnAbs (sites listed in Table 2).

   Two Tier 2 physico-chemical weighted Hamming distances will be analyzed: one defined the same as above except all filtered-in Env gp120 AA sites are included, and one defined based on positions in the V5 loop.

   For these distance-mark analyses, sites not passing the alignment difficulties/hypervariable filter will be excluded, but sites that pass these filters but do not pass the minimum-variability filter will be included.

4. [Other Env gp120 AA sequence features (Tier 1 or Tier 2).]

   Additional Tier 1 Env gp120 AA features that will be analyzed are: Length of V1V2, Total number of sequons in V1V2, and Electrochemical charge of V2.

   Additional Tier 2 Env gp120 AA features that will be analyzed are: Length of V5, Total number of sequons in V5.

   In calculating the above features (hypervariable sequence lengths, number of sequons, and electrochemical charge), we will include all sites, including those that were deemed unalignable, as each one of these characteristics is calculated for each sequence individually (and independently of the alignment).

5. [Indicator of whether a case's set of sequences is estimated to be multi-lineage vs. a single lineage (Tier 1).

   This Tier 1 feature is distinct from AA sequence features in being a measure of complexity of infection, not being about AA residue content.

Table 2 lists the set of marks V_{obs} that will be analyzed (all position numberings use the HXB2 numbering system).

8.3 Exploratory Tier 2 Coverage Analysis

To better understand the coverage provided by the C-clade boost as opposed to using a more central, mosaic insert, we will use the LANL tool EpiCover (Fischer et al, 2007; Turmond et al, 2008) to compare the coverage across all of Env gp120 achieved by a single mosaic insert, the C-clade boost alone, and the mosaic and C-clade used together.

Table 2: List of AA Sequence Features V_{obs} for Sieve Analysis

**Tier 1 Hypothesis-Driven**	
1. Env gp120 AA site-scanning	– a. V1V2-hotspot Janssen NHP challenge: sites 157–184<br>– b. IgG3 V1V2 breadth-correlates sites: 130, 155, 160, 161, 164, 165, 166, 170, 172, 173, 178, 179, 181, 186, 200<br>– c. RV144 V2 C1 region sites within ADCC epitope: 51–61, 69–78<br>– d. RV144 primary sieve sites: 169, 181 (Rolland, Edlefsen et al. 2012)<br>– e. Signature sites for C-clade bnAbs (i.e., VRC01, 4E10, 3BNC117, and CAP256.VRC26): 230, 279–282, 350, 364, 429, 432, 456, 471
2. Presence/absence of sequons	– Glycan holes in C97ZA insert: 234, 392 (Korber CHAVD presentation, Oct. 2021)<br>– V2 bnAb contact glycans: 130, 156, 160, 234, 332, 392
3. PC-weighted Hamming dist	– a. V1V2-hotspot Janssen NHP challenge: sites 157–184<br>– b. IgG3 V1V2 breadth-correlates sites: 130, 155, 160, 161, 164, 165, 166, 170, 172, 173, 178, 179, 181, 186, 200<br>– c. RV144 V2 C1 region sites within ADCC epitope: 51–61, 69–78<br>– d. Signature sites for C-clade bnAbs (i.e., VRC01, 4E10, 3BNC117, and CAP256.VRC26): sites 230, 234, 279–282, 350, 364, 429, 432, 456, 471.
4. Other Env AA features	– Length of V1V2<br>– Number of sequons in V1V2<br>– Electrochemical charge of V2
5. Multiplicity of viruses	– Single vs. multiple lineages
**Tier 2 Exploratory Analysis**	
1. Env gp120 AA site-scanning	– All sufficiently-variable positions in Env gp120
2. PC-weighted Hamming dist	– All of Env gp120<br>– All of V5
3. Other AA features	– Length of V5<br>– Number of sequons in V5

Notes: All of the features specified above are included in the May 21, 2022 reports. Only proportional-hazards based inferential methods are used for the May 21, 2022 reports. Descriptive plots are included comparing the distributions of features between the vaccine and placebo arms, but no inferences (i.e., confidence intervals, p-values) are done using post-infection sieve analysis methods.

8.4 Exploratory Tier 2 Analysis of Signature Sites for Clade C bnAbs

In order to measure whether there has been significant genetic drift over time at the "signature sites for Clade C bnAbs", we will use as "background sequences" Envs downloaded from the LANL database spanning a sampling time of 20 years, from 1998 to 2018 (where 2018 is chosen as an arbitrary time cut-off of sequences sampled earlier than the 705 Envs). We will compare the entropy at the specified sites and epitopes and investigate whether there have been a significant change in entropy in the HVTN 705 placebo arm Envs compared to the earlier ones sampled from the database, and, if present, we will investigate whether these changes have been toward more resistance or more sensitivity to one of the major bnAb classes (V2, V3, CD4bs or fusion peptide). The results may be illustrated using logo plots and structure visualizations. These analyses are exploratory, and are not sieve analyses per se that study potential vaccine effects.

8.5 Sieve Analysis Inference Conditioning on Infection (Approach A Methods)

For continuous/quantitative marks, the Approach A sieve analysis is conducted as described in Subsection 8.8. The Approach A methods are included for their potential to have improved statistical power. These inferential analyses will be accompanied by plots of the distribution of the mark by each of the two treatment arms.

8.6 Accounting for Multiplicity of Sequences from Participants Who Acquired HIV-1

The sieve analysis is based on a single sequence per participant, selected as a participant's closest-to-the-consensus sequence, based on the physicochemical (PC)-weighted Hamming distance in Env. When such sequence does not exist due to genetic diversity driven by either the presence of multiple, genetically distinct, lineages, or by immune pressure, the most represented sequence from the most numerous lineage is chosen instead.

More in detail, we proceed as follows. We begin by using a semi-automated algorithm to distinguish alignments with a single "lineage" (where by lineage we mean a viral population that evolved from a single founder virus) from those with multiple lineages based on the number of peaks of the inter-sequence Hamming distance frequency distribution. Typically in early infection, inter-sequence Hamming distances from a set of sequences all belonging to the same lineage follow a unimodal distribution (Keele et al., 2008). The exception to this are: (a) presence of APOBEC enrichment, which we correct by calculating an additional distribution obtained from masking "G" columns in the alignment that are in APOBEC context (i.e. the "GRD" motif), or (b) the presence of non-random accumulation of mutations due to early

host immune pressure. In general, we find that intersequence Hamming distance frequency distribution remains unimodal even in the presence of those first immune selection pressures, thus making for a good criterion to distinguish single vs. multiple lineage infection. Because some participants are sampled later and have a higher degree of diversity due to selection pressure, our automated algorithm is followed by visual inspection to confirm the original classification.

Once the single vs. multiple lineage classification is completed, we proceed to screen the multiple lineage infections for recombination using the LANL tool RAPR (Song et al., 2018). When found, recombinants are excluded from founder selection due to the fact that it is not always possible to discern whether the recombination event happened before or after infection (i.e. in the donor or the recipient).

The “mindist” algorithm mentioned earlier is then applied to the resulting alignments, single lineage and recombinant-free multiple lineage, as follows. For each alignment we calculate the “majority” consensus (namely, the most frequent nucleotide at each position) and then sort all sequences by their PC-weighted Hamming distance from this consensus. In single lineage infections, as well as multiple lineage infections where there is a clear “majority” lineage, the lowest ranked sequence will be identical to the consensus (i.e. it will be the sequence with a “0” Hamming distance), and this is the sequence selected for the sieve analyses.

In all other cases we take the lowest ranked sequence in the alignment (i.e. the one with the lowest PC-weighted Hamming distance from the consensus) and, if there is more than one, we resolve ambiguities by visual inspection of the sequences themselves and the quality control metrics provided by the sequencing labs.

Each step in this strategy is augmented with manual inspection by sequence experts, as the unsupervised algorithms may not handle recombination in an adequate way. This founder identification process will be conducted with all of the NT sequences for the full reads for each insert gene (gag, pol, env).

8.7 Application of Approach A and B Sieve Analyses to the AA Sequence Features in Tier 1 and Tier 2

Table 3 maps the AA sequence features for sieve analysis to which each Approach A and B sieve analysis methods are applied. The table refers to marks V_{obs} defined in Table 2 and the elaborated text in Section 8.2.

Table 3: Application of Approach A and B sieve analysis to the AA sequence features in Tier 1 and Tier 2[1,2]

Tier	Mark/Analysis (Marks V_{obs} defined in Table 2)
1, 2	AA residue match vs. mismatch (all binary; AA site-scanning)
	Tier 1 studies sites in Table 2
	Tier 2 studies Env sites in Table 2
	Approach B VE_h LM 1995, VE_c NPMLE AJ
	Approach A GenSig phylogenetically-corrected AA site
	signature analysis (LANL) for all specified sites (Bhattacharya et al. 2007 Science)[3]
1, 2	Physicochemical weighted Hamming distances
	Approach A DRTMLE
	Approach B VE_h JG 2013
	Approach B VE_h LM 1995
	Tier 1 studies the weighted Hamming distances based on sites in Table 2
	Tier 2 studies the weighted Hamming distance based on sites in Table 2
1	Indicator of multi-lineage infection
	Approach B VE_h LM 1995

[1] As noted above VE_h inferences are included for the May 21 report, VE_c NPMLE AJ inferences and Approach A inferences (DRTMLE, GenSig) are not included for the May 21 report. Results on the indicator of multi-lineage infection are included for the May 21 report.

[2]Nomenclature: NPMLE = Nonparametric maximum likelihood estimation; AJ = Aalen Johansen (1978) NPMLE for genotype-specific cumulative risk; DRTMLE = doubly-robust targeted minimum loss-based estimation; CRAN = Comprehensive R Archive Network.

[3]Approach A AA site-scanning sieve analysis will be conducted via phylogenetically corrected signatures analysis using the method (**?**), which uses the reconstructed phylogeny at each site to correct for association bias due to ancestral relatedness between sequences rather than phenotype. In this analysis, the probability of each base in each codon is calculated to reconstruct the most probable ancestral states of each codon. The most recent common ancestor of a given codon is compared to the codon in end point taxa to do the analysis. The output of the signature analysis are the translated codons, i.e. amino acids, but the trees used to obtain the phylogenetically corrected signatures are nucleotide based.

8.8 Approach A Conditional Sieve Analysis Statistical Method

The so-called Approach A method compares the marginal mean value of the AA sequence feature V_{obs} between the vaccine arm viruses and the placebo arm viruses. This approach will be applied using doubly robust targeted minimum loss-based estimation (DRTMLE) for

both quantitative and binary readouts (Benekser et al., 2017), to estimate the mean readout of each group with a Wald 95% confidence interval, as well as the mean difference (vaccine - placebo) with a Wald 95% confidence interval.

For analyses with any missing data on marks, the DRTMLE method will accommodate the missing data by including a model of the probability of observing the mark, where the model will be simple because there is only a small amount of missing data. Note that these conditional methods include all infected cases in the analysis, in contrast to the unconditional vaccine efficacy sieve analyses noted above that exclude cases with missing marks from the analysis.

In addition, all implementations of the DRTMLE method adjust for baseline covariates that are judged to possibly be associated with both HIV-1 infection and with the mark feature under analysis. Analyses adjust for the same baseline covariates adjusted for in the immune correlates analyses: South Africa vs. not South Africa, BMI, age, and baseline behavioral risk score. The baseline behavioral risk score was built by superlearner of the placebo arm data, which is the same risk score used for the HVTN 705 immune correlates analyses.

This covariate adjustment is needed because the analysis must control for all dual predictors of HIV-1 infection and the mark endpoint in order that the estimated contrast in means across treatment arms assesses a causal effect in the sub-population of participants who would be HIV-1 infected under either treatment assignment (vaccine or placebo) (e.g., Shepherd et al., 2006). More aggressive covariate adjustment is not done because adjusting for covariates predictive of HIV-1 infection but not the mark would reduce precision, and do not need to be accounted for as they are not confounders. In particular baseline risk score is not adjusted for as it could readily be highly correlated with infection but not with the mark feature.

The covariate adjustment is achieved using a simple superlearner model for the conditional outcome regression (with the mark the outcome). For quantitative marks, the non-negative least squares (NLLS) loss function (the default for the SuperLearner R package with a quantitative outcome) is used. In addition, 5-fold cross-validation is used, with the following learners for predicting a quantitative outcome (as hard-coded into the Superlearner R package): SL.mean, SL.glm, and SL.glmnet.All of these learners are documented in the SuperLearner R package, where SL.mean fits a sample mean, SL.glm fits a standard linear regression, and SL.glmnet fits a LASSO model for a quantitative outcome selecting the tuning parameter λ via cross validation such that error is within one standard error of the minimum.

For analyses with missing mark data a superlearner model with minus binomial log likelihood-loss is used to estimate the pobability of observing the mark, with 5-fold cross-validation. If fewer than 20% of cases are missing marks, then the library of learners is small, only including SL.mean and SL.glm (with logistic link). If between 20% and 40% of cases are missing marks, then the library is expanded to also include SL.step, SL.bayesglm (with logistic link), SL.glm (with logistic link), SL.glm.interaction (with logistic link), and SL.glmnet (with logistic link). If more than 40% of cases are missing marks, then the library is further expanded to include SL.xgboost and SL.ksvm (mote data adapti VE algorithms). For a

binary outcome such as the observed data indicator, SL.step performs a stepwise selection logistic regression optimizing AIC, SL.bayesglm fits a bayesian logistic regression, SL.glm fits a standard logistic regression, SL.gam fits a generalized additi VE model with 2 degrees of smoothing, SL.glm.interaction fits a logistic regression with all 2-way interactions and SL.glmnet fits a LASSO model for a binary outcome selecting λ via cross validation such that error is within one standard error of the minimum. The amount of missing mark data was less than 10%, such that only SL.mean and SL.glm are used.

In addition, Superlearner is also used to estimate the propensity score for treatment assignment to vaccine vs. placebo, using the mean model, logistic regression and lasso regression in the library of learners (SL.mean, SL.glm, SL.glmnet in the Superlearner R package), which use a logit link function given the outcome being predicted is binary. This superlearning uses minus binomial log likelihood-loss with 5-fold cross-validation. The same superlearner approach was used for the propensity score for neutralization sieve analysis in Corey et al. (2021). The covariates employed for this propensity score model are age, BMI, indicator of South Africa, and the baseline behavioral risk score.

The analysis will be done using the *drtmle* R package by Dr. David Benkeser available at CRAN. The code accomplishes the following two tasks for reporting of results for a mark V_{obs}:

1. Report point and 95% confidence interval estimates of the mean V_{obs} for each of the vaccine and placebo arms.
2. Report point and 95% confidence interval estimates of the mean difference in V_{obs} (vaccine - placebo arm)

8.9 Details of Implementation of Approach B Proportional Hazards Sieve Analysis

For each analysis, the result is reported as point and 95% confidence interval estimates of vaccine efficacy as a function of the mark V_{obs} with figures portraying the results.

The semiparametric proportional hazards estimation and hypothesis testing methods of Juraska and Gilbert (2013) are applied. First, the two-sided Wald test of $\{H_{10} : VE(v) = 0 \text{ for all } v\}$ is applied. Second, the two-sided Wald test of $\{H_{20} : VE(v) = VE \text{ for all } v\}$ is applied: the Wald test statistic (Juraska and Gilbert, 2013, Section 3) for the mark coefficient in the density ratio model is used to test H_{20} against $\{H_{21} : VE(v) \text{ varies with } v\}$.

8.10 Analysis of Indicator of Multi-Lineage Infection

Bar plots will describe the proportion of primary endpoint acquisition events that are multi-lineage infections by vaccine and placebo arm. In addition, the proportional-hazards differ-

ential VE sieve analysis method will be used to assess whether VE differs against single- vs. multi-lineage infection. This analysis will not be included in any multitesting procedure.

8.10.1 Handling Non-Subtype C Acquisition Primary Endpoints

Almost 100% of primary endpoints have acquired viruses that are of subtype C. Only two primary endpoints are a non-C subtype virus (A1/C recombinants). Given very few non-C viruses, all viruses are included in all sieve analyses, and the subtype is not accounted for. Essentially the entire analysis can be interpreted for C subtype viruses.

8.11 Descriptive Analysis of Amino Acid Sequence Features V_{obs}

For each sequence feature that is included in sieve analyses, the distribution of the mark will be plotted for primary endpoint HIV-1 infected cases, stratified by vaccine vs. placebo group.

8.12 Phylogenetic Trees

Phylogenetic trees will be constructed and plotted, with annotation by country and vaccine/placebo group. The trees will contain:

- The representative sequence selected from each participant
- All insert sequences
- The HXB2 reference sequence
- The current subtype C consensus sequence available from the LANL HIV Sequence Database

Additionally, a second tree will be made to confirm clade assignments, and it will contain the HXB2 reference sequence as well as the A-J clade reference sequences downloaded from the LANL database. All trees will be created using FastTree v2. NT trees will be created using the GTR model with Gamma distribution, whereas AA trees will be built using the JTT+CAT model.

8.13 Multiple Hypothesis Testing Adjustment for Sieve Effects

Westfall and Young (1993) p-values are used to estimate family-wise error rate (FWER) adjusted p-values and FDR-adjusted p-values, used for each set of analyses listed below,

done separately for Tier 1 and Tier 2 analyses, with Tier 1 and Tier 2 specified here. The Westfall and Young function is implemented as in Dr. Youyi Fong's R package *kyotil*.

For the Env gp120 sieve analysis, multiple testing correction is performed for each of two genotype categories of sieve analysis separately. The first category is amino acid site-scanning including glycan analysis (feature types 1 and 2 for Tier 1 in Table 2; feature type 1 for Tier 2 in Table 2). The second category is physico-chemical Hamming distances and other AA sequence features (feature types 3 and 4 for Tier 1 in Table 2; feature types 2 and 3 for Tier 2 in Table 2). For each category cross-classified with tier, FWER- and FDR-adjusted p-values are computed for all of the sieve effect tests that are conducted based on VE_h. This means that for the May 21, 2022 interim sieve analysis, a total four separate multiplicity adjustments are performed for the Clade C C97ZA boost Env reference sequence: (1) Tier 1 feature types 1 and 2; (2) Tier 1 feature types 3 and 4; (3) Tier 2 feature type 1; (4) Tier 2 feature types 2 and 3.

Tier 1 feature type 5 (single vs. multiple lineages) will be a stand-alone analysis, not included in any multiplicity adjustment.

The multiplicity adjustment is performed for the Clade C Env boost C97ZA reference sequence. The analyses of the other two vaccine-insert Envs are sensitivity analyses, where unadjusted p-values are included for comparison with the primary analysis results based on the Clade C C97ZA insert.

Definition of statistical significance in the context of multiple hypothesis testing. In general, unadjusted p-values, FWER-adjusted p-values, and FDR-adjusted p-values are reported. FWER statistical significance is defined by FWER p-value ≤ 0.05, whereas FDR statistical significance is defined by FDR p-value ≤ 0.20 and unadjusted p-value ≤ 0.05. All tests are two-sided except sieve tests involving a Hamming distance, which are one-sided. For multiplicity sets in which one-sided testing is performed, the FWER and FDR statistical significance is applied to doubled one-sided p-values.

9 R Code for the Sieve Analysis Methods

The planned sieve analysis methods are implemented in R and are publicly available. The following table summarizes the R code that is used.

Table 4: R code for implementing the sieve analyses*

Method	R code
Lunn and McNeil (1995) mark-specific proportional hazards model Approach B sieve analysis for a binary/trinary mark	Fred Hutch internal code
Juraska and Gilbert (2013) mark-specific hazard-ratio model Approach B sieve analysis for a quantitative mark	R package *sievePH* at CRAN
Benkeser et al. (2019) NPMLE Aalen-Johansen (AJ) cumulative incidence based VE Approach B sieve analysis for a $K = 2 - 4$ category mark	R package *survtmle* at CRAN
Benkeser et al. (2017) DRTMLE Approach A sieve analysis for a quantitative mark	R package *drtmle* at CRAN

*Nomenclature: NPMLE = Nonparametric maximum likelihood estimation; AJ = Aalen Johansen (1978) NPMLE for genotype-specific cumulative risk; DRTMLE = doubly-robust targeted minimum loss-based estimation; CRAN = Comprehensive R Archive Network.

10 Additions to the v1 SAP: New Sieve Analyses Implemented in the v2 SAP

10.1 Additional Env Sequence Features

- The v1 SAP restricted to gp120; now all of Env is analyzed, including the signal peptide. The extension to the full Env applies to the site-scanning and Hamming distance analyses. For the latter, a new gp41-specific physico-chemical weighted Hamming distance to each vaccine insert will be analyzed, restricted to the subregion of gp41 included in the vaccine inserts. All PC-weighted Hamming distances analyzed under v2 SAP are listed in Table 5.

- The v1 SAP conducted amino acid site scanning analysis in terms of matched vs. mismatched character compared to the vaccine-strain-character at a given amino acid position. The v2 SAP adds a reference-independent version of amino acid site scanning that conducts Approach B sieve analysis for every genotype defined by a specific character at a given amino acid position, compared to the complement of the genotype (all other characters combined). In addition, all amino acid site scanning in Tier 2 excludes the Tier 1 hypothesis-driven position set to avoid duplicating sieve tests for the same binary feature in Tier 1 and 2.

  - The vaccine residue match/mismatch site scanning will be done for the region of

Env included in the vaccine inserts.

  - The reference-independent residue presence/absence site scanning will be done for all of Env including the signal peptide. For the vaccine-insert region, the analysis will assess differential VE for each sufficiently prevalent non-vaccine-insert residue vs. the complement of that residue. Vaccine-insert residues will be ignored in the vaccine-insert region to avoid duplication (these residues are evaluated in the vaccine residue match/mismatch analysis in the previous bullet). For the non-vaccine-insert region, we will assess differential VE for each sufficiently prevalent residue vs. the complement of that residue.

- The v2 SAP adds two physico-chemical weighted Hamming distances to each vaccine insert sequence for two sets of sites identified in the HVTN 505 sieve study (deCamp et al., 2017):

  1. 93 CD4bs antibody contact sites
  2. 54 AA sites corresponding to 9-mers in 4 linear regions overlapping the CD4bs that correlated with vaccine efficacy in HVTN 505

- The v2 SAP adds the cysteine count in gp160. Note that the cysteine counts are only of interest when studying all of gp160 since the sieve effect signal in HVTN 505 was focused in the transmembrane domain.

- The v2 SAP includes, for each of the V1V2 positions with HXB2 coordinates 130, 155, 160, 161, 164, 165, 166, 170, 172, 173, 178, 179, 181, 186, and 200, a binary indicator mark defined as residue match/mismatch to the gp70-001428.2.42 (henceforth referred to as 1428) V1V2 antigen sequence. These marks are motivated by the result from the HVTN 705 immune correlates analysis where IgG3 against the 1428 V1V2 had evidence as an inverse correlate of HIV-1 acquisition risk. The listed AA positions are those exhibiting a residue mismatch between the strongest V1V2 IgG3 CoR and either of the two weakest V1V2 IgG3 CoRs among all gp70-scaffolded V1V2 antigens.

- The v2 SAP includes the PC-weighted Hamming distance to the 1428 V1V2 antigen sequence in the subset of positions listed in the previous bullet that are alignable.

10.2 Complete List of Env Sequence Features for v2 SAP

Table 5 provides a complete list of Env sequence features for inclusion in the sieve analysis implementing v2 SAP.

10.3 Multiple Hypothesis Testing Adjustment for v2 SAP

Under version 2.0, this section replaces Section 8.13. The only change in this section is the expansion of the set of Env sequence features as detailed in Table 5.

To minimize the occurrence of false positive sieve effect results given the large number of analyzed sequence features (see Table 5) and thereby increase the credibility of the sieve analysis, it is critical to use a stringent multiplicity adjustment for sieve tests based on VE_h. To this end, Westfall and Young (1993) p-values are used to estimate family-wise error rate (FWER) adjusted p-values and FDR-adjusted p-values, applied separately to the following four multiplicity sets of features, characterized by feature types and analysis tiers as listed in Table 5:

- feature types 1.–4. in Tier 1;
- feature types 5.–7. in Tier 1;
- feature types 1.–2. in Tier 2;
- feature types 3.–4. in Tier 2,

where, for vaccine insert-dependent features, only those using the Clade C C97ZA boost Env reference sequence are included in the sets above. No adjusted p-values will be computed for sieve tests for Hamming distances from each the Ad26 Mos1 and Mos2S Env vaccine inserts; the analyses of Hamming distances from either of these two vaccine-insert Envs are sensitivity analyses, where unadjusted p-values are included for comparison with the primary analysis Hamming distance results based on the Clade C C97ZA insert Env.

The Westfall and Young function is implemented as in Dr. Youyi Fong's R package *kyotil* available on CRAN.

Tier 1 feature type 8 (single vs. multiple lineages) will be a stand-alone analysis, not included in any multiplicity adjustment.

Definition of statistical significance in the context of multiple hypothesis testing. In general, unadjusted p-values, FWER-adjusted p-values, and FDR-adjusted p-values are reported. FWER statistical significance is defined by FWER p-value ≤ 0.05, whereas FDR statistical significance is defined by FDR p-value ≤ 0.20 and unadjusted p-value ≤ 0.05. All tests are two-sided except sieve tests involving a Hamming distance, which are one-sided. For multiplicity sets in which one-sided sieve testing is performed, the FWER and FDR statistical significance is applied to doubled one-sided p-values.

Table 5: Complete list of AA sequence features V_{obs} studied in v2 SAP, with additions in v2 shown in blue

**Tier 1 Hypothesis-Driven**	
1. Env gp120 vaccine residue match/mismatch site-scanning	– a. V1V2-hotspot Janssen NHP challenge: sites 157–184<br>– b. IgG3 V1V2 breadth-correlates sites: 130, 155, 160, 161, 164, 165, 166, 170, 172, 173, 178, 179, 181, 186, 200<br>– c. RV144 V2 C1 region sites within ADCC epitope: 51–61, 69–78<br>– d. RV144 primary sieve sites: 169, 181 (Rolland, Edlefsen et al. 2012)<br>– e. Signature sites for C-clade bnAbs (i.e., VRC01, 4E10, 3BNC117, and CAP256.VRC26): 230, 279–282, 350, 364, 429, 432, 456, 471
2. Env gp70-1428 V1V2 antigen sequence residue match/mismatch	– a. IgG3 V1V2 breadth correlates sites: 130, 155, 160, 161, 164, 165, 166, 170, 172, 173, 178, 179, 181, 186, 200
3. Env gp120 C97ZA vaccine insert-independent residue presence/absence site-scanning	– a. V1V2-hotspot Janssen NHP challenge: sites 157–184<br>– b. IgG3 V1V2 breadth-correlates sites: 130, 155, 160, 161, 164, 165, 166, 170, 172, 173, 178, 179, 181, 186, 200<br>– c. RV144 V2 C1 region sites within ADCC epitope: 51–61, 69–78<br>– d. RV144 primary sieve sites: 169, 181 (Rolland, Edlefsen et al. 2012)<br>– e. Signature sites for C-clade bnAbs (i.e., VRC01, 4E10, 3BNC117, and CAP256.VRC26): 230, 279–282, 350, 364, 429, 432, 456, 471
4. Presence/absence of sequons	– Glycan holes in C97ZA insert: 234, 392 (Korber CHAVD presentation, Oct. 2021)<br>– V2 bnAb contact glycans: 130, 156, 160, 234, 332, 362, 363, 392
5. PC-weighted Hamming dist from vaccine insert	– a. V1V2-hotspot Janssen NHP challenge: sites 157–184<br>– b. IgG3 V1V2 breadth correlates sites: 130, 155, 160, 161, 164, 165, 166, 170, 172, 173, 178, 179, 181, 186, 200<br>– c. RV144 V2 C1 region sites within ADCC epitope: 51–61, 69–78<br>– d. Signature sites for C-clade bnAbs (i.e., VRC01, 4E10, 3BNC117, and CAP256.VRC26): sites 230, 234, 279–282, 350, 364, 429, 432, 456, 471.<br>– e. HVTN 505 sieve analysis: 93 CD4bs mAb contact sites<br>– f. HVTN 505 sieve analysis: 54 sites corresponding to 9-mers in 4 linear regions overlapping the CD4bs contact residues
6. PC-weighted Hamming dist from gp70-1428 V1V2 antigen	– a. IgG3 V1V2 breadth correlates sites: 130, 155, 160, 161, 164, 165, 166, 170, 172, 173, 178, 179, 181, 186, 200
7. Other Env AA features	– Length of V1V2<br>– Number of sequons in V1V2<br>– Electrochemical charge of V2<br>– Cysteine count in gp160
8. Multiplicity of virus lineages	– Single vs. multiple lineages
**Tier 2 Exploratory Analysis**	
1. Vaccine residue match/mismatch site-scanning in vaccine-insert region of Env excluding Tier 1 hypothesis-driven position set	– All sufficiently-variable positions in signal peptide + gp120 + N-terminal portion of gp41 included in vaccine inserts excluding Tier 1 hypothesis-driven positions to avoid duplication
2. Vaccine insert-independent residue presence/absence site-scanning in all of Env excluding Tier 1 hypothesis-driven position set	– All sufficiently-variable positions in Env excluding Tier 1 hypothesis-driven positions to avoid duplication (vaccine insert residues are ignored in the vaccine-insert region)
3. PC-weighted Hamming dist to vaccine insert	– All of gp120<br>– N-terminal portion of gp41 included in vaccine inserts<br>– All of V5
4. Other AA features	– Length of V5<br>– Number of sequons in V5 (dichotomized as < 2 vs. ≥ 2)

11 Additions to the v2 SAP: New Sieve Analyses Implemented in the v3 SAP

11.1 Analysis of Predicted Env Structures for Functional Sieve Effects

We will use AlphaFold 3 (AF3) to predict Env trimer structures for all Env sequences used in the sieve analysis as well as the vaccine insert. Specifically, we used the gp120 (HXB2 31–511) and gp41 (HXB2 512–665) regions of the Env sequences as input due to both prior works creating reliable multimer structures in AlphaFold (Bai et al., 2024) and our work here with the creation of high-predictive scoring Env trimer structures in AF3. The top AF3 scored model from 5 predictions was used for all following analysis.

To analyze structural differences from the vaccine insert and identify and corroborate potential sieve effects, a structural similarity metric was used, the template modeling score (TM-Score) (Zhang and Skolnick, 2004), for comparing each predicted structure of the primary endpoints to the insert of a single protomer chain ('A'). The TM-score was selected due to its length-independent nature, necessary for certain comparisons when handling sets of proteins with non-uniform lengths. AF3 also utilizes the TM-Score to measure prediction accuracies (Abramson et al., 2024) and continues to be used for the evaluation of models throughout the literature (Wee and Wei, 2024).

Although TM-scores are often utilized to compare entire proteins, the gp140 region of sequences in the trial are relatively homogeneous and would show little to no TM-score signals. Instead, we utilized linear N-mers to calculate TM-score chunks to get a finer level of detail and structural identity within a protein. We calculated TM-scores of each 15-mer window across gp120 with an overlap/step size of 4 positions between all primary endpoints and the vaccine insert. Of note, a 15-mer is defined as 15 linear HXB2 alignment positions rather than 15 linear AAs in the structure. This is done to ensure we are comparing the same structural region within each window. As such, a floor of 6 AAs within a window was set to ensure differences not be dominated by point mutations. We tested the impact of higher floors (i.e. 10 AA) and found similar results while generally increasing similarities. No significant positive correlation was found between shortening of the N-mers and signal strength, but rather a negative correlation was, suggesting longer N-mers were in fact more signal determinative. Thus, selecting a shorter floor was optimal to avoid the removal of potentially significant windows based on length. Finally, a site-level metric was obtained by attributing each TM-score from a 15-mer window to its associated HXB2 alignment sites. This results in 1-4 scores per site which were all considered as possible features in the new sieve analysis.

11.2 Sieve Analysis of High-Dimensional Signals in Amino Acid Sequence Features

If there is FDR-significant evidence of any sieve effects from the Approach B sieve analysis, we will apply the DiProPerm (direction-projection-permutation, a.k.a. "DPP") test to look for signals distinguishing vaccine vs. placebo as a function of input variables V_{obs}. DPP is a two-sample hypothesis test for comparing two high-dimensional distributions, and is exact—i.e., the type I error is guaranteed to be controlled at the nominal level for any n—making it suitable for datasets of high dimensionality and low sample size.

We will use DPP to calculate the p-value for each set of input variables, plus determine the variable importance (i.e., the loadings) for each variable in each set. All variable sets will adjust for age, BMI, baseline risk score, and enrollment in South Africa (i.e., Set 1 below).

For comparison, the p-value for the null model with no input features will also be reported. Due to limitations of the DPP software, this null data will consist of two features, each with an equal split of two different values (1 and 2) across the two treatment assignments.

The individual sets and subsets of features to be analyzed are outlined in the list below, starting with just the baseline variables and looking at individual groups of Tier 1 and Tier 2 features (matching those found in **Table 5**) and some additional structure-related marks.

1. Control/baseline variables only
   - Age, BMI, baseline risk score and enrollment in South Africa
2. Tier 1: Env gp120 C97ZA insert residue match/mismatch status
   (a) V1V2-hotspot Janssen NHP challenge: sites 157–184
   (b) IgG3 V1V2 breadth-correlates sites: sites 130, 155, 160, 161, 164, 165, 166, 170, 172, 173, 178, 179, 181, 186, 200
   (c) RV144 V2 C1 region sites within ADCC epitope: sites 51–61, 69–78
   (d) RV144 primary sieve sites (Rolland, Edlefsen et al. 2012): sites 169 and 181
   (e) Signature sites for C-clade bnAbs (i.e., VRC01, 4E10, 3BNC117, and CAP256.VRC26): sites 230, 279–282, 350, 364, 429, 432, 456, 471
3. Tier 1: Env gp70-1428 V1V2 antigen sequence residue match/mismatch status
   - IgG3 V1V2 breadth correlates sites: sites 130, 155, 160, 161, 164, 165, 166, 170, 172, 173, 178, 179, 181, 186, 200
4. Tier 1: Env gp120 C97ZA vaccine insert-independent residue presence/absence indicators
   (a) V1V2-hotspot Janssen NHP challenge: sites 157–184

  (b) IgG3 V1V2 breadth-correlates sites: sites 130, 155, 160, 161, 164, 165, 166, 170, 172, 173, 178, 179, 181, 186, 200
  (c) RV144 V2 C1 region sites within ADCC epitope: sites 51–61, 69–78
  (d) RV144 primary sieve sites (Rolland, Edlefsen et al. 2012): sites 169 and 181
  (e) Signature sites for C-clade bnAbs (i.e., VRC01, 4E10, 3BNC117, and CAP256.VRC26): sites 230, 279–282, 350, 364, 429, 432, 456, 471

5. Tier 1: Presence/absence of sequons
   (a) Glycan holes in C97ZA insert (Korber CHAVD presentation, Oct. 2021): sites 234, 392
   (b) V2 bnAb contact glycans: sites 130, 156, 160, 234, 332, 362, 363, 392

6. Tier 1: PC-weighted Hamming distance from vaccine insert
   (a) V1V2-hotspot Janssen NHP challenge: sites 157–184
   (b) IgG3 V1V2 breadth correlates sites: sites 130, 155, 160, 161, 164, 165, 166, 170, 172, 173, 178, 179, 181, 186, 200
   (c) RV144 V2 C1 region sites within ADCC epitope: sites 51–61 and 69–78
   (d) Signature sites for C-clade bnAbs (i.e., VRC01, 4E10, 3BNC117, and CAP256.VRC26): sites 230, 234, 279–282, 350, 364, 429, 432, 456, 471
   (e) HVTN 505 sieve analysis (DeCamp et al.): 93 CD4bs mAb contact sites
   (f) HVTN 505 sieve analysis (DeCamp et al.): 54 sites corresponding to 9-mers in 4 linear regions overlapping the CD4bs contact residues

7. Tier 1: PC-weighted Hamming dist from gp70-1428 V1V2 antigen
   - IgG3 V1V2 breadth correlates sites: sites 130, 155, 160, 161, 164, 165, 166, 170, 172, 173, 178, 179, 181, 186, 200

8. Tier 1: Other Env AA features
   (a) Length of V1V2
   (b) Number of sequons in V1V2
   (c) Electrochemical charge of V2
   (d) Cysteine count in gp160
   (e) Indicators of single vs multiple viral lineages

9. Tier 2: Vaccine residue match/mismatch indicators in vaccine-insert region of Env, excluding Tier 1 hypothesis-driven position set

- All sufficiently-variable positions in signal peptide + gp120 + N-terminal portion of gp41 included in vaccine inserts, excluding Tier 1 hypothesis-driven positions to avoid duplication

10. Tier 2: Vaccine insert-independent residue presence/absence indicators in all of Env, excluding Tier 1 hypothesis-driven position set

    - All sufficiently-variable positions in Env, excluding Tier 1 hypothesis-driven positions to avoid duplication (vaccine insert residues are ignored in the vaccine-insert region)

11. Tier 2: PC-weighted Hamming distance to the vaccine insert

    (a) All of gp120

    (b) N-terminal portion of gp41 included in vaccine inserts

    (c) All of V5

12. Tier 2: Other AA features

    (a) Length of V5

    (b) Number of sequons in V5 (dichotomized as < 2 vs. ≥ 2)

13. Structural Features: TM score, comparing sequence with C97ZA insert

    (a) All 15mer window TM scores

    (b) C1 region

    (c) V1/V2 loop

    (d) Loop D

    (e) C2 region

    (f) C3 region

    (g) C4 region

    (h) C5 region

    (i) Items (b) through (h) combined

    (j) β2 strand (from the N-terminal V1/V2 stem): positions 121-123

    (k) β3 strand (from the C-terminal V1/V2 stem): residues 201-203

    (l) β20 strand (from the N-terminal C4 region): residues 421-423

    (m) β21 strand (from the C-terminal C4 region): residues 433-435

    (n) Items (j) through (m) combined

    (o) Items (i) and (n) combined

    (p) All of gp120

For each major feature set above (sets 1 through 13), the variable subsets will be analyzed individually and combined. (E.g., for Set 8, there will be separate individual analyses for Sets 8a through 8e, and a combined analysis that will include all Sets 8a-8e combined.)

All analyses will be controlled for the baseline variables in Set 1.

For sets 2-12, we will conduct additional combined analyses to determine if the structural features contribute to the signal with any of the non-structural feature sets. This will be done by running the analysis with the original set with set 13o, which will contain all of the structural features except all of gp120 (set 13p). E.g., for Set 10, we will run the original analysis (Set 10 combined with Set 1) and the combined structural analysis (Sets 10, 1 and 13o combined).

An additional analysis of **All Features** will be conducted on all sets combined (feature sets 1 through 13).

As mentioned, this analysis will only be conducted if any FDR-significant results are found in the final Approach B sieve analysis or in any planned T cell epitope sieve analyses. As such, this machine learning analysis will not be conducted for the May 21, 2022 report.

12 Revision from SAP v3.0 to v4.0

SAP v4.0 is created in response to peer-review feedback concerning the use of one-sided tests for sieve analyses involving physicochemical (PC)-weighted Hamming distances. SAP v3.0 prespecified that all tests were two-sided except sieve tests involving a Hamming distance, which were one-sided, and that multiplicity adjustment for these tests was applied to doubled one-sided p-values (see Section 10.3).

Although this approach was prespecified before treatment unblinding, SAP v4.0 revises the testing strategy so that all reported sieve test p-values are based on two-sided tests. Specifically, the Approach B proportional hazards sieve analysis method for quantitative marks, including PC-weighted Hamming distances, is updated to use the two-sided Wald test for testing whether $VE(v)$ varies with v. The multiple-testing adjustment procedures are unchanged, except that they now use the corresponding two-sided p-values for all Env sequence features.

References

Aalen, O. (1978), "Nonparametric inference for a family of counting processes." *Annals of Statistics*, 6, 701–726.

Abramson, J., Adler, J., Dunger, J., Evans, R., Green, T., Pritzel, A., Ronneberger, O., Willmore, L., Ballard, A. J., Bambrick, J., Bodenstein, S. W., Evans, D. A., Hung, C.-W., O'Neill, M., Reiman, D., Tunyasuvunakool, K., Wu, Z., Žemgulytė, A., Arvaniti, E., et al. (2024), "Accurate structure prediction of biomolecular interactions with AlphaFold 3," *Nature*, 630, 493–500.

Bai, H., Lewitus, E., Li, Y., Thomas, P. V., Zemil, M., Merbah, M., Peterson, C. E., Thuraisamy, T., Rees, P. A., Hajduczki, A., Dussupt, V., Slike, B., Mendez-Rivera, L., Schmid, A., Kavusak, E., Rao, M., Smith, G., Frey, J., Sims, A., Wieczorek, L., Polonis, V., Krebs, S. J., Ake, J. A., Vasan, S., Bolton, D. L., Joyce, M. G., Townsley, S., and Rolland, M. (2024), "Contemporary HIV-1 consensus Env with AI-assisted redesigned hypervariable loops promote antibody binding," *Nature Communications*, 15, 3924.

Benkeser, D., Carone, M., Laan, M. V. D., and Gilbert, P. (2017), "Doubly robust nonparametric inference on the average treatment effect," *Biometrika*, 104, 863–880.

Benkeser, D., Gilbert, P. B., and Carone, M. (2019), "Estimating and Testing Vaccine Sieve Effects Using Machine Learning," *Journal of the American Statistical Association*, 1–12.

Benkeser, D., Juraska, M., and Gilbert, P. B. (2020), "Assessing Trends in Vaccine Efficacy by Pathogen Genetic Distance." *Societe Francaise de Statistique*, 161.

Bricault, C. A., Yusim, K., Seaman, M. S., Yoon, H., Theiler, J., Giorgi, E. E., Wagh, K., Theiler, M., Hraber, P., Macke, J. P., et al. (2019), "HIV-1 neutralizing antibody signatures and application to epitope-targeted vaccine design," *Cell host & microbe*, 25, 59–72.

Corey, L., Gilbert, P., Juraska, M., Montefiori, D., Morris, L., Karuna, S., Edupuganti, S., and et al. (2021), "Two randomized trials of neutralizing antibodies to prevent HIV-1 acquisition," *New England Journal of Medicine*.

deCamp, A., Rolland, M., Edlefsen, P., Sanders-Buell, E., Hall, B., Magaret, C., Fiore-Gartland, A., Juraska, M., Carpp, L., Karuna, S., Bose, M., LePore, S., Miller, S., O'Sullivan, A., Poltavee, K., Bai, H., Dommaraju, K., Zhao, H., Wong, K., Chen, L., Ahmed, H., Gottardo, R., Koup, R., Bailer, R., Mascola, J., Graham, B., Roederer, M., O'Connell, R., Michael, N., Robb, M., Adams, E., D'Souza, P., Kublin, J., Corey, L., Geraghty, D., Frahm, N., Tomaras, G., McElrath, M., Frenkel, L., Strychak, S., Tovanabutra, S., Sobieszczyk, M., Hammer, S., Kim, J., Mullins, J., and Gilbert, P. B. (2017), "Sieve analysis of breakthrough HIV-1 sequences in HVTN 505 identifies vaccine pressure targeting the CD4 binding site of Env-gp120," *PLoS One*, 12, e0185959, pMCID: PMC5693417.

Easterhoff, D., Pollara, J., Luo, K., Janus, B., Gohain, N., Williams, L. D., Tay, M. Z., Monroe, A., Peachman, K., Choe, M., Min, S., Lusso, P., Zhang, P., Go, E. P., Desaire, H., Bonsignori, M., Hwang, K.-K., Beck, C., Kakalis, M., O'Connell, R. J., Vasan, S., Kim, J. H., Michael, N. L., Excler, J.-L., Robb, M. L., Rerks-Ngarm, S., Kaewkungwal, J., Pitisuttithum, P., Nitayaphan, S., Sinangil, F., Tartaglia, J., Phogat, S., Wiehe, K., Saunders, K. O., Montefiori, D. C., Tomaras, G. D., Moody, M. A., Arthos, J., Rao, M., Joyce, M. G., Ofek, G., Ferrari, G., and Haynes, B. F. (2020), "HIV vaccine delayed boosting increases Env variable region 2-specific antibody effector functions," *JCI Insight*, 5.

Fischer, W., Perkins, S., Theiler, J., Bhattacharya, T., Yusim, K., Funkhouser, R., Kuiken, C., Haynes, B., Letvin, N. L., Walker, B. D., Hahn, B. H., and Korber, B. T. (2007), "Polyvalent vaccines for optimal coverage of potential T-cell epitopes in global HIV-1 variants," *Nat. Med.*, 13, 100–106.

Gilbert, P. B. (2000), "Comparison of competing risks failure time methods and time-independent methods for assessing strain variations in vaccine protection." *Statistics in Medicine*, 19, 3065–3086.

Gilbert, P. B., Self, S. G., and Ashby, M. A. (1998), "Statistical methods for assessing differential vaccine protection against human immunodeficiency virus types," *Biometrics*, 799–814.

Gilbert, P. B. and Sun, Y. (2015), "Inferences on relative failure rates in stratified mark-specific proportional hazards models with missing marks, with application to HIV vaccine efficacy trials." *Journal of the Royal Statistical Society: Series C (Applied Statistics)*, 64, 49–73, pMCID: PMC4310507.

Grambsch, P. and Therneau, T. (1994), "Proportional hazards tests and diagnostics based on weighted residuals." *Biometrika*, 81, 515–526.

Heng, F., Sun, Y., and Gilbert, P. (2022), "Estimation and hypothesis testing of strain-specific vaccine efficacy with missing strain types, with applications to a COVID-19 vaccine trial," *arXiv*, 2201.08946.

Hraber, P., Korber, B. T., Lapedes, A. S., Bailer, R. T., Seaman, M. S., Gao, H., Greene, K. M., McCutchan, F., Williamson, C., Kim, J. H., Tovanabutra, S., Hahn, B. H., Swanstrom, R., Thomson, M. M., Gao, F., Harris, L., Giorgi, E., Hengartner, N., Bhattacharya, T., Mascola, J. R., and Montefiori, D. C. (2014), "Impact of clade, geography, and age of the epidemic on HIV-1 neutralization by antibodies," *J. Virol.*, 88, 12623–12643.

Juraska, M. and Gilbert, P. B. (2013), "Mark-specific hazard ratio model with multivariate continuous marks: An application to vaccine efficacy," *Biometrics*, 69, 328–337, pMCID: PMC3940058.

— (2016), "Mark-specific hazard ratio model with missing multivariate marks." *Lifetime Data Analysis*, 22, 606–625, pMCID: PMC4848257.

Keele, B., Giorgi, E., Salazar-Gonzalez, J., and et al. (2008), "Identification and characterization of transmitted and early founder virus envelopes in primary HIV-1 infection." *Proceedings of the National Academy of Sciences*, 105, 7552–7557, pMCID: PMC2387184.

Lunn, M. and McNeil, D. (1995), "Applying Cox regression to competing risks." *Biometrics*, 51, 524–532.

Pollara, J., Bonsignori, M., Moody, M. A., Liu, P., Alam, S. M., Hwang, K.-K., Gurley, T. C., Kozink, D. M., Armand, L. C., Marshall, D. J., Whitesides, J. F., Kaewkungwal, J., Nitayaphan, S., Pitisuttithum, P., Rerks-Ngarm, S., Robb, M. L., O'Connell, R. J., Kim, J. H., Michael, N. L., Montefiori, D. C., Tomaras, G. D., Liao, H.-X., Haynes, B. F., and Ferrari, G. (2014), "HIV-1 vaccine-induced C1 and V2 Env-specific antibodies synergize for increased antiviral activities," *J. Virol.*, 88, 7715–7726.

Rademeyer, C., Korber, B., Seaman, M. S., Giorgi, E. E., Thebus, R., Robles, A., Sheward, D. J., Wagh, K., Garrity, J., Carey, B. R., Gao, H., Greene, K. M., Tang, H., Bandawe, G. P., Marais, J. C., Diphoko, T. E., Hraber, P., Tumba, N., Moore, P. L., Gray, G. E., Kublin, J., McElrath, M. J., Vermeulen, M., Middelkoop, K., Bekker, L.-G., Hoelscher, M., Maboko, L., Makhema, J., Robb, M. L., Abdool Karim, S., Abdool Karim, Q., Kim, J. H., Hahn, B. H., Gao, F., Swanstrom, R., Morris, L., Montefiori, D. C., and Williamson, C. (2016), "Features of recently transmitted HIV-1 clade C viruses that impact antibody recognition: Implications for active and passive immunization," *PLoS Pathog.*, 12, e1005742.

Rolland*, M., Edlefsen*, P., Larsen, B., and et al. (2012), "Increased HIV-1 vaccine efficacy against viruses with genetic signatures in Env V2." *Nature*, 490, 417–420, *Contributed equally.

Shepherd, B., Gilbert, P. B., Jemiai, Y., and Rotnitzky, A. (2006), "Sensitivity analyses comparing outcomes only existing in a subset selected post-randomization, conditional on covariates, with application to HIV vaccine trials." *Biometrics*, 62, 332–342.

Song, H., Giorgi, E. E., Ganusov, V. V., Cai, F., Athreya, G., Yoon, H., Carja, O., Hora, B., Hraber, P., Romero-Severson, E., Jiang, C., Li, X., Wang, S., Li, H., Salazar-Gonzalez, J. F., Salazar, M. G., Goonetilleke, N., Keele, B. F., Montefiori, D. C., Cohen, M. S., Shaw, G. M., Hahn, B. H., McMichael, A. J., Haynes, B. F., Korber, B., Bhattacharya, T., and Gao, F. (2018), "Tracking HIV-1 recombination to resolve its contribution to HIV-1 evolution in natural infection," *Nat. Commun.*, 9, 1928.

Stefic, K., Bouvin-Pley, M., Essat, A., Visdeloup, C., Moreau, A., Goujard, C., Chaix, M.-L., Braibant, M., Meyer, L., and Barin, F. (2019), "Sensitivity to broadly neutralizing antibodies of recently transmitted HIV-1 clade CRF02_AG viruses with a focus on evolution over time," *J. Virol.*, 93.

Sun, Y., Gilbert, P. B., and McKeague, I. (2009), "Proportional hazards models with continuous marks." *Annals of Statistics*, 37, 394–426, pMCID: PMC2762218.

Thurmond, J., Yoon, H., Kuiken, C., Yusim, K., Perkins, S., Theiler, J., Bhattacharya, T., Korber, B., and Fischer, W. (2008), "Web-based design and evaluation of T-cell vaccine candidates," *Bioinformatics*, 24, 1639–1640.

Wee, J. and Wei, G.-W. (2024), "Evaluation of AlphaFold 3's Protein–Protein Complexes for Predicting Binding Free Energy Changes upon Mutation," *Journal of Chemical Information and Modeling*, 64, 6676–6683.

Westfall, P. H., Young, S. S., et al. (1993), *Resampling-based multiple testing: Examples and methods for p-value adjustment*, vol. 279, John Wiley & Sons.

Zhang, Y. and Skolnick, J. (2004), "Scoring function for automated assessment of protein structure template quality," *Proteins: Structure, Function, and Bioinformatics*, 57, 702–710.
