# Supplementary material for: Quantifying how HIV-1 envelope sequence features impact vaccine efficacy in the HVTN 705/HPX2008 randomised trial in southern African women
Source: eBioMedicine. 2026 Aug 31;131:106459. doi: 10.1016/j.ebiom.2026.106459 (PMC13560528; doi:10.1016/j.ebiom.2026.106459)
Supplement: Supplementary file 2 [file mmc1.docx]

# **Supplementary Appendix for:**

# **Quantifying how HIV-1 envelope sequence features impact vaccine efficacy in the HVTN 705/HPX2008 randomised trial in southern African women**

**by Juraska, Li et al.**

**Figure S1. Methodology flow chart.** The diagram shows flow from HIV-1 acquisition primary endpoints in the per-protocol (PP) cohort through viral sequence processing, intra-host virus population characterisation, single-sequence and multi-sequence sieve analyses, feature-specific and quasispecies-level vaccine efficacy (VE) estimation, differential VE testing, multiple testing adjustment, and interpretation. AA, amino acid; BMI, body mass index; FDR, false discovery rate; FWER, familywise error rate; PC, physico-chemical; PNGS, potential N-linked glycosylation site; SMRT-UMI, single molecule real-time unique molecular identifier.

^1^ Westfall DH, Deng W, Pankow A, Murrell H, Chen L, Zhao H, et al. Optimized SMRT-UMI protocol produces highly accurate sequence datasets from diverse populations-Application to HIV-1 quasispecies. Virus Evol. 2024;10(1):veae019.

^2^ Juraska M, Gilbert PB. Mark-specific hazard ratio model with multivariate continuous marks: an application to vaccine efficacy. Biometrics. 2013;69(2):328-37.

^3^ Peng J, Juraska M, Shaw PA, Gilbert PB. Vaccine sieve analysis on deep sequencing data using competing risks Cox regression with failure type subject to misclassification [Preprint]. arXiv. arXiv:2512.09262 [stat.ME] Posted 10 Dec, 2025. Access date 12 Dec, 2025.

^4^ Lunn M, McNeil D. Applying Cox regression to competing risks. Biometrics. 1995;51(2):524-32.

^5^ Westfall PH, Young SS. p Value Adjustments for Multiple Tests in Multivariate Binomial Models. Journal of the American Statistical Association. 1989;84(407):780-6; Westfall PH, Young SS. Resampling-based multiple testing: Examples and methods for p-value adjustment: John Wiley & Sons; 1993.

**Table S1**. **Hypervariable *Env* AA positions**

| ***Env* Region** | Hypervariable AA Positions |
| --- | --- |
| V1 | 133–154 |
| V2 | 185–192 except 186 |
| V4 | 392–416 except 392 |
| V5 | 460–466 |

**Table S2. Feature subsets analysed by the DiProPerm method.** The number of variables included in each subset is reported in parentheses.

| **1. Control variables only** | |
| --- | --- |
| Subset 1 | Age, BMI, baseline risk score and enrolment in South Africa; these features were analysed independently as Subset 1, but also included in all other subsets to control for these features (r = 4) |
|  | |
| **2. Tier 1: Env gp120 C97ZA vaccine insert residue match/mismatch status (r = 85)** | |
| Subset 2a | V1V2-hotspot Janssen NHP challenge: positions 157–184 (r = 35) |
| Subset 2b | IgG3 V1V2 breadth-correlates positions: positions 130, 155, 160, 161, 164, 165, 166, 170, 172, 173, 178, 179, 181, 186, 200 (r = 19) |
| Subset 2c | RV144 V2 C1 region positions within ADCC epitope: positions 51–61, 69–78 (r = 26) |
| Subset 2d | RV144 primary sieve positions (Rolland, Edlefsen et al. 2012)^1^: positions 169 and 181 (r = 6) |
| Subset 2e | Signature positions for C-clade bnAbs (i.e., VRC01, 4E10, 3BNC117, and CAP256.VRC26): positions 230, 279–282, 350, 364, 429, 432, 456, 471 (r = 15) |
|  | |
| **3. Tier 1: Env gp70-1428 V1V2 antigen sequence residue match/mismatch status** | |
| Subset 3 | gG3 V1V2 breadth correlates positions: positions 130, 155, 160, 161, 164, 165, 166, 170, 172, 173, 178, 179, 181, 186, 200 (r = 19) |
|  | |
| **4. Tier 1: Env gp120 vaccine insert-independent residue presence/absence indicators (r = 432)** | |
| Subset 4a | V1V2-hotspot Janssen NHP challenge: positions 157–184 (r = 174) |
| Subset 4b | IgG3 V1V2 breadth-correlates positions: positions 130, 155, 160, 161, 164, 165, 166, 170, 172, 173, 178, 179, 181, 186, 200 (r = 131) |
| Subset 4c | RV144 V2 C1 region positions within ADCC epitope: positions 51–61, 69-78 (r = 44) |
| Subset 4d | RV144 primary sieve positions (Rolland, Edlefsen et al. 2012)^1^: positions 169 and 181 (r = 19) |
| Subset 4e | Signature positions for C-clade bnAbs (i.e., VRC01, 4E10, 3BNC117, and CAP256.VRC26): positions 230, 279–282, 350, 364, 429, 432, 456, 471 (r = 80) |
|  | |
| **5. Tier 1: PNG motif presence/absence (r = 14)** | |
| Subset 5a | Glycan holes in C97ZA insert (Korber CHAVD presentation, Oct. 2021): positions 234, 392 (r = 6) |
| Subset 5b | V2 bnAb contact glycans: positions 130, 156, 160, 234, 332, 362, 363, 392 (r = 12) |
|  |  |
| **6. Tier 1: PC-weighted Hamming distance from C97ZA vaccine sequence (r = 10)** | |
| Subset 6a | V1V2-hotspot Janssen NHP challenge: positions 157–184 (r = 5) |
| Subset 6b | IgG3 V1V2 breadth correlates positions: positions 130, 155, 160, 161, 164, 165, 166, 170, 172, 173, 178, 179, 181, 186, 200 (r = 5) |
| Subset 6c | RV144 V2 C1 region positions within ADCC epitope: positions 51–61 and 69-78 (r = 5) |
| Subset 6d | Signature positions for C-clade bnAbs (i.e., VRC01, 4E10, 3BNC117, and CAP256.VRC26): sites 230, 234, 279–282, 350, 364, 429, 432, 456, 471 (r = 5) |
| Subset 6e | HVTN 505 sieve analysis (deCamp et al.)^2^: 93 CD4bs mAb contact sites (r = 5) |
| Subset 6f | HVTN 505 sieve analysis (deCamp et al.)^2^: 54 positions corresponding to 9-mers in 4 linear regions overlapping the CD4bs contact residues (r = 5) |
|  | |
| **7. Tier 1: PC-weighted Hamming distance from gp70-1428 V1V2 antigen sequence** | |
| Subset 7 | IgG3 V1V2 breadth correlates sites: sites 130, 155, 160, 161, 164, 165, 166, 170, 172, 173, 178, 179, 181, 186, 200 (r = 5) |
|  | |
| **8. Tier 1: Other Env AA features (r = 9)** | |
| Subset 8a | Length of V1V2 (r = 5) |
| Subset 8b | Number of PNGS motifs in V1V2 (r = 5) |
| Subset 8c | Electrochemical charge of V2 (r = 5) |
| Subset 8d | Cysteine count in gp160 (r = 5) |
| Subset 8e | Indicator of single vs. multiple viral lineages (r = 5) |
|  | |
| **9. Tier 2: C97ZA vaccine residue match/mismatch in vaccine-insert region of Env, excluding Tier 1 hypothesis-driven position set** | |
| Subset 9 | All sufficiently-variable positions in signal peptide + gp120 + N-terminal portion of gp41 included in vaccine inserts, excluding Tier 1 hypothesis-driven positions to avoid duplication (r = 317) |
|  | |
| **10. Tier 2: Vaccine insert-independent residue presence/absence indicators in all of Env, excluding Tier 1 hypothesis-driven position set** | |
| Subset 10 | All sufficiently-variable positions in Env, excluding Tier 1 hypothesis-driven positions to avoid duplication (vaccine insert residues are ignored in the vaccine-insert region) (r = 1201) |
|  | |
| **11. Tier 2: PC-weighted Hamming distance to the C97ZA vaccine insert (r = 7)** | |
| Subset 11a | All of gp120 (r = 5) |
| Subset 11b | N-terminal portion of gp41 included in vaccine inserts (r = 5) |
| Subset 11c | All of V5 (r = 5) |
|  | |
| **12. Tier 2: Other AA features (r = 6)** | |
| Subset 12a | Length of V5 (r = 5) |
| Subset 12b | Number of PNGS motifs in V5 (dichotomised as < 2 vs. >= 2) (r = 5) |
|  | |
| **13. Structural features: TM score, comparing a participant’s mindist sequence with C97ZA vaccine sequence** | |
| Subset 13a | All 15mer window TM scores (r = 119) |
| Subset 13b | C1 region (r = 5) |
| Subset 13c | V1/V2 loop (r = 5) |
| Subset 13d | Loop D (r = 5) |
| Subset 13e | C2 region (r = 5) |
| Subset 13f | C3 region (r = 5) |
| Subset 13g | C4 region (r = 5) |
| Subset 13h | C5 region (r = 5) |
| Subset 13i | Subsets 13b through 13h combined (r = 11) |
| Subset 13j | Beta2 strand (from the N-terminal V1/V2 stem): positions 121–123 (r = 5) |
| Subset 13k | Beta3 strand (from the C-terminal V1/V2 stem): positions 201–203 (r = 5) |
| Subset 13l | Beta20 strand (from the N-terminal C4 region): positions 421–423 (r = 5) |
| Subset 13m | Beta21 strand (from the C-terminal C4 region): positions 433–435 (r = 5) |
| Subset 13n | Subsets 13j through 13m combined (r = 8) |
| Subset 13o | Subsets 13i and 13n combined (r = 15) |
| Subset 13p | All of gp120 (r = 5) |
|  | |
| **All features combined** | |
| Subset 14 | Subsets 1, 2a, 2b, 2c, 2d, 2e, 3, 4a, 4b, 4c, 4d, 4e, 5a, 5b, 6a, 6b, 6c, 6d, 6e, 6f, 7, 8a, 8b, 8c, 8d, 8e, 9, 10, 11a, 11b, 11c, 12a, 12b, 13a, 13o and 13p (r = 2,193) |

**Table S3. Feature multiplicity sets for adjustment of sieve test P-values**

| **Multiplicity Set** | **Feature Types** | **Number of Screened-in Features** |
| --- | --- | --- |
| *Single-sequence sieve analyses* | | |
| A | Tier 1 residue presence/absence  Tier 1 PNGS motif presence/absence | 138 6 |
| B | Tier 1 PC-weighted Hamming distances  Tier 1 hypervariable region characteristics  Tier 1 cysteine count | 7 3 1 |
| C | Tier 2 residue presence/absence | 1118 |
| D | Tier 2 PC-weighted Hamming distances  Tier 2 hypervariable region characteristics | 3 2 |
| E | Tier 2 15-mer TM-scores | 115 |
| *Multi-sequence sieve analyses* | | |
| F | Tier 1 residue presence/absence  Tier 1 PNGS motif presence/absence | 0 0 |
| G | Tier 2 residue presence/absence | 114 |

**Figure S2. Midpoint-rooted phylogenetic tree of 116 HIV-1 Env mindist sequences of primary endpoint cases.** Tips are colour-coded by subtype, and symbol shape and fill differentiate among treatment assignments and vaccine inserts. The tree was created using the IQ-Tree software with gamma distribution and ultra-fast bootstrapping for branch support.

**Figure S3. Midpoint-rooted phylogenetic tree of 116 HIV-1 Env mindist sequences of primary endpoint cases.** Tips are colour-coded by country, and symbol shape and fill differentiate among treatment assignments and vaccine inserts. The tree was created using the IQ-Tree software with gamma distribution and ultra-fast bootstrapping for branch support.

**Figure S4. Intra-host mean pairwise Hamming distance (HD).** Top panels: mean pairwise HD per nucleotide (NT), by study group, in single (left) vs. multiple (right) lineage primary endpoints. Bottom panels: rate of mean pairwise HD per nucleotide per day since the last RNA-negative sample collection date, by study group, in single (left) vs. multiple (right) lineage primary endpoints. P-values are from two-sided permutation tests using median quantile regression models adjusted for age, BMI, baseline risk score, and the indicator of South Africa vs. a different country. Violin plots were generated using the vioplot function.^3^

**Figure S5. Sections of inter-participant mindist alternate alignments of Env amino acid sequences that encompass amino acid position 364.** Reference sequences plus the participant mindist sequences with length variation in this region are shown. HXB2 amino acid position 364 corresponds to position 606 in this alternate alignment (designated with the red arrow).

**Table S4. Screened-in tier 2 residue presence/absence features in Env. Amino acid positions are numbered using HXB2 coordinates.**

**1118 screened-in residue presence/absence features**

2.K, 2.R, 4.K, 4.M, 4.R, 4.T, 5.E, 5.G, 6.I, 6.M, 6.T, 7.L, 7.M, 7.Q, 7.R, 7.W, 8.K, 9.N, 9.S, 10.C, 10.W, 10.Y, 11.L, 11.P, 11.Q12.H, 12.L, 12.P, 12.Q, 12.R, 13.L, 13.W, 13a.gap, 14.gap, 16.I, 16.M, 16.T, 18.G, 18.S, 19.I, 19.S, 21.A, 21.G, 22.F, 22.L, 23.W, 24.I, 24.L, 24.M, 25.F, 25.I, 25.L, 25.V, 25.gap, 26.I, 26.L, 26.M, 26.gap, 27.I, 27.M, 27.N, 27.S, 27.T, 27.V, 27.gap, 28.C, 28.S, 28.Y, 29.K, 29.N, 29.R, 29.S, 29a.gap, 30.G, 30.V, 30.gap, 31.E, 31.G, 31.I, 31.K, 31.L, 31.M, 31.R, 31.T, 31.V, 31.gap, 32.E, 32.G, 32.K, 32.N, 32.R, 33.D, 33.E, 33.K, 33.N, 33.Q, 33.gap, 33a.gap, 34.L, 34.M, 34.R, 34.S, 46.K, 46.R, 46.T, 47.D, 49.K, 50.A, 62.D, 62.E, 62.V, 63.K, 63.Q, 63.R, 63.T, 65.A, 65.V, 68.I, 80.D, 80.N, 80.S, 82.Q, 84.I, 84.L, 84.M, 84.V, 85.E, 85.F, 85.I, 85.K, 85.L, 85.P, 85.V, 86.L, 86.M, 87.A, 87.E, 87.G, 87.K, 87.gap, 89.V, 92.N, 95.M, 97.E, 97.K, 99.D, 99.N, 99.Y, 101.A, 102.D, 102.E, 105.H, 105.Q, 106.E, 108.I, 110.N, 110.S, 111.I, 113.D, 113.E, 114.E, 117.K, 121.K, 122.L, 128.T, 132.I, 132.K, 132.N, 132.S, 132.T, 156.N, 184d.gap, 184e.gap, 184f.gap, 184g.gap, 184h.gap, 184i.gap, 184j.S, 184j.gap, 184k.N, 184k.S, 184k.gap, 184l.D, 184l.N, 184l.gap, 184m.D, 184m.E, 184m.G, 184m.gap, 184n.D, 184n.E, 184n.G, 184n.K, 184n.N, 184n.S, 184n.gap, 184o.D, 184o.E, 184o.G, 184o.K, 184o.N, 184o.S, 184o.gap, 184p.D, 184p.E, 184p.G, 184p.K, 184p.N, 184p.S, 184p.T, 184p.gap, 184q.D, 184q.E, 184q.G, 184q.K, 184q.N, 184q.S, 184q.T, 184q.gap, 194.I, 195.H, 195.N, 195.S, 198.A, 198.S, 198.T, 199.S, 199.T, 201.I, 201.V, 202.A, 202.K, 202.T, 208.I, 209.N, 209.S, 209.T, 210.F, 211.D, 211.E, 215.I, 217.F, 219.A, 223.F, 227.K, 227.R, 229.K, 229.N, 231.E, 231.K, 231.N, 232.T, 236.K, 236.S, 236.T, 238.L, 238.P, 240.H, 240.K, 240.N, 240.Q, 240.S, 240.T, 244.T, 252.K, 252.R, 255.I, 265.L, 266.A, 266.S, 266.T, 267.E, 267.K, 268.E, 268.G, 268.K, 268.gap, 269.D, 269.E, 269.G, 269.K, 270.I, 270.T, 270.V, 271.I, 271.V, 272.I, 273.K, 273.R, 273.S, 274.S, 275.E, 275.K, 276.N, 277.I, 277.L, 277.M, 278.S, 278.T, 283.I, 283.T, 286.A, 286.V, 287.H, 287.Q, 288.F, 289.K, 289.N, 290.D, 290.E, 290.K, 290.N, 290.Q, 291.A, 291.P, 291.S, 291.T, 292.I, 292.V, 293.E, 293.G, 293.K, 293.N, 293.P, 293.Q, 293.S, 293.T, 295.E, 295.I, 295.N, 295.T, 295.V, 297.A, 297.E, 297.I, 297.T, 297.V, 299.P, 300.G, 300.N, 300.S, 303.T, 305.K, 305.Q, 305.R, 305.T, 306.G, 306.S, 307.I, 307.M, 307.V, 308.G, 308.R, 316.A, 316.T, 316.V, 317.F, 318.F, 318.Y, 318a.A, 318a.gap, 319.T, 319.gap, 320.G, 320.N, 321.A, 321.D, 321.E, 321.G, 321.N, 322.I, 322.V, 323.I, 325.D, 325.N, 326.I, 328.A, 328.E, 328.K, 328.Q, 328.R, 330.H, 330.Y, 332.K, 332.N, 332.S, 332.T, 333.I, 333.V, 334.N, 334.S, 334.T, 335.A, 335.E, 335.G, 335.K, 335.R, 336.A, 336.D, 336.E, 336.G, 336.K, 336.N, 336.Q, 336.R, 336.S, 336.T, 336.gap, 337.A, 337.D, 337.E, 337.G, 337.K, 337.N, 337.Q, 337.R, 337.gap, 339.E, 339.I, 339.N, 339.T, 340.D, 340.E, 340.K, 340.N, 340.R, 340.T, 341.A, 341.T, 342.I, 342.L, 342.V, 343.E, 343.H, 343.K, 343.L, 343.Q, 343.R, 343.Y, 344.D, 344.E, 344.G, 344.K, 344.L, 344.M, 344.N, 344.Q, 344.R, 345.I, 345.V, 346.A, 346.G, 346.K, 346.R, 346.S, 347.D, 347.E, 347.K, 347.N, 347.R, 348.E, 348.K, 351.E, 351.G, 351.K, 351.N, 352.H, 352.K, 352.L, 352.Y, 353.F, 353.L, 353.Y, 354.H, 354.K, 354.N, 354.P, 354.gap, 355.gap, 356.G, 356.K, 356.N, 357.K, 357.R, 357.T, 358.I, 358.N, 358.S, 358.T, 359.I, 359.T, 359a.I, 360.E, 360.I, 360.K, 360.N, 360.Q, 360.R, 360.T, 360.V, 362.A, 362.E, 362.G, 362.K, 362.N, 362.Q, 362.S, 362a.gap, 363.N, 363.P, 363.Q, 363.S, 365.A, 365.S, 369.L, 369.P, 371.I, 371.V, 372.T, 372.V, 373.M, 373.T, 375.S, 375.T, 377.N, 379.G, 379.R, 386.D, 386.N, 388.S, 388.T, 389.D, 389.E, 389.G, 389.I, 389.K, 389.N, 389.Q, 389.R, 389.T, 390.I, 392.D, 392.K, 392.N, 392.S, 392.T, 417.L, 417.P, 417.Q, 419.K, 419.R, 421.K, 423.F, 423.I, 424.I, 425.N, 425.R, 426.L, 426.M, 434.I, 434.M, 439.I, 440.A, 440.E, 442.E, 442.K, 442.N, 442.S, 444.T, 446.E, 446.I, 446.K, 446.N, 446.R, 446.T, 446.V, 448.N, 448.S, 452.I, 453.I, 455.E, 455.T, 455.V, 459.G, 459d.gap, 459e.N, 459e.gap, 459f.N, 459f.gap, 459g.N, 459g.gap, 459h.N, 459h.gap, 459i.G, 459i.K, 459i.N, 459i.R, 459i.T, 459i.gap, 459j.D, 459j.G, 459j.N, 459j.S, 459j.T, 459j.gap, 467.I, 467.T, 474.D, 474.N, 476.K, 477.D, 477.N, 481.N, 489.I, 491.I, 491.V, 492.E, 492.K, 492.N, 492.Q, 492.R, 496.I, 496.L, 496.V, 500.D, 500.E, 500.G, 500.K, 500.R, 500.T, 501.A, 502.K, 502.R, 504.Q, 504.R, 507.E, 507.G, 507.K, 507.Q, 507a.gap, 507b.gap, 509.E, 509.G, 509.Q, 510.K, 513.A, 513.V, 513.gap, 514.G, 514.T, 515.I, 515.L, 515.M, 515.V, 518.L, 518.M, 518.V, 519.F, 519.I, 519.L, 520.F, 520.L, 524.G, 525.A, 525.V, 535.I, 535.L, 535.M, 535.V, 536.A, 536.T, 539.A, 539.V, 543.Q, 546.S, 553.N, 553.S, 557.K, 557.R, 565.L, 565.M, 577.Q, 577.R, 578.A, 583.I, 583.L, 583.M, 583.V, 588.K, 588.Q, 588.R, 591.Q, 595.I, 595.L, 601.K, 602.I, 602.L, 605.T, 607.A, 607.N, 607.S, 607.T, 609.P, 612.A, 612.L, 612.N, 612.S, 612.T, 613.S, 613.T, 616.N, 617.K, 617.R, 618.S, 618.T, 619.E, 619.K, 619.L, 619.Q, 619.V, 619.Y, 620.A, 620.D, 620.E, 620.G, 620.K, 620.N, 620.S, 620.T, 621.D, 621.E, 621.N, 621.Y, 624.D, 624.E, 624.G, 624.N, 624.Q, 626.L, 626.M, 629.I, 629.M, 630.E, 630.Q, 632.D, 633.K, 633.R, 635.I, 636.D, 636.N, 636.S, 639.S, 640.D, 640.E, 640.G, 640.N, 640.Q, 640.S, 640.Y, 641.I, 641.L, 641.T, 644.E, 644.G, 644.K, 644.M, 644.N, 644.Q, 644.R, 644.S, 644.W, 647.E, 647.G, 648.D, 648.E, 648.K, 648.V, 649.S, 651.I, 651.K, 651.N, 651.S, 651.T, 655.E, 655.I, 655.K, 655.N, 655.Q, 655.R, 658.K, 658.Q, 658.R, 659.D, 659.E, 662.A, 662.E, 662.S, 664.D, 664.N, 665.K, 665.S, 667.A, 667.D, 667.K, 667.N, 667.Q, 668.N, 668.S, 671.N, 671.S, 671.T, 674.D, 674.N, 674.S, 676.S, 676.T, 677.H, 677.K, 677.N, 677.Q, 677.R, 683.K, 683.R, 684.I, 684.L, 687.I, 687.M, 688.I, 688.V, 689.I, 689.V, 698.I, 698.V, 699.F, 699.L, 700.A, 700.G, 700.T, 701.V, 702.I, 702.L, 704.I, 704.L, 704.V, 705.I, 705.V, 706.K, 706.N, 706.R, 707.K, 707.R, 708.V, 710.Q, 717.F, 717.L, 720.L, 720.P, 721.I, 721.L, 721.P, 721.T, 723.A, 723.N, 723.S, 723.T, 724.P, 724.Q, 724.S, 725.G, 725.R, 726.E, 726.G, 727.L, 727.P, 730.L, 730.P, 731.E, 731.G, 731.R, 732.E, 732.G, 732.R, 734.E, 736.E, 736.G, 740.Q, 741.D, 741.G, 741.N, 742.K, 742.N, 742.R, 742.S, 743.D, 743.E, 743.G, 743.N, 743.S, 744.K, 744.R, 746.I, 746.T, 746.V, 750.N, 750.S, 753.F, 753.L, 754.A, 754.P, 754.S, 754.T, 755.I, 755.L, 756.A, 756.F, 756.I, 756.V, 758.D, 758.E, 762.N, 762.S, 765.L, 767.C, 767.L, 767.S, 769.H, 769.R, 770.H, 770.L, 770.Q, 770.R, 773.D, 773.N, 774.C, 774.F, 774.L, 775.I, 775.L, 775.T, 776.L, 776.S, 777.I, 777.L, 777.V, 778.A, 778.I, 778.T, 778.V, 779.A, 779.T, 779.V, 781.A, 781.G, 781.T, 781.V, 782.A, 782.V, 783.E, 784.I, 784.L, 784.R, 786.G, 787.H, 787.R, 787.gap, 787a.N, 787a.S, 787a.gap, 787b.I, 787b.S, 787b.gap, 787e.L, 787e.gap, 787f.K, 787f.Q, 787f.R, 787f.gap, 787g.G, 787h.I, 787h.L, 787h.gap, 787i.Q, 787i.gap, 788.K, 788.R, 789.G, 789.V, 791.E, 792.A, 792.I, 792.T, 792.V, 795.H, 795.Y, 797.G, 797.K, 798.G, 798.N, 798.S, 799.I, 799.L, 800.A, 800.G, 800.I, 800.L, 800.V, 801.L, 801.Q, 802.Y, 803.W, 804.C, 804.G, 804.V, 805.L, 805.Q, 807.I, 807.L, 808.K, 809.K, 809.R, 810.S, 812.I, 812.T, 812.V, 813.N, 813.S, 815.F, 815.L, 815.V, 817.A, 817.S, 817.T, 818.I, 818.L, 818.T, 818.V, 820.I, 821.A, 821.I, 821.R, 821.T, 821.V, 822.T, 822.V, 823.A, 823.G, 824.E, 829.I, 829.V, 830.I, 830.L, 831.A, 831.E, 832.A, 832.F, 832.G, 832.I, 832.L, 832.V, 833.A, 833.I, 833.L, 833.V, 834.L, 834.Q, 834.R, 835.G, 835.R, 836.F, 836.I, 836.L, 836.V, 837.C, 837.F, 837.G, 837.Y, 839.A, 839.G, 840.F, 840.I, 841.C, 841.L, 841.R, 841.Y, 842.H, 842.N, 843.I, 843.L, 843.V, 844.P, 845.R, 845.T, 845.gap, 846.R, 846.gap, 847.I, 847.V, 847.gap, 848.R, 849.Q, 850.G, 851.A, 851.F, 851.L, 851.gap, 852.E, 853.A, 853.I, 853.S, 853.T, 853.gap, 854.A, 854.S, 854.gap, 855.L, 856.L, 856.Q, 856.gap

**Figure S6. Difference in natural log hazard ratio (vaccine/placebo) of the primary endpoint (residue X-specific HIV diagnosis minus residue not X specific HIV diagnosis) vs. unadjusted two-sided P-value (Lunn-McNeil test^4^) that tests for differential VE, calculated for all screened-in tier 2 residue presence/absence features in Env (n=1118 screened-in features).**

**Fig. S7. Vaccine efficacy (VE) against viruses with specific PNGS amino acid site status for screened-in Env amino acid positions for glycans associated with increased resistance to V2, V3, and CD4bs bnAbs.** Filled squares designate VE point estimates, and bars extend through the 95% confidence intervals (CIs). Two-sided p-values are from two-sided Wald tests. Two-sided differential VE p-values were obtained using the Lunn-McNeil test.^4^ Positions annotated with an asterisk (*) represent glycan holes in the C97ZA insert sequence.

**Table S5.** **Amino acid position sets pertaining to PC-weighted Hamming distances. Positions are numbered using HXB2 coordinates.**

| **Hypothesis*** | **Env amino acid position set** |
| --- | --- |
| V2-associated protection in NHP challenge model | 157–184 |
| IgG3 V1V2 correlate in HVTN 705 | 130, 155, 160, 161, 164–166, 170, 172, 173, 178, 179, 181, 186, 200 |
| C1 epitope of ADCC correlate of risk in RV144 | 51–61, 69–78 |
| Subtype C CD4bs-, V3-, and MPER-bnAb resistance-associated signatures | 230, 279–282, 350, 364, 429, 432, 456, 471 |
| Signature CD4bs antibody footprints in HVTN 505 | 49, 94, 96–99, 102, 105, 108, 109, 112, 122–129, 195–199, 210, 255–257, 275, 276, 278–283, 353, 354, 356, 357, 360, 362, 364–373, 375, 376, 382, 384, 386, 417–419, 421, 424–432, 455–467, 469–480 |
| CD4bs-overlapping signature k-mers in HVTN 505 | 192–207, 365–373, 425–437, 464–479 |

* Hypotheses described in Methods and Table 1.

**Figure S8.** **Vaccine efficacy (VE) against viruses with specific physicochemical (PC)-weighted Hamming distance from the C97ZA Env vaccine sequence in clade C bnAb resistance-associated signature positions excluding position 364.** The horizontal box plots at the top show distributions of the PC-weighted Hamming distance in primary endpoint cases in the vaccine group (open red triangles, n=53) and the placebo group (open blue circles, n=63), where the vertical line in each boxplot represents the median PC-weighted Hamming distance and the boxes extend through the 25^th^ and 75^th^ percentiles. “Unadjusted Sieve P” is a two-sided p-value from testing (Wald test) whether VE varies with the sequence distance on the x-axis.

**Figure S9.** **Vaccine efficacy (VE) against viruses with specific physicochemical (PC)-weighted Hamming distance from the Mos1 (A and C) and Mos2S (B and D) Env vaccine sequence in clade C bnAb resistance-associated signature positions including 364 (A and B) and excluding position 364 (C and D).** The dashed black curves represent 95% pointwise confidence intervals. The horizontal box plots at the top show distributions of the PC-weighted Hamming distances in primary endpoint cases in the vaccine group (open red triangles, n=53) and the placebo group (open blue circles, n=63), where the vertical line in each boxplot represents the median PC-weighted Hamming distance and the boxes extend through the 25^th^ and 75^th^ percentiles. “Unadjusted Sieve P” is a p-value from testing (Wald test) whether VE varies with the sequence distance on the x-axis.

**Figure S10. Shannon entropy for the set of clade C bnAb resistance signature positions in the vaccine and placebo groups, and a set of matched reference sequences selected from the LANL HIV Sequence database.** The vertical dashed segments are 95% bootstrap confidence intervals. n=94 LANL reference sequences, n=63 HVTN 705 placebo sequences, n=53 HVTN 705 vaccine sequences).

**Figure S11**. **Vaccine efficacy (VE) against viruses with specific physicochemical (PC)-weighted Hamming distance from the Mos1 (A) and Mos2S (B) Env vaccine sequence in CD4 binding site-overlapping signature k-mers identified in the HVTN 505 sieve analysis.** The dashed black curves represent 95% pointwise confidence intervals. The horizontal box plots at the top show distributions of the PC-weighted Hamming distances in primary endpoint cases in the vaccine group (open red triangles, n=53) and the placebo group (open blue circles, n=63), where the vertical line in each boxplot represents the median PC-weighted Hamming distance and the boxes extend through the 25^th^ and 75^th^ percentiles. “Unadjusted Sieve P” is a p‑value from testing (Wald test) whether VE varies with the sequence distance on the x-axis.

**Figure S12.** **Shannon entropy for the set of CD4 binding site-overlapping signature k-mers in the vaccine and placebo groups, and a set of matched reference sequences selected from the LANL HIV Sequence database.** The vertical dashed segments are 95% bootstrap confidence intervals. n=94 LANL reference sequences, n=63 HVTN 705 placebo sequences, n=53 HVTN 705 vaccine sequences).

**Figure S13.** **Vaccine efficacy (VE) against viruses with specific physicochemical (PC)-weighted Hamming distances from the C97ZA Env vaccine sequence in (A) the V2 hotspot based on the NHP challenge model, (B) the IgG3 V1V2 HVTN 705 correlates set, (C) the C1 epitope of the ADCC correlate in RV144, and (D) signature CD4bs antibody contact sites in HVTN 505.** The dashed black curves represent 95% pointwise confidence intervals. The horizontal box plots at the top show distributions of the PC-weighted Hamming distances in primary endpoint cases in the vaccine group (open red triangles, n=53) and the placebo group (open blue circles, n=63), where the vertical line in each boxplot represents the median PC-weighted Hamming distance and the boxes extend through the 25^th^ and 75^th^ percentiles. “Unadjusted Sieve P” is a p-value from testing (Wald test) whether VE varies with the sequence distance on the x-axis.

**Figure S14.** **Vaccine efficacy (VE) against viruses with specific physicochemical (PC)-weighted Hamming distance from the gp70-001428.2.42 V1V2 antigen sequence in the IgG3 V1V2 correlates set.** The dashed black curves represent 95% pointwise confidence intervals. The horizontal box plots at the top show distributions of the PC-weighted Hamming distance in primary endpoint cases in the vaccine group (open red triangles, n=53) and the placebo group (open blue circles, n=63), where the vertical line in each boxplot represents the median PC-weighted Hamming distance and the boxes extend through the 25^th^ and 75^th^ percentiles. “Unadjusted Sieve P” is a p-value from testing (Wald test) whether VE varies with the sequence distance on the x-axis.

**Figure S15**. **Vaccine efficacy (VE) against viruses with specific (A) length of V1V2, (B) number of PNGS motifs in V1V2, (C) electrochemical charge of V2, and (D) cysteine count in gp160.** The dashed black curves represent 95% pointwise confidence intervals. The horizontal box plots at the top show distributions of each viral geometry feature in primary endpoint cases in the vaccine group (open red triangles, n=53) and the placebo group (open blue circles, n=63), where the vertical line in each boxplot represents the median PC-weighted Hamming distance and the boxes extend through the 25^th^ and 75^th^ percentiles. “Two-Sided Unadjusted Sieve P” is a p-value from testing (Wald test) whether VE varies with the feature on the x-axis.

**A**

**B**

**Figure S16.** **Vaccine efficacy (VE) against viruses with specific (A) number of PNGS motifs in the V5 loop, and (B) length of the V5 loop.** In panel A, filled squares designate VE point estimates, and bars extend through the 95% confidence intervals (CIs). The dashed black curves in panel B represent 95% pointwise confidence intervals. The horizontal box plots at the top show distributions of the length of V5 in primary endpoint cases in the vaccine group (open red triangles, n=53) and the placebo group (open blue circles, n=63) where the vertical line in each boxplot represents the median V5 loop length and the boxes extend through the 25^th^ and 75^th^ percentiles. Due to the skewed distribution and the discrete nature of the variable, the median and Q3 overlap. “Two-Sided Unadjusted Sieve P” is a p-value from testing (Wald test) whether VE varies with the length of V5.

**Figure S17. Vaccine efficacy (VE) by single vs. multiple early virus lineages.** Filled squares designate VE point estimates, and bars extend through the 95% confidence intervals (CIs). Two-sided p-values were from two-sided Wald tests. Two-sided differential VE p-values were obtained using the Lunn-McNeil test.^4^

**Figure S18. Mean differences (vaccine minus placebo) in standardised template modelling (TM)-scores of 15-mers tiled across gp120 and gp41 vs. unadjusted two-sided P-values (permutation test) for a treatment difference.** AlphaFold3^5^ was used to predict the structure of gp120 (HXB2 positions 31–511) and of a region of gp41 (HXB2 positions 512–665), for the observed mindist Env sequence of each primary endpoint (n=116) and for the C97ZA vaccine-insert Env sequence. TM-scores, which correspond to a topological similarity metric for comparing each predicted primary endpoint structure to the predicted structure of each vaccine sequence, were then calculated. The TM-scores were calculated using overlapping 15-mer windows (n=115 windows) across gp120, with a 4-position overlap between consecutive windows. Windows with any hypervariable loop positions were excluded. A minimum threshold of six amino acids per window was required to avoid spuriously large differences dominated by point mutations. TM-scores were pre-standardised to the [0, 1] interval. Permutation test P-values were based on linear regression models adjusted for baseline age, BMI, HIV risk score, and the indicator of South Africa, calculated for all 15-mers overlapping by 4 amino acid positions. TM, template modelling.

**Figure S19.** **For each of the 116 primary endpoint cases with available sequence data (rows), proportion of intra-host sequences with presence of a particular residue (or PNGS) at an Env amino acid position, for 144 screened-in hypothesis-driven features analysed in tier 1 (columns).** Zero proportion is shown in white, and proportion equal to 1 is shown in darkest blue. Sequence features (columns) are ordered by the ascending proportion of primary endpoints with a displayed value >0 and <1. Primary endpoints (rows) are ordered from bottom to top by the ascending proportion of sequence features with a displayed value >0 and <1.

**Figure S20.** **For each of the 116 primary endpoint cases with available sequence data (rows), proportion of intra-host sequences with the value of a hypothesis-driven tier 1 quantitative feature (columns) outside the interval defined as the host’s modal value –/+ 0.5 times the average of intra-host sample standard deviations.** Zero proportion is shown in white. Sequence features (columns) are ordered by the ascending average displayed value over all primary endpoints. Primary endpoints (rows) are ordered from bottom to top by the ascending average displayed value over all sequence features.

**Table S6. Numbers of binary features that met the inter- and intra-host diversity screening criteria individually and combined for the multi-sequence sieve analysis**

| Binary Feature Type | Feature Prevalence Threshold | Sufficient  Inter-host  Diversity | Sufficient  Intra-host  Diversity | Sufficient Inter- and Intra-host  Diversity |
| --- | --- | --- | --- | --- |
| Tier 1 residue presence/absence | 1% | 320 | 0 | 0 |
|  | 99% | 305 | 0 | 0 |
| Tier 1 PNGS motif presence/absence | 1% | 7 | 0 | 0 |
|  | 99% | 8 | 0 | 0 |
| Tier 2 residue presence/absence | 1% | 2,194 | 71 | 71 |
|  | 99% | 2,105 | 43 | 43 |

**Figure S21. Multi-sequence and single-sequence sieve analyses of Env position 832.** (A) Vaccine efficacy (VE) against viral populations with ≥99% vs. <99% prevalence of leucine at Env position 832 (L832) after exclusion of sequences having a gap at position 832, contrasted with VE against viral populations whose mindist sequence carries L832 vs. notL832 after exclusion of sequences with a gap at position 832. In the multi-sequence analysis, case counts reflect sampled sequences only. Filled squares designate VE point estimates, and bars extend through the 95% confidence intervals (CIs). (B) L832 prevalence among sampled sequences from primary endpoints by treatment group (n=53 in the vaccine group, n=63 in the placebo group). Data point size is proportional to sequencing depth. The solid vertical lines show the average treatment-specific L832 prevalence.

**Table S7. Unadjusted p-values from the DiProPerm analysis of 97 sequence feature subsets, testing for differences in high-dimensional distributions of sequence features between vaccine and placebo primary endpoints**

| **Feature Subset** | **P-value** |  | **Feature Subset** | **P-value** |  | **Feature Subset** | **P-value** |
| --- | --- | --- | --- | --- | --- | --- | --- |
| Subset 2e | 0.029 |  | Subset 8b Hybrid | 0.525 |  | Subset 8 Hybrid | 0.684 |
| Subset 2e Hybrid | 0.035 |  | Subset 12a Hybrid | 0.526 |  | Subset 12 Hybrid | 0.688 |
| Subset 4e | 0.085 |  | Subset 11a Hybrid | 0.528 |  | Subset 8 | 0.701 |
| Subset 4e Hybrid | 0.097 |  | Subset 13h | 0.531 |  | Subset 12b | 0.703 |
| Subset 6 | 0.171 |  | Subset 8b | 0.535 |  | Subset 14 (All Features Combined) | 0.716 |
| Subset 6 Hybrid | 0.209 |  | Subset 8a Hybrid | 0.535 |  | Subset 12 | 0.733 |
| Subset 6f | 0.212 |  | Subset 11b Hybrid | 0.536 |  | Subset 5 Hybrid | 0.763 |
| Subset 6d | 0.237 |  | Subset 6a | 0.537 |  | Subset 5b Hybrid | 0.773 |
| Subset 6f Hybrid | 0.267 |  | Subset 13c | 0.544 |  | Subset 10 Hybrid | 0.782 |
| Subset 6d Hybrid | 0.300 |  | Subset 6b Hybrid | 0.545 |  | Subset 10 | 0.783 |
| Subset 13l | 0.356 |  | Subset 7 Hybrid | 0.545 |  | Subset 5 | 0.788 |
| Subset 13e | 0.407 |  | Subset 8c Hybrid | 0.546 |  | Subset 2d Hybrid | 0.794 |
| Subset 11c | 0.419 |  | Subset 12a | 0.547 |  | Subset 5b | 0.799 |
| Subset 4c Hybrid | 0.433 |  | Subset 11a | 0.551 |  | Subset 2d | 0.829 |
| Subset 11c Hybrid | 0.437 |  | Subset 6c Hybrid | 0.553 |  | Subset 2 Hybrid | 0.832 |
| Subset 13d | 0.438 |  | Subset 13i | 0.554 |  | Subset 2 | 0.835 |
| Subset 13a | 0.441 |  | Subset 13j | 0.555 |  | Subset 3 Hybrid | 0.881 |
| Subset 2c Hybrid | 0.446 |  | Subset 11b | 0.558 |  | Subset 4d Hybrid | 0.896 |
| Subset 4c | 0.449 |  | Subset 8a | 0.558 |  | Subset 3 | 0.898 |
| Subset 2c | 0.456 |  | Subset 13m | 0.575 |  | Subset 4d | 0.921 |
| Subset 13n | 0.464 |  | Subset 13k | 0.579 |  | Subset 9 | 0.924 |
| Subset 6e Hybrid | 0.469 |  | Subset 6b | 0.580 |  | Subset 9 Hybrid | 0.925 |
| Subset 13p | 0.474 |  | Subset 7 | 0.580 |  | Subset 4 Hybrid | 0.941 |
| Subset 6e | 0.476 |  | Subset 8c | 0.591 |  | Subset 4 | 0.944 |
| Subset 8d Hybrid | 0.476 |  | Subset 6c | 0.599 |  | Subset 4b Hybrid | 0.975 |
| Subset 8d | 0.482 |  | Subset 8e | 0.601 |  | Subset 4b | 0.980 |
| Subset 11 Hybrid | 0.485 |  | Subset 13b | 0.602 |  | Subset 2b Hybrid | 0.989 |
| Subset 11 | 0.498 |  | Subset 5a Hybrid | 0.613 |  | Subset 2a Hybrid | 0.990 |
| Subset 13o | 0.498 |  | Subset 8e Hybrid | 0.614 |  | Subset 2a | 0.991 |
| Subset 1 (Baseline Control Variables Only) | 0.507 |  | Subset 13g | 0.619 |  | Subset 4a Hybrid | 0.995 |
| Subset 13f | 0.520 |  | Subset 5a | 0.641 |  | Subset 2b | 0.997 |
| Subset 6a Hybrid | 0.521 |  | Subset 12b Hybrid | 0.668 |  | Subset 4a | 1.000 |

**Figure S22. Variable loadings from the Direction–Projection–Permutation (DiProPerm) analysis for subset 2e, which included 11 vaccine-residue match/mismatch features at signature positions for clade C broadly neutralising antibodies (bnAbs).** Numbers denote Env amino acid positions in HXB2 coordinates. ‘Risk’ indicates baseline HIV risk score, ‘RSA’ indicates enrolment in South Africa, and ‘Age’ and ‘BMI’ were measured at enrolment. Loadings quantify each feature’s contribution to the mean difference direction separating vaccine from placebo endpoints.

**Supplementary References**

1.	Rolland M, Edlefsen PT, Larsen BB, et al. Increased HIV-1 vaccine efficacy against viruses with genetic signatures in Env V2. *Nature* 2012; **490**(7420): 417-20.

2.	deCamp AC, Rolland M, Edlefsen PT, et al. Sieve analysis of breakthrough HIV-1 sequences in HVTN 505 identifies vaccine pressure targeting the CD4 binding site of Env-gp120. *PLoS One* 2017; **12**(11): e0185959.

3.	Adler D, Kelly ST. vioplot: violin plot. R package version 0.5.1 <https://github.com/TomKellyGenetics/vioplot>. 2025.

4.	Lunn M, McNeil D. Applying Cox regression to competing risks. *Biometrics* 1995; **51**(2): 524-32.

5.	Bai H, Lewitus E, Li Y, et al. Contemporary HIV-1 consensus Env with AI-assisted redesigned hypervariable loops promote antibody binding. *Nat Commun* 2024; **15**(1): 3924.
